# The global burden of breast cancer in women from 1990 to 2030: assessment and projection based on the global burden of disease study 2019

**DOI:** 10.3389/fonc.2024.1364397

**Published:** 2024-06-20

**Authors:** Song Zhang, Zhihui Jin, Lingling Bao, Peng Shu

**Affiliations:** ^1^ Department of Hematology and Oncology, Ningbo Beilun District People’s Hospital, Ningbo, China; ^2^ Precision Medicine Research Center, Ningbo Beilun District People’s Hospital, Ningbo, China

**Keywords:** global burden of disease, breast cancer in women, projection, generalized additive models, age-standardized disability-adjusted life year (DALY) rate, age-standardized incidence rate (ASIR), estimated annual percentage change

## Abstract

**Background and aim:**

This study aims to analyze the worldwide prevalence, mortality rates, and disability-adjusted life years (DALYs) attributed to breast cancer in women between 1990 and 2019. Additionally, it seeks to forecast the future trends of these indicators related to the burden of breast cancer in women from 2020 to 2030.

**Methods:**

Data from the Global Burden of Disease Study (GBD) 2019 was analyzed to determine the age-standardized incidence rate (ASIR) and age-standardized death rate (ASDR) of DALYs due to breast cancer in women across 204 countries and territories from 1990 to 2019. Socio-economic development levels of countries and regions were assessed using Socio-demographic Indexes, and trends in the burden of breast cancer in women worldwide from 2020 to 2030 were projected using generalized additive models (GAMs).

**Results:**

The estimated annual percentage change (EAPC) in the ASIR breast cancer in women globally was 0.36 from 1990 to 2019 and is expected to increase to 0.44 from 2020 to 2030. In 2019, the ASIR of breast cancer in women worldwide was 45.86 and is projected to reach 48.09 by 2030. The burden of breast cancer in women generally rises with age, with the highest burden expected in the 45–49 age group from 2020 to 2030. The fastest increase in burden is anticipated in Central sub-Saharan Africa (EAPC in the age-standardized death rate: 1.62, EAPC in the age-standardized DALY rate: 1.52), with the Solomon Islands (EAPC in the ASIR: 7.25) and China (EAPC in the ASIR: 2.83) projected to experience significant increases. Furthermore, a strong positive correlation was found between the ASIR breast cancer in women globally in 1990 and the projected rates for 2030 (r = 0.62).

**Conclusion:**

The anticipated increase in the ASIR of breast cancer in women globally by 2030 highlights the importance of focusing on women aged 45–49 in Central sub-Saharan Africa, Oceania, the Solomon Islands, and China. Initiatives such as breast cancer information registries, raising awareness of risk factors and incidence, and implementing universal screening programs and diagnostic tests are essential in reducing the burden of breast cancer and its associated morbidity and mortality.

## 1 Introduction

Breast cancer is the leading cause of cancer-related deaths and the most prevalent cancer among women globally, presenting a significant public-health challenge (1, 2). According to the World Health Organization, there were around 2.26 million new cases of breast cancer in 2020, making it the most common cancer worldwide (3). Additionally, breast cancer incidence and mortality are on the rise across various socio-economic levels, which is a major concern. In comparison to low-income countries, high-income countries have higher incidence rates but lower mortality rates from breast cancer (4).Disparities in breast cancer mortality rates across countries can be attributed to factors such as improved control of risk factors in high-income nations, access to educational resources, increased adherence to screening and surveillance programs for early disease detection. Furthermore, high-income countries typically have better access to basic health services and a greater number of public cancer centers providing advanced diagnosis and treatment options. Future efforts to address the burden of breast cancer include screening for risk factors, monitoring global data, promoting early detection, offering health guidance, and implementing enhanced interventions and treatments to reduce disease risk (5, 6).

The burden of breast cancer is higher in high-income countries due to lifestyle factors and large populations (7). On the other hand, changing reproductive patterns, lack of awareness, delayed diagnosis, and treatment worsen the burden in middle- and low-income countries (1, 8). Furthermore,. Particularly worrisome is the burden of breast cancer in women aged 15–49, as this age group has been less studied and their health impacts not only their own lives but also those of their families and society at large (9).

Given the significant impact of breast cancer on countries globally, it is crucial to forecast future burdens to enhance the allocation of public health resources and early interventions for prevention and control in high-burden countries (10–13). However, literature lacks comprehensive projections of breast cancer burdens worldwide (14). Therefore, this study aimed to refine projections from 2020 to 2030 using data from the Global Burden of Disease Study (GBD)2019. By employing generalized additive models (GAMs), we integrated linear predictability and non-linear trends to project the burden of breast cancer in women globally. These findings can assist in identifying high-risk groups and developing targeted prevention and intervention strategies, ultimately contributing to global health efforts in combating breast cancer among women.

## 2 Materials and methods

### 2.1 Data source

The GBD is a comprehensive global epidemiology program that involves collaboration among over 3,600 experts from 145 countries. It assesses the impact of diseases, injuries, and risk factors worldwide, at both regional and national levels (15, 16). By analyzing a vast amount of published literature, surveys, and epidemiological data, the GBD offers detailed insights into the incidence, prevalence, mortality, and Disability-Adjusted Life Years (DALYs) related to over 350 diseases in 204 countries and territories. Its annual updates reflect the changing landscape of global health and help in forecasting future health service needs (17, 18).

One of the key factors in our study was the Socio-demographic Index (SDI), a comprehensive measure that evaluates the social and economic development of a region or country based on per capita income, average years of schooling, and fertility rates. The SDI enables the classification of countries and regions into different categories: low-SDI (0–0.45), low–medium-SDI (0.45–0.60), medium-SDI (0.60–0.68), high-SDI (0.68–0.80), and very-high-SDI (0.80–1). This classification system offers a detailed insight into the socio-economic status of a country or region, with low-SDI areas characterized by lower income and education levels, as well as higher fertility rates, while very-high-SDI regions exhibit higher income and educationlevels, along with lower fertility rates (19).

We utilized the coding system of the 9th and 10th revisions of the International Classification of Diseases (ICDs) to ensure that only confirmed cases breast cancer were included. In ICD-9, the codes for breast cancer range from 174 to 175.9, 217 to 217.8, 233.0, 238.3, 239.3, and 610 to 610.9. In ICD-10, they are categorized as C50 to C50.9, D05 to D05.9, D24 to D24.9, D48.6, and D49.3. These codes were meticulously cross-referenced with the list of causes in the GBD 2019, ensuring our data analysis was both comprehensive and specific.

### 2.2 Statistical analysis

A comprehensive statistical analysis was conducted to project trends in the incidence, mortality, and DALYs related to breast cancer in women globally from 2020 to 2030. Initially, a logarithmic model was applied using population data from 2009 to 2019 for each country to estimate population changes.


ln(pnum)=a×year+b

where “*pnum*” is the population size; *a* is the coefficient of the year, which is the calendar year; and *b* is the intercept. We utilized GAMs to forecast the number of women affected by breast cancer mortality, as well as the DALYs attributed to breast cancer, on a global scale from 2020 to 2030. GAMs offer the advantage of incorporating non-linear relationships of predictor variables, making them more advanced compared to conventional linear models. The specific definition of the GAM used in our analysis was as follows:


ln(number)=s[ln(pnum)]+s(c)+s(year)+s(e)+r

where “*number*” is the number of cases breast cancer in women, the number of deaths from breast cancer in women, or the number of DALYs due to breast cancer in women; “*pnum*” is the population size; *c* is the median age in each age group of women; “*year*” is the calendar year; *e* is the difference between the calendar year and the mid-value of the age group of women; *s* is a smoothing spline function essential for capturing the nuanced complexities within the data; and r is the intercept (20).

The bootstrap method was utilized to calculate projections and their 95% confidence intervals (CIs). This approach helped ensure the precision and reliability of the projections, which was essential for evaluating their variability and accuracy.

Temporal trends were assessed by calculating age-standardized rates of breast cancer in women and estimating the annual percentage changes (EAPCs) in these rates. The EAPC and 95% CIs of each parameter were analyzed to identify any increasing, decreasing, or stable trends (21–23).

Scatter plots were utilized to illustrate the relationships between age-standardized incidence rate (ASIRs) and age-standardized death rate (ASDRs) of age-standardized DALY rates attributed to breast cancer in women across different SDI quintiles. This visual examination offered valuable insights into the associations between demographic variables and the burden of breast cancer in women. The statistical analysis was conducted using R software (version 3.6.1). A significance level of P< 0.05 was considered to indicate statistically significant differences, thereby enhancing the strength and credibility of our results.

## 3 Results

### 3.1 Projected trends in the global burden of breast cancer in women from 2020 to 2030

**Figure 1:**
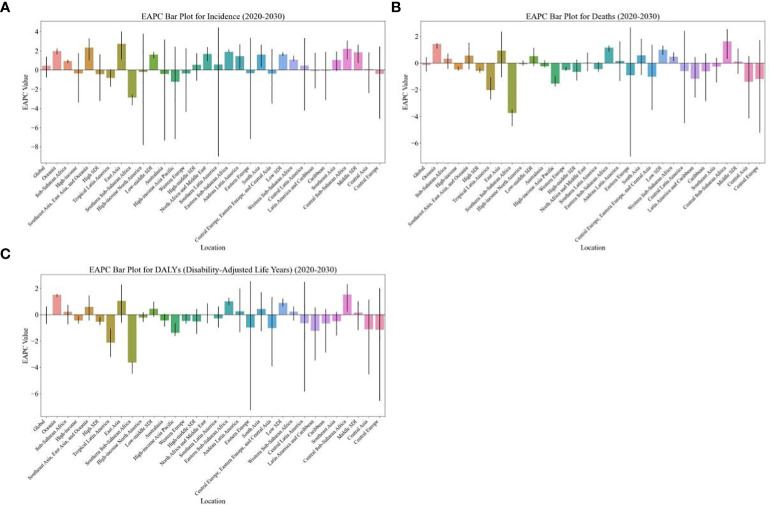
EAPC of global burden of breast cancer in women from 2020 to 2030, by regions. **(A)** ASIR **(B)** ASDR **(C)** age-standardized DALY rate. EAPC, estimated annual percentage change; ASIR, age standardized incidence rate; ASDR, age standardized death rate.

**Figure 2:**
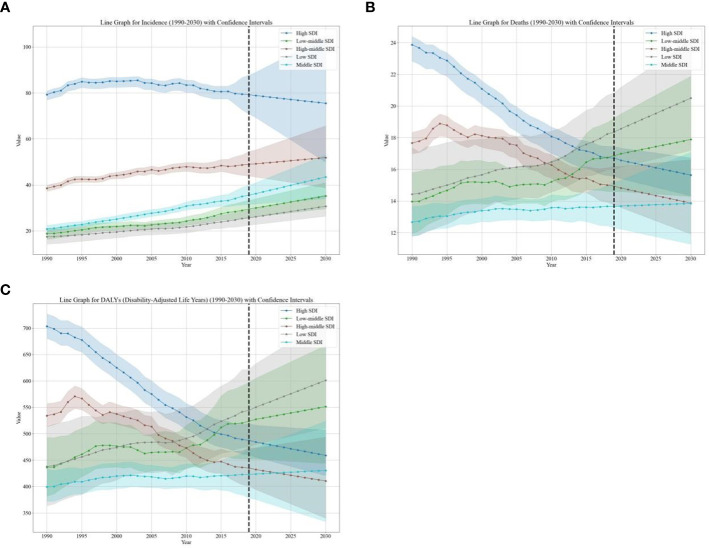
EAPC of global burden of breast cancer in women from 1990 to 2030, by SDI Regions. **(A)** The EAPC of ASIR **(B)** The EAPC of ASDR **(C)** The EAPC of age-standardized DALY rate. EAPC, estimated annual percentage change; ASIR, age standardized incidence rate; ASDR, age standardized death rate.

The global burden of breast cancer in women is expected to rise significantly from 2020 to 2030, surpassing the increase observed from 1990 to 2019(**Table 1**; **Figure 1**). Specifically, there is a projected notable increase in the ASIRof breast cancer in women, with the EAPC rising from 0.36 (95% CI: 0.25, 0.47) in 1990–2019 to 0.44 (95% CI: 0.40, 1.39) in 2020–2030 (**Table 1**; **Figure 1**). This translates to an increase from 45.86 (95% uncertainty interval [UI]: 41.91, 49.76) cases per 100,000 women in 2019 to 48.09 (95% UI: 38.55, 57.89) cases per 100,000 women in 2030 (**Table 2**; **Figure 2**). However, the projected increases in the age-standardized DALY rate and ASDR due to breast cancer in women are expected to be less pronounced (EAPC: 0.01 [95% CI:0.01, 0.61] and EAPC: -0.15, [95% CI: -0.65, -0.17], respectively) (**Table 2**; **Figure 2**). In contrast, when considering the baseline burden of breast cancer in women globally, the age-standardized DALY rate is anticipated to decrease from 473.83 (95% UI: 437.30, 510.51) in 2019 to 474.28 (95% UI: 405.49, 545.70) in 2030 per 100,000 women, and the ASDR is projected to decrease from 15.88 (95% UI: 14.66, 17.07) in 2019 to 15.62 (95% UI: 13.63, 17.89) in 2030 per 100,000 women, indicating a slightly negative trend (**Supplementary Table 4**; **Figure 2**).

**Table 1:** The EAPC of breast cancer in women from 1990 to 2019 in different regions.

location	1990–2019			2020–2030		
	No.(95%CI)	No.(95%CI)	No.(95%CI)	No.(95%CI)	No.(95%CI)	No.(95%CI)
	Age-standardized DALY rate (per 100000)	ASDR	ASIR	Age-standardized DALY rate (per 100000)	ASDR	ASIR
Global	-0.50 (-0.58, -0.44)	-0.51 (-0.61, -0.44)	0.36 (0.25, 0.47)	0.01 (-0.70, 0.61)	-0.15 (-0.65, 0.45)	0.44 (-0.78, 1.39)
**Sociodemographic index**	–	–	–	–	–	–
High SDI	-1.41 (-1.49, -1.32)	-1.31 (-1.40, -1.23)	-0.12 (-0.25, 0.02)	-0.53 (-0.79, -0.18)	-0.58 (-0.73, -0.36)	-0.43 (-3.22, 1.62)
High-middle SDI	-0.96 (-1.06, -0.86)	-0.79 (-0.91, -0.69)	0.74 (0.57, 0.90)	-0.51 (-1.46, 0.43)	-0.66 (-1.30, 0.26)	0.54 (-1.13, 1.75)
Middle SDI	0.13 (0.08, 0.16)	0.20 (0.15, 0.23)	1.87 (1.78, 1.94)	0.16 (-1.17, 1.03)	0.12 (-0.80, 1.09)	1.83 (0.71, 2.62)
Low-middle SDI	0.47 (0.42, 0.50)	0.50 (0.54, 0.48)	1.44 (1.44, 1.44)	0.45 (-0.18, 0.98)	0.52 (-0.22, 1.15)	1.59 (1.23, 1.89)
Low SDI	0.67 (0.85, 0.53)	0.73 (0.99, 0.51)	1.30 (1.55, 1.12)	0.89 (0.64, 1.22)	0.99 (0.64, 1.30)	1.62 (1.47, 1.84)
**Regions**	–	–	–	–	–	–
Andean Latin America	-0.36 (-0.59, -0.17)	-0.22 (-0.41, -0.05)	1.35 (1.10, 1.55)	0.26 (-1.35, 1.97)	0.16 (-1.35, 1.65)	1.43 (0.08, 2.67)
Australasia	-1.72 (-1.82, -1.59)	-1.55 (-1.69, -1.44)	-0.26 (-0.58, 0.05)	-0.44 (-0.89, 0.07)	-0.24 (-0.35, 0.21)	-0.42 (-7.35, 3.16)
Caribbean	0.27 (-0.09, 0.62)	0.28 (0.01, 0.58)	1.01 (0.74, 1.27)	-0.66 (-2.88, 0.43)	-0.63 (-2.84, 0.73)	-0.08 (-3.16, 1.88)
Central Asia	-0.59 (-0.71, -0.48)	-0.23 (-0.33, -0.13)	0.33 (0.23, 0.43)	-1.12 (-4.53, 1.13)	-1.40 (-4.15, 0.52)	0.08 (-2.43, 1.83)
Central Europe	-0.61 (-0.78, -0.45)	-0.35 (-0.52, -0.21)	0.93 (0.76, 1.09)	-1.15 (-6.53, 1.99)	-1.19 (-5.23, 1.71)	-0.42 (-5.08, 2.43)
Central Europe, Eastern Europe, and Central Asia	-0.76 (-0.83, -0.65)	-0.44 (-0.54, -0.33)	0.71 (0.63, 0.82)	-1.03 (-3.91, 1.34)	-1.02 (-3.52, 1.36)	-0.40 (-3.50, 2.17)
Central Latin America	0.13 (-0.08, 0.35)	0.12 (-0.07, 0.33)	1.55 (1.34, 1.77)	-0.65 (-5.85, 2.48)	-0.60 (-4.49, 2.42)	0.46 (-4.24, 3.32)
Central Sub-Saharan Africa	0.56 (0.32, 0.75)	0.67 (0.41, 0.92)	1.02 (0.77, 1.23)	1.52 (0.22, 2.32)	1.62 (0.33, 2.54)	2.21 (1.10, 3.04)
East Asia	-0.29 (-0.29, -0.27)	-0.10 (-0.15, -0.05)	2.67 (2.61, 2.75)	1.05 (-0.61, 2.28)	0.93 (-1.06, 2.35)	2.73 (1.09, 4.00)
Eastern Europe	-0.90 (-1.00, -0.77)	-0.61 (-0.71, -0.49)	0.68 (0.60, 0.80)	-0.99 (-7.24, 2.54)	-0.91 (-7.00, 2.67)	-0.34 (-7.19, 3.34)
Eastern Sub-Saharan Africa	0.29 (0.44, 0.18)	0.55 (0.74, 0.38)	0.93 (1.13, 0.79)	1.01 (0.74, 1.29)	1.17 (0.85, 1.32)	1.89 (1.86, 2.15)
High-income	-1.41 (-1.49, -1.33)	-1.33 (-1.44, -1.26)	-0.11 (-0.25, 0.04)	-0.45 (-0.68, -0.13)	-0.46 (-0.56, -0.27)	-0.35 (-3.40, 1.76)
High-income Asia Pacific	0.39 (0.27, 0.50)	0.56 (0.37, 0.67)	2.15 (2.01, 2.26)	-1.38 (-1.61, -0.66)	-1.53 (-1.76, -0.96)	-1.22 (-7.20, 2.42)
High-income North America	-1.74 (-1.80, -1.68)	-1.56 (-1.63, -1.50)	-0.95 (-1.12, -0.75)	-0.23 (-0.55, 0.17)	-0.08 (-0.19, 0.20)	-0.20 (-7.83, 3.75)
Latin America and Caribbean	-0.25 (-0.37, -0.14)	-0.25 (-0.35, -0.17)	1.03 (0.91, 1.15)	-1.23 (-3.48, 0.55)	-1.18 (-2.58, 0.44)	-0.09 (-1.92, 1.78)
North Africa and Middle East	0.30 (0.23, 0.30)	0.38 (0.33, 0.36)	1.15 (1.09, 1.14)	0.00 (-0.64, 0.85)	0.03 (-0.61, 0.78)	1.66 (0.91, 2.39)
Oceania	0.95 (0.90, 1.05)	0.95 (0.89, 1.04)	1.27 (1.19, 1.36)	1.49 (1.32, 1.57)	1.42 (1.08, 1.49)	1.96 (1.62, 2.23)
South Asia	0.34 (0.38, 0.35)	0.30 (0.40, 0.26)	0.77 (0.84, 0.76)	0.42 (-1.25, 1.70)	0.58 (-0.89, 1.81)	1.58 (0.30, 2.63)
Southeast Asia	-0.07 (-0.16, -0.05)	-0.01 (-0.11, 0.03)	1.15 (1.08, 1.18)	-0.49 (-1.59, 0.21)	-0.27 (-1.44, 0.38)	1.04 (-0.13, 1.90)
Southeast Asia, East Asia, and Oceania	-0.13 (-0.13, -0.14)	-0.03 (-0.06, -0.01)	2.22 (2.14, 2.30)	0.58 (-0.43, 1.44)	0.55 (-0.46, 1.52)	2.32 (1.00, 3.28)
Southern Latin America	-0.85 (-0.92, -0.78)	-0.73 (-0.82, -0.67)	0.40 (0.09, 0.70)	-0.29 (-0.98, 0.61)	-0.44 (-0.67, 0.10)	0.57 (-9.01, 4.43)
Southern Sub-Saharan Africa	0.77 (0.73, 0.87)	0.93 (1.00, 0.87)	1.27 (1.29, 1.30)	-3.63 (-4.50, -3.57)	-3.73 (-4.73, -3.47)	-2.86 (-3.67, -2.54)
Sub-Saharan Africa	0.63 (0.60, 0.64)	0.79 (0.83, 0.74)	1.23 (1.23, 1.23)	0.22 (-0.73, 0.73)	0.33 (-0.43, 0.70)	0.93 (0.69, 1.05)
Tropical Latin America	-0.54 (-0.58, -0.51)	-0.58 (-0.65, -0.55)	0.71 (0.66, 0.74)	-2.13 (-3.22, -1.05)	-2.01 (-2.72, -1.07)	-0.84 (-1.76, 0.05)
Western Europe	-1.58 (-1.67, -1.47)	-1.39 (-1.52, -1.30)	0.07 (-0.15, 0.27)	-0.48 (-0.68, 0.04)	-0.51 (-0.58, -0.29)	-0.36 (-4.37, 2.26)
Western Sub-Saharan Africa	0.85 (0.79, 0.84)	0.96 (0.95, 0.88)	1.48 (1.41, 1.47)	0.24 (-0.46, 0.62)	0.47 (0.14, 0.79)	1.08 (0.91, 0.67)

**Table 2:** The Age-standerized rates of breast cancer in women from 1990 to 2019 in different regions.

location	1990			2019			2030		
	No.(95%UI)	No.(95%UI)	No.(95%UI)	No.(95%UI)	No.(95%UI)	No.(95%UI)	No.(95%UI)	No.(95%UI)	No.(95%UI)
	Age-standardized DALY rate (per 100000)	ASDR	ASIR	Age-standardized DALY rate (per 100000)	ASDR	ASIR	Age-standardized DALY rate (per 100000)	ASDR	ASIR
**Global**	524.87 (501.78, 551.15)	17.76 (16.93, 18.51)	40.12 (38.78, 41.33)	473.83 (437.30, 510.51)	15.88 (14.66, 17.07)	45.86 (41.91, 49.76)	474.28 (405.49, 545.70)	15.62 (13.63, 17.89)	48.09 (38.55, 57.89)
**Sociodemographic index**	–	–	–	–	–	–	–	–	–
High SDI	703.06 (680.17, 726.62)	23.87 (22.83, 24.39)	79.30 (77.00, 80.87)	487.45 (459.76, 518.84)	16.71 (15.56, 17.45)	79.22 (70.83, 87.70)	459.04 (420.84, 507.02)	15.64 (14.32, 16.73)	75.48 (49.86, 104.53)
High-middle SDI	534.27 (513.62, 557.28)	17.66 (16.98, 18.33)	38.52 (37.22, 39.86)	434.96 (400.69, 473.31)	14.93 (13.75, 16.19)	48.93 (43.84, 54.49)	410.57 (340.24, 493.65)	13.87 (11.92, 16.61)	51.87 (38.72, 65.71)
Low SDI	437.94 (363.58, 513.68)	14.42 (11.76, 17.37)	17.43 (14.22, 20.52)	544.03 (475.61, 621.56)	18.34 (15.98, 20.84)	25.67 (22.54, 29.10)	601.03 (511.11, 714.33)	20.50 (17.19, 24.11)	30.70 (26.38, 35.69)
Low-middle SDI	436.47 (381.96, 493.02)	13.97 (11.93, 15.79)	18.80 (16.33, 21.13)	523.52 (452.09, 597.21)	16.86 (14.59, 19.24)	29.47 (25.91, 33.20)	551.28 (443.94, 667.19)	17.88 (14.28, 21.88)	35.14 (29.57, 41.00)
Middle SDI	399.52 (372.02, 433.14)	12.67 (11.79, 13.68)	20.81 (19.25, 22.45)	422.91 (378.62, 468.87)	13.66 (12.30, 15.18)	35.52 (31.47, 39.81)	430.58 (333.72, 524.17)	13.85 (11.28, 17.08)	43.43 (34.03, 53.08)
**Regions**	–	–	–	–	–	–	–	–	–
Andean Latin America	385.28 (345.28, 431.27)	12.73 (11.44, 14.21)	19.22 (17.23, 21.54)	370.35 (300.19, 459.51)	12.67 (10.44, 15.51)	29.63 (24.05, 36.45)	380.78 (257.43, 572.58)	12.89 (8.96, 18.61)	34.74 (24.12, 49.26)
Australasia	794.40 (763.72, 825.24)	26.46 (25.26, 27.30)	85.02 (81.28, 88.27)	509.03 (471.05, 552.78)	17.47 (16.11, 18.69)	84.69 (68.25, 104.99)	483.85 (426.36, 555.92)	16.96 (15.43, 19.04)	80.80 (29.87, 148.11)
Caribbean	596.80 (559.99, 637.90)	20.02 (18.88, 21.15)	43.15 (41.14, 45.36)	623.62 (515.19, 740.86)	20.84 (17.62, 24.40)	55.37 (46.63, 65.11)	582.17 (376.55, 783.35)	19.54 (12.94, 26.57)	55.18 (33.13, 80.27)
Central Asia	612.61 (589.89, 635.80)	18.88 (18.18, 19.56)	35.40 (34.09, 36.86)	523.88 (464.12, 588.67)	17.29 (15.53, 19.16)	38.36 (34.23, 42.80)	463.23 (282.63, 664.27)	14.84 (9.88, 20.28)	38.77 (26.40, 52.28)
Central Europe	646.94 (628.68, 664.47)	21.78 (21.10, 22.35)	46.42 (45.08, 47.66)	552.66 (476.95, 638.66)	19.87 (17.25, 22.71)	60.22 (52.04, 69.57)	487.22 (231.01, 792.66)	17.44 (9.69, 27.33)	57.56 (29.65, 90.60)
Central Latin America	372.32 (362.37, 382.23)	12.38 (11.91, 12.71)	24.06 (23.29, 24.71)	390.08 (330.64, 460.41)	12.87 (11.05, 15.09)	38.45 (32.30, 45.64)	364.46 (174.58, 603.59)	12.07 (6.78, 19.65)	40.57 (20.50, 65.47)
East Asia	295.76 (247.21, 349.22)	9.20 (7.68, 10.78)	17.23 (14.27, 20.37)	282.15 (230.81, 341.19)	9.12 (7.36, 11.13)	35.69 (28.32, 44.54)	316.48 (215.54, 439.36)	10.10 (6.57, 14.39)	48.17 (32.03, 68.66)
Eastern Europe	580.67 (563.76, 603.07)	17.93 (17.43, 18.54)	39.69 (38.55, 41.28)	529.13 (456.07, 618.57)	17.47 (15.05, 20.36)	51.89 (44.14, 61.31)	472.30 (203.32, 803.53)	15.73 (6.91, 26.81)	49.90 (20.02, 87.18)
Eastern Sub-Saharan Africa	447.71 (369.21, 527.69)	15.18 (12.37, 17.91)	17.94 (14.68, 21.14)	501.54 (427.72, 580.64)	18.15 (15.65, 20.60)	24.04 (20.78, 27.49)	561.24 (465.82, 668.23)	20.66 (17.22, 23.79)	29.61 (25.46, 34.84)
High-income Asia Pacific	295.10 (284.83, 306.71)	8.71 (8.34, 8.92)	32.74 (30.94, 34.62)	321.94 (300.10, 349.17)	9.78 (8.91, 10.41)	56.30 (47.14, 67.18)	276.04 (251.16, 323.32)	8.25 (7.33, 9.34)	49.23 (21.40, 86.21)
High-income North America	838.52 (809.43, 872.63)	27.54 (26.42, 28.19)	114.22 (110.57, 116.81)	533.82 (502.49, 569.80)	18.36 (17.28, 19.19)	93.75 (78.03, 112.64)	519.72 (472.47, 579.28)	18.12 (16.85, 19.52)	91.56 (32.89, 166.96)
North Africa and Middle East	395.45 (357.63, 458.53)	12.28 (11.03, 14.23)	19.64 (17.76, 22.61)	472.73 (409.00, 544.75)	15.22 (13.31, 17.35)	37.48 (32.68, 42.94)	473.38 (379.73, 600.10)	15.28 (12.41, 18.99)	45.05 (36.12, 55.97)
Oceania	1086.68 (862.15, 1349.77)	32.82 (26.12, 40.10)	45.27 (36.44, 55.41)	1416.87 (1084.79, 1808.02)	42.80 (33.19, 54.23)	65.58 (50.44, 83.58)	1677.81 (1260.74, 2157.31)	50.27 (37.55, 63.91)	81.78 (60.56, 107.00)
South Asia	406.15 (333.57, 466.21)	13.41 (10.60, 15.75)	17.05 (13.65, 19.79)	520.59 (426.84, 620.44)	16.83 (13.91, 20.00)	27.72 (22.91, 33.00)	545.98 (373.33, 747.51)	17.95 (12.63, 24.42)	33.01 (23.62, 44.17)
Southeast Asia	623.50 (559.91, 714.09)	18.83 (17.11, 21.29)	27.34 (24.58, 30.96)	621.22 (534.07, 719.10)	19.23 (16.62, 22.01)	38.52 (33.11, 44.64)	587.98 (447.36, 736.30)	18.66 (14.16, 22.99)	43.10 (32.64, 55.07)
Southern Latin America	794.89 (769.75, 820.70)	28.63 (27.55, 29.61)	47.99 (46.09, 50.01)	643.67 (602.30, 691.61)	24.04 (22.41, 25.61)	56.51 (43.78, 71.94)	625.30 (542.90, 739.69)	22.97 (20.87, 25.96)	60.46 (15.89, 116.74)
Tropical Latin America	521.91 (503.70, 540.25)	17.74 (16.93, 18.39)	31.73 (30.41, 32.83)	451.18 (424.36, 479.09)	15.19 (14.15, 16.11)	39.75 (37.24, 42.24)	356.58 (298.57, 425.38)	12.18 (10.52, 14.29)	36.18 (30.71, 42.29)
Western Europe	827.52 (801.86, 852.63)	28.37 (27.19, 28.98)	81.08 (78.79, 82.91)	552.56 (519.78, 591.68)	19.79 (18.32, 20.77)	85.85 (74.12, 98.85)	523.18 (480.48, 591.35)	18.67 (17.16, 20.05)	82.49 (45.62, 126.93)

**Figure 3:**
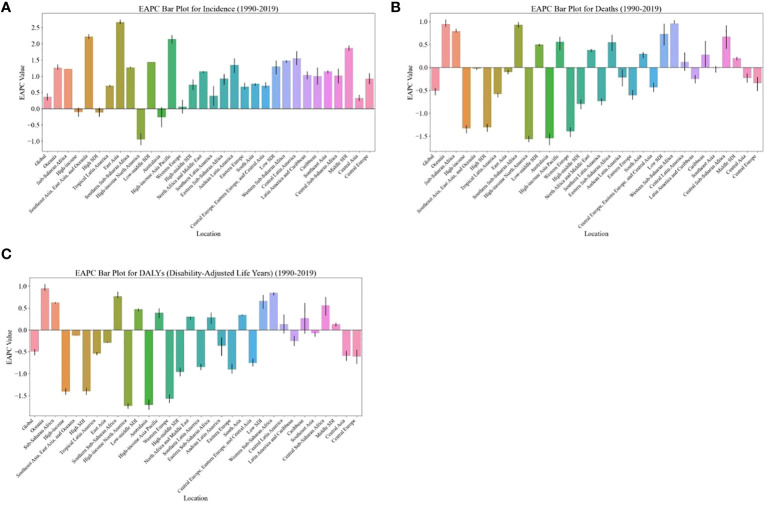
EAPC of global burden of breast cancer in women from 1990 to 2019, by regions. **(A)** ASIR **(B)** ASDR **(C)** age-standardized DALY rate. EAPC, estimated annual percentage change; ASIR, age standardized incidence rate; ASDR, age standardized death rate.

The burden of breast cancer in women is expected to vary significantly based on regions’ SDI levels. For instance, between 2020 and 2030, the ASIR of breast cancer in women is projected to decrease in high-SDI regions (EAPC: -0.43, [95% CI: -3.22, -0.47]) (**Table 1**). On the other hand, the age-standardized DALY rate and ASDR due to breast cancer in women are expected to decrease in both high-SDI regions (EAPC: -0.53, [95% CI: -0.79, -0.18] and EAPC: -0.58, [95% CI: -0.73, -0.36], respectively) and high-middle-SDI regions (EAPC: -0.51, [95% CI: -1.46, -0.56] and EAPC: -0.66, [95% CI: -1.30, -0.73], respectively) as shown in **Table 1** and **Figure 3**.

### 3.2 Projected trends in and distributions of the burden of breast cancer in women worldwide by age from 2020 to 2030

**Figure 4:**
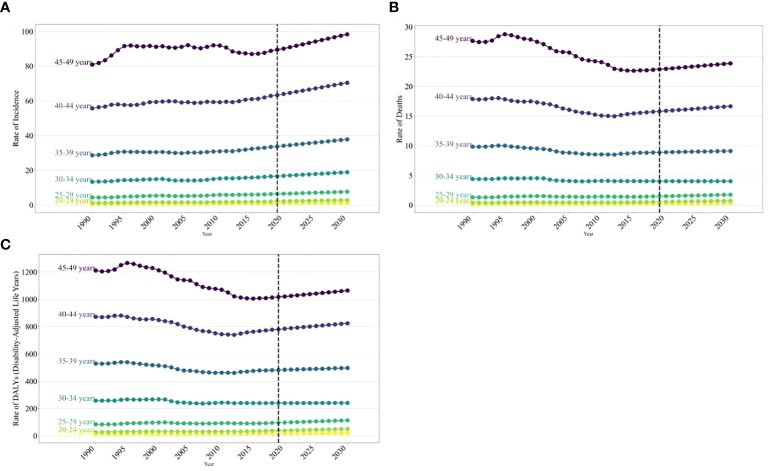
The trends of incidence rate for global burden of breast cancer in women in 2019, by SDI regions and age group. **(A)** Incidence rate **(B)** Death rate **(C)** age-standardized DALY rate.

The age distribution of the disease burden breast cancer in women worldwide is expected to vary significantly from 2020 to 2030. The ASIRs of breast cancer in women globally are projected to show a notable increase with age from 1990 to 2030. Over this timeframe, the incidence rate of breast cancer in women is anticipated to generally rise with age. Specifically, between 2020 and 2030, the incidence rate is forecasted to increase across most age groups, with the most significant rise seen in those aged 45–49, going from 80.94 (95% UI: 77.72, 84.44) in 1990 to 98.41 (95% UI: 79.55, 118.10) in 2030 per 100,000 (**Figure 4**). However, in high-SDI regions, the incidence rate of breast cancer in women aged 40–49 is projected to decline. A similar trend is expected for death rates and DALY rates due to cancer in women. Specifically, between 2020 and 2030, the death rate and the DALY rate are anticipated to increase in high-SDI and high–middle-SDI regions, particularly the death rate in those aged 40–49 and the DALY rate in those aged 45–49. Overall, the projected increase in the burden of breast cancer in those aged 40–49 is closely linked to the regions’ SDI levels (**Supplementary Tables 2** and **3**; **Supplementary Figures 2** and **3**).

In 2030, the burden of breast cancer in women is expected to rise with age, reaching its peak in the 45–49 age group. This peak will be characterized by an incidence rate of 98.41, a death rate of 23.88, and a DALY rate of 1064.40 per 100,000 individuals (**Supplementary Tables 1** and **3**; **Supplementary Figure 4**).

### 3.3 Projected distributions of the burden of breast cancer in women worldwide by region and country from 2020 to 2030

From 2020 to 2030, the highest EAPC in the ASDR and age-standardized DALY rate of breast cancer in women is projected to be in Central Sub-Saharan Africa (1.62 [95% CI: 0.33, 2.54] and EAPC: 1.52 [95% CI: 0.22, 2.32], respectively), followed by Oceania (1.42 [95% CI: 1.08, 1.49] and EAPC: 1.49 [95% CI: 1.32, 1.57], respectively (**Table 1**; **Figure 1**). Additionally, the EAPC in the ASIR of breast cancer in women is projected to be the largest in East Asia (2.73), with an increase from 35.69 in 2019 to 48.17 in 2030 per 100,000 population (**Table 2**; **Figure 1**).

Across 204 countries from 2020 to 2030, the burden of breast cancer in women is projected to vary significantly. For instance, the Solomon Islands are expected to experience the largest annual percentage changes in ASIR and ASDR, as well as age-standardized DALY rates due to breast cancer in women (7.25 [95% CI: 7.07, 7.39], 6.87 [95% CI: 6.81, 6.90], and 7.09 [95% CI: 6.93, 7.18], respectively), which are notably higher than the second-ranked country (**Supplementary Table 6**; **Supplementary Figures 5** and **7**). Furthermore, Gambia is projected to have the largest annual percentage changes in ASDRs and age-standardized DALY rates due to breast cancer in women (2.17 [95% CI: 1.18, 2.52] and 2.20 [95% CI: 1.41, 2.53], respectively) from 2020 to 2030 (**Supplementary Table 6**; **Supplementary Figures 5** and **7**). Despite having a lower baseline and smaller increase in the burden of breast cancer in women compared to other countries, China is expected to have a notably high annual percentage change in its ASIR for this burden (2.83 [95% CI: 1.29, 4.13]), ranking second among all 204 countries (**Supplementary Table 7**; **Supplementary Figures 5** and **7**). Conversely, Myanmar is projected to have the smallest annual percentage changes in ASIR, ASDR, and age-standardized DALY rates due to breast cancer in women from 2020 to 2030 (-12.06 [95% CI: -12.96, -10.89], -16.29 [95% CI: -18.79, -13.84], and -39.43 [95% CI: -41.42, -28.82]), followed by the Republic of Moldova and South Africa (**Supplementary Table 8**; **Supplementary Figures 5** and **7**).

The burden of breast cancer in women is expected to be highest in the Solomon Islands (ASIR: 6.43; ASDR: 5.12; age-standardized DALY rate: 5.96) per 100,000 (**Supplementary Table 9**; **Supplementary Figures 6** and **8**), followed by Lesotho (ASIR: 3.94; ASDR: 3.58; age-standardized DALY rate: 3.83) per 100,000 (**Supplementary Table 9**; **Supplementary Figures 6** and **8**). On the other hand, the lowest ASIR is projected to be in Myanmar (-2.27), followed by Kyrgyzstan (-1.57) and Iceland (-1.29) per 100,000 (**Supplementary Table 10**; **Supplementary Figures 6** and **8**).

### 3.4 Correlation of the burden of breast bancer in women worldwide in 1990–2019 with that in 2020–2030

A significant correlation was discovered between the burden of breast cancer in women from 1990 to 2019 and the projected burden for 2020 to 2030. Specifically, the analysis of EAPCs in key parameters across 204 countries during these time frames showed a positive correlation (ASIR: r = 0.38, p< 0.01; ASDR: r = 0.37, p< 0.01; and age-standardized DALY rate: r = 0.31, p< 0.01; see **Supplementary Figure 9**).

A significant positive correlation was observed between the ASIR of breast cancer in women globally in 1990 and the projected rates for 2030 (r = 0.62, p< 0.01; **Supplementary Figure 10**). This indicates that higher ASIRs in 1990 are associated with higher projected rates in 2030. Additionally, while the correlations between the ASDR and age-standardized DALY rates due to breast cancer in women globally in 1990 and their projected rates for 2030 were not as strong as those for ASIRs, they were still positive (r = 0.30, p< 0.01 and r = 0.25, p< 0.01, respectively; **Supplementary Figure 10**).

## 4 Discussion

In this study, the GBD 2019 data was analyzed to examine the temporal trends in the global burden of breast cancer from 1990 to 2019, as well as to forecast these trends from 2020 to 2030 on a global scale and by country. It was projected that the ASIR of breast cancer in women will increase annually worldwide from 1990 to 2030. However, from 2020 to 2030, the ASIR of breast cancer in women is expected to decrease in high-SDI regions and increase in other SDI regions (24).

The projected burden of breast cancer in women is expected to vary across regions of different SDI levels from 2020 to 2030. Particularly noteworthy is the anticipated decrease in this burden in high-SDI regions. This decrease in the ASIR of breast cancer in women in high-SDI regions is likely attributed to the more advanced medical technology, effective preventive measures, and greater health awareness among the population in these regions. These factors facilitate better screening and early detection of breast cancer. The Age-Standardized DALY rates and ASDRs due to breast cancer in women are projected to decline in high-SDI and middle-high-SDI regions, but increase in other-SDI regions, particularly low-SDI regions. The anticipated rise in the incidence of breast cancer among women in low-SDI regions could be impacted by geographical variables like climate and environmental exposure. For instance, specific pollutants and lifestyle choices prevalent in developing regions with low industrialization levels may increase the susceptibility to breast cancer. Additionally, in comparison to the focus on healthy living, stress reduction, self-examination, and medical screenings in higher SDI regions, occupational stress and lifestyle decisions in low-SDI regions might escalate the risk of breast cancer (23, 25). Variations in the impact of breast cancer in women are influenced by economic and social factors, underscoring the importance of strategic allocation of health resources and medical interventions to mitigate the burden of breast cancer. In countries with limited resources, priority should be placed on prevention, increased screening, early detection, and improved access to treatment to effectively decrease mortality and DALYs (26–28).

From 2020 to 2030, the burden of breast cancer among women of childbearing age is expected to rise across most age groups globally, with the highest increase projected in women aged 45–49 (29, 30). However, a decrease in incidence rates is anticipated for women aged 40–49 in regions with high SDI. This highlights that the impact of breast cancer varies depending on age and the region’s SDI. The rise in global breast cancer incidence is linked to societal aging (31), leading to greater exposure to genetic variations and environmental factors, ultimately increasing cancer rates. Consequently, the overall risk of cancer rises with age (32–34).

The burdens of breast cancer in women childbearing age are projected to vary by region from 2020 to 2030. The largest increase in the ASDR and age-standardized DALY rate due to breast cancer in women is projected to occur in Central sub-Saharan Africa (EAPCs of 1.62 and 1.52, respectively), followed by Oceania (EAPCs of 1.42 and 1.49, respectively). East Asia is expected to have the fastest-growing ASIR among all regions during this period. The projected increase in Central sub-Saharan Africa and Oceania may be attributed to low levels of economic development, unequal distribution of health resources, and weak healthcare systems (35, 36), while the rise in East Asia could be linked to changes in lifestyle (such as dietary habits and pace of life) and genetic factors (4, 37).The Global Breast Cancer Initiative (GBCI) is laudable for its emphasis on health promotion, early detection, prompt diagnosis, and comprehensive treatment of breast cancer. Emphasizing the importance of early detection and effective screening programs is essential in the worldwide battle against breast cancer, as it can significantly decrease the prevalence of this disease.

Projections on the burden of breast cancer in women across 204 countries from 2020 to 2030, and its growth trends, reveal significant disparities. The Solomon Islands are expected to experience the fastest growth in this burden, with the EAPC in its ASIR and ASDR of and DALY rates due to breast cancer in women projected to significantly surpass those of other countries. Additionally, China and Myanmar are noteworthy, with China having the second highest projected growth rate in the ASIR of breast cancer in women among the 204 countries, while Myanmar is expected to have the lowest burden. The anticipated rapid rise in the burden of breast cancer in women in the Solomon Islands from 2020 to 2030 may be attributed to its socioeconomic and health conditions, including limited medical resources and low public health awareness. Conversely, China’s high growth rate in the ASIR of breast cancer in women may be linked to rapid urbanization and lifestyle changes, whereas Myanmar’s low burden may be influenced by lifestyle and genetic factors, such as dietary habits and ethnic background, which warrant further exploration (38, 39).

Our study revealed a notable association between the burden of breast cancer in women from 1990 to 2019 and the projected burden from 2020 to 2030, suggesting a potential link between past and future trends. Furthermore, a strong positive correlation (r = 0.62) was observed between the ASIR of breast cancer in women in 1990 and the projected rate in 2030, indicating that regions with high burdens in 1990 are likely to continue facing high burdens in 2030. However, weaker positive correlations were found between the ASDRs of and DALY rates due to breast cancer in women (r = 0.30 and 0.25, respectively).

The positive correlation between historical and projected burdens of breast cancer in women may reflect the ongoing impact of the disease’s pathogenesis and its interaction with genetics, environmental, and lifestyle factors. The significant positive correlation between the ASIR of breast cancer in women in 1990 and its projected rate in 2030 could be attributed to the stability of long-term lifestyles (40), environmental factors (41), and socioeconomic conditions (42, 43). On the other hand, the weaker correlations between the ASDR and DALY rates due to breast cancer in women in 1990 and their projections for 2030 may indicate the influence of advancements in medical technology, treatment methods, and changes in public health strategies on mortality and overall burden of the disease. These factors may lead to variations in mortality and burden across regions, even when incidence rates remain relatively constant. Consequently, a global disparity in the burden of breast cancer in women may emerge, with some regions facing higher burdens due to limited health resources and preventive measures, while others experience lower burdens due to improved healthcare and preventive measures (18, 44).

These findings emphasize the significance of utilizing historical data to forecast future trends. They also emphasize the necessity for global strategies in preventing and treating breast cancer in women to take into account regional disparities and long-term patterns. Furthermore, they demonstrate the critical need for enhancing measures for breast cancer prevention and treatment, particularly in areas with a historical prevalence of the disease (45).

This comprehensive analysis highlights a decrease in the burden of breast cancer in women from 1990 to 2019, yet emphasizes the persistence of key risk factors and challenges that require attention (46). To address these issues, we recommend the following actions: Firstly, there is a necessity for the development and implementation of enhanced public-health strategies globally, particularly in low-SDI regions, to bolster breast cancer screening and prevention efforts. Secondly, there is a call for increased investment in medical resources for the early diagnosis and treatment of breast cancer, particularly in underserved areas. Additionally, there should be a focus on raising public health awareness and education to improve women’s knowledge of breast cancer, promoting regular check-ups for early detection. Lastly, it is crucial to encourage international researchers to explore the link between breast cancer and lifestyle, promote healthy behaviors like moderate exercise and a balanced diet, and foster global collaboration to share research findings and medical resources for more effective prevention and treatment of breast cancer in women worldwide (23, 25, 47).

This study utilized long-term data to comprehensively evaluate the global burden of breast cancer in women. It also investigated the impact of factors like SDIs, regional variations, and gender disparities on this burden, particularly focusing on the influence of SDI changes. These findings facilitated the development of strategic recommendations to mitigate the burden in countries and regions with diverse SDIs.

However, this study also has certain limitations. Firstly, despite utilizing a wide range of data, potential biases and inaccuracies within the data could have impacted the precision of our findings. The GBD estimates relied heavily on an algorithm that was significantly influenced by the quality and quantity of data retroactively used in the modeling process. Moreover, data scarcity, particularly in regions such as Latin America, sub-Saharan Africa, and Asia, could have posed challenges. Secondly, while our predictive model accounted for various stratification factorslike SDI region, age distribution, and country, it may have overlooked future events or emerging risk factors. Lastly, our strategic recommendations may necessitate additional validation and implementation to verify their efficacy and relevance.

## 5 Conclusion

This study examined the historical and projected trends in the global burden of breast cancer among women from 1990 to 2030. The analysis forecasts an overall increase in the ASIR of breast cancer in women worldwide, with the exception of high SDI regions where a decrease is projected. The ASIR is expected to rise with age, peaking among women aged 45–49. Sub-Saharan Africa and Oceania are anticipated to experience the most significant increases in breast cancer burden among women, with the Solomon Islands and China projected to have the fastest growth in ASIR. Additionally, a notable positive correlation exists between the ASIR of breast cancer in women in 1990 and the projected rates for 2030. In conclusion, efforts should be directed towards addressing the burden of breast cancer among women aged 45–49 in sub-Saharan Africa, Oceania, and specifically in the Solomon Islands and China.

## Data availability statement

The original contributions presented in the study are included in the article/**Supplementary Material**. Further inquiries can be directed to the corresponding author.

## Author contributions

SZ: Data curation, Methodology, Writing – original draft. ZJ: Data curation, Methodology, Writing – original draft. LB: Conceptualization, Project administration, Writing – review & editing. PS: Conceptualization, Project administration, Writing – review & editing.
