# Supplementary material for: The global burden of breast cancer in women from 1990 to 2030: assessment and projection based on the global burden of disease study 2019
Source: Front Oncol. 2024 Jun 20;14:1364397. doi: 10.3389/fonc.2024.1364397 (PMC11222408; doi:10.3389/fonc.2024.1364397)

**SFIGURE 1**

The trends of incidence rate for global burden of breast cancer in women in 2019, by SDI regions and age group. (A) Global (B) High SDI (C) High-middle SDI (D) Middle SDI (E) Low-middle SDI (F) Low SDI.


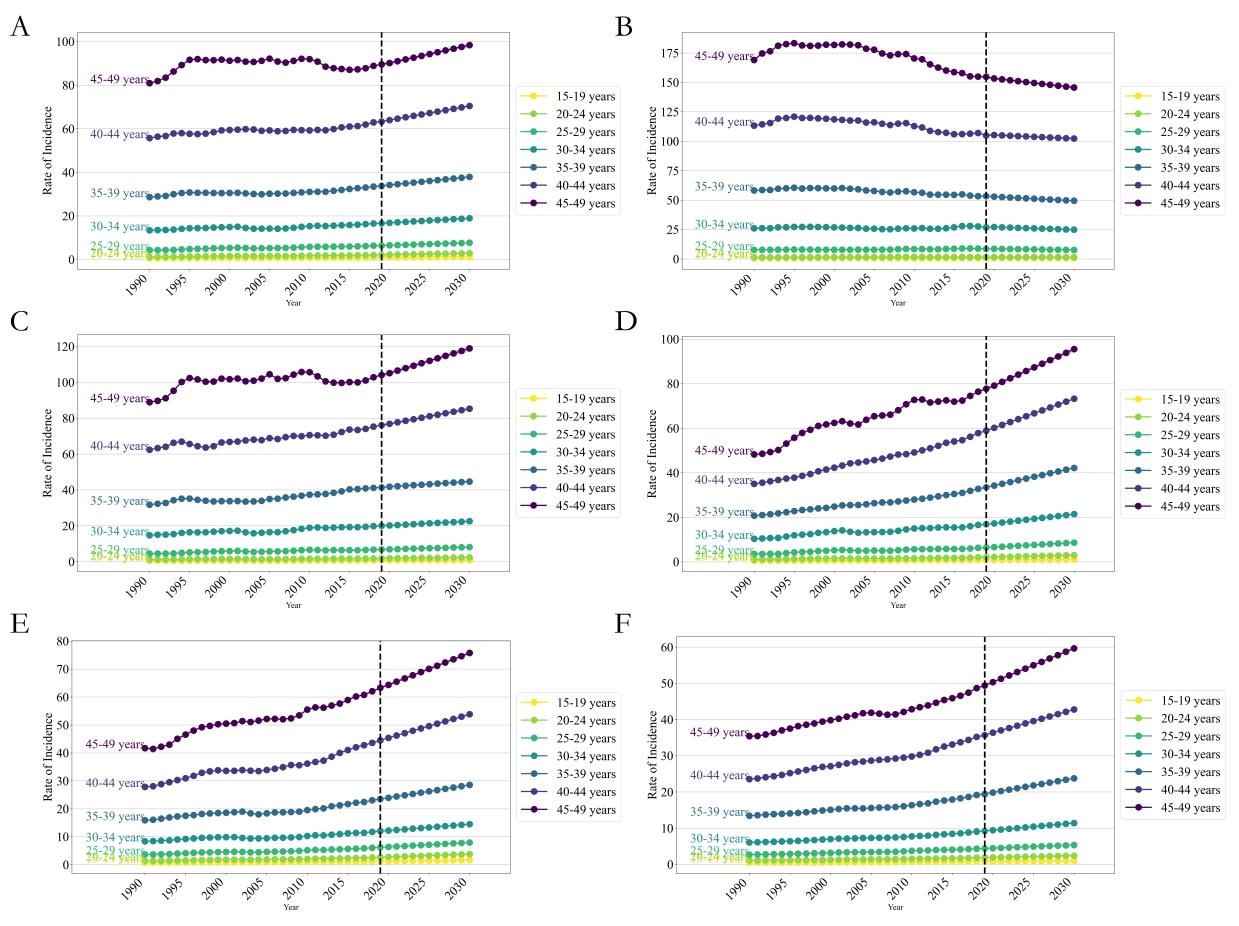


**SFIGURE 2**

The trends of death rate for global burden of breast cancer in women in 2019, by SDI regions and age group. (A) Global (B) High SDI (C) High-middle SDI (D) Middle SDI (E) Low-middle SDI (F) Low SDI.

**
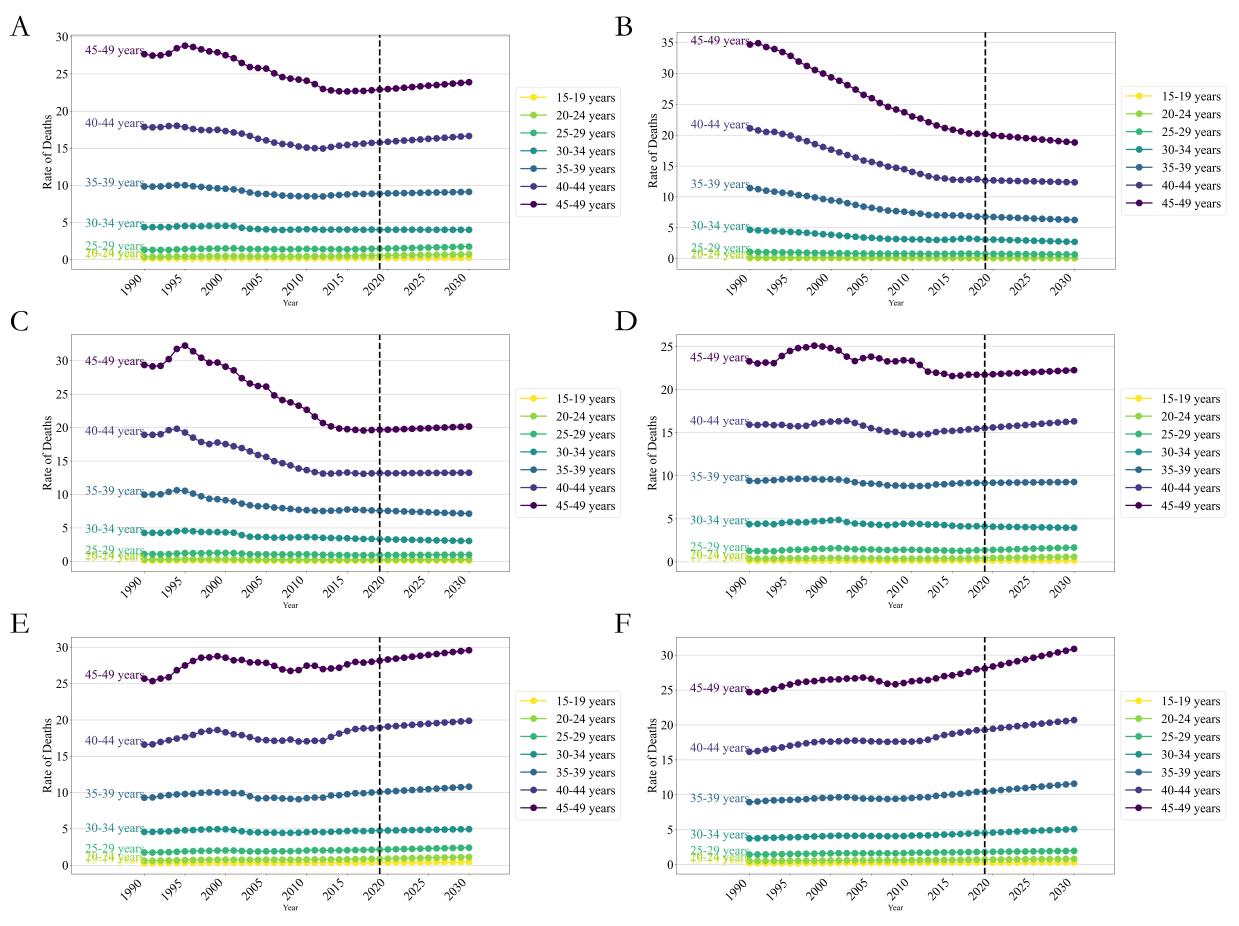
**

**SFIGURE 3**

The trends of DALY rate for global burden of breast cancer in women in 2019, by SDI regions and age group. (A) Global (B) High SDI (C) High-middle SDI (D) Middle SDI (E) Low-middle SDI (F) Low SDI.


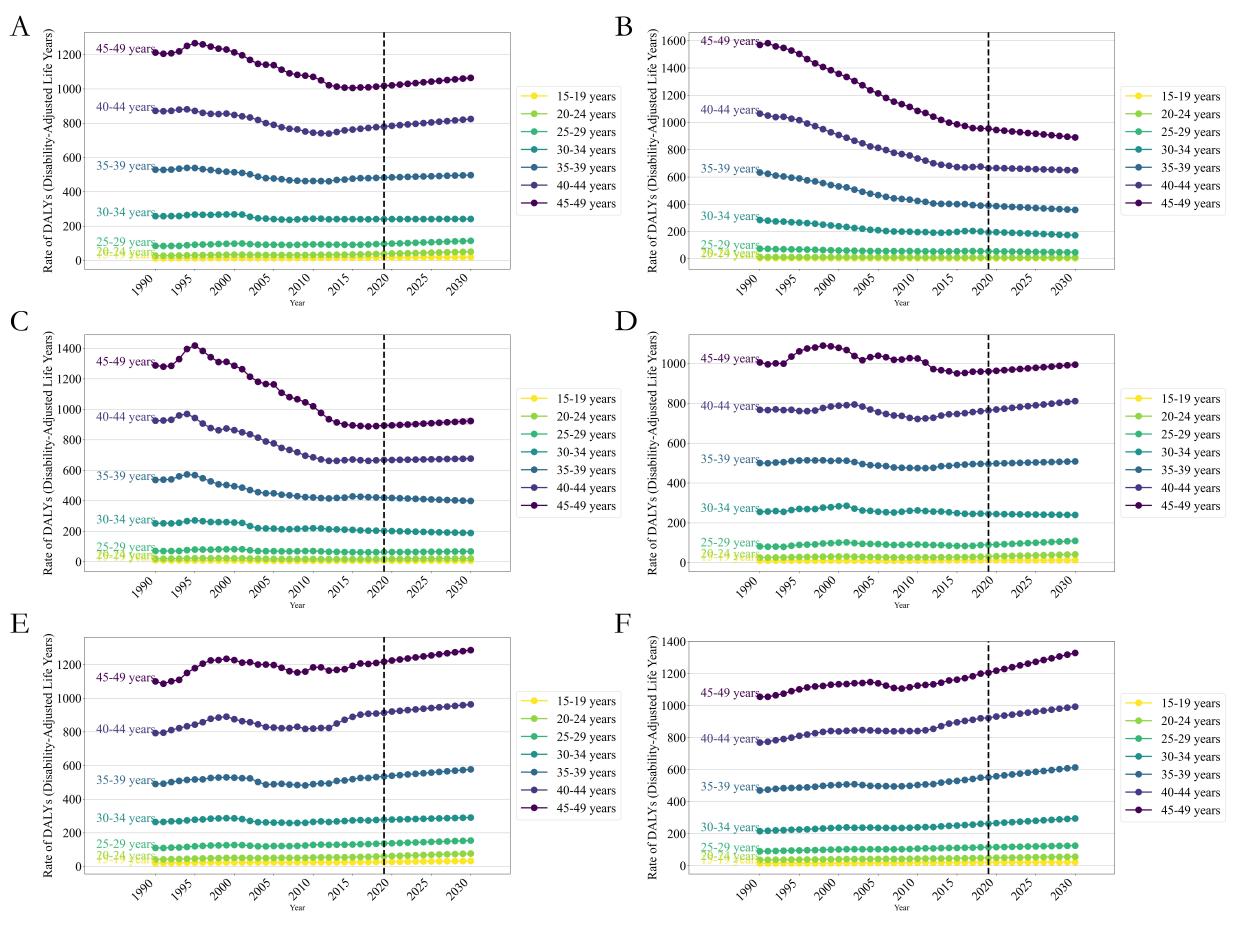


**SFIGURE 4**

Rate of global burden of breast cancer in women in 2030, by age groups. (A) Incidence rate(B) Death rate (C) age-standardized DALY rate.

**
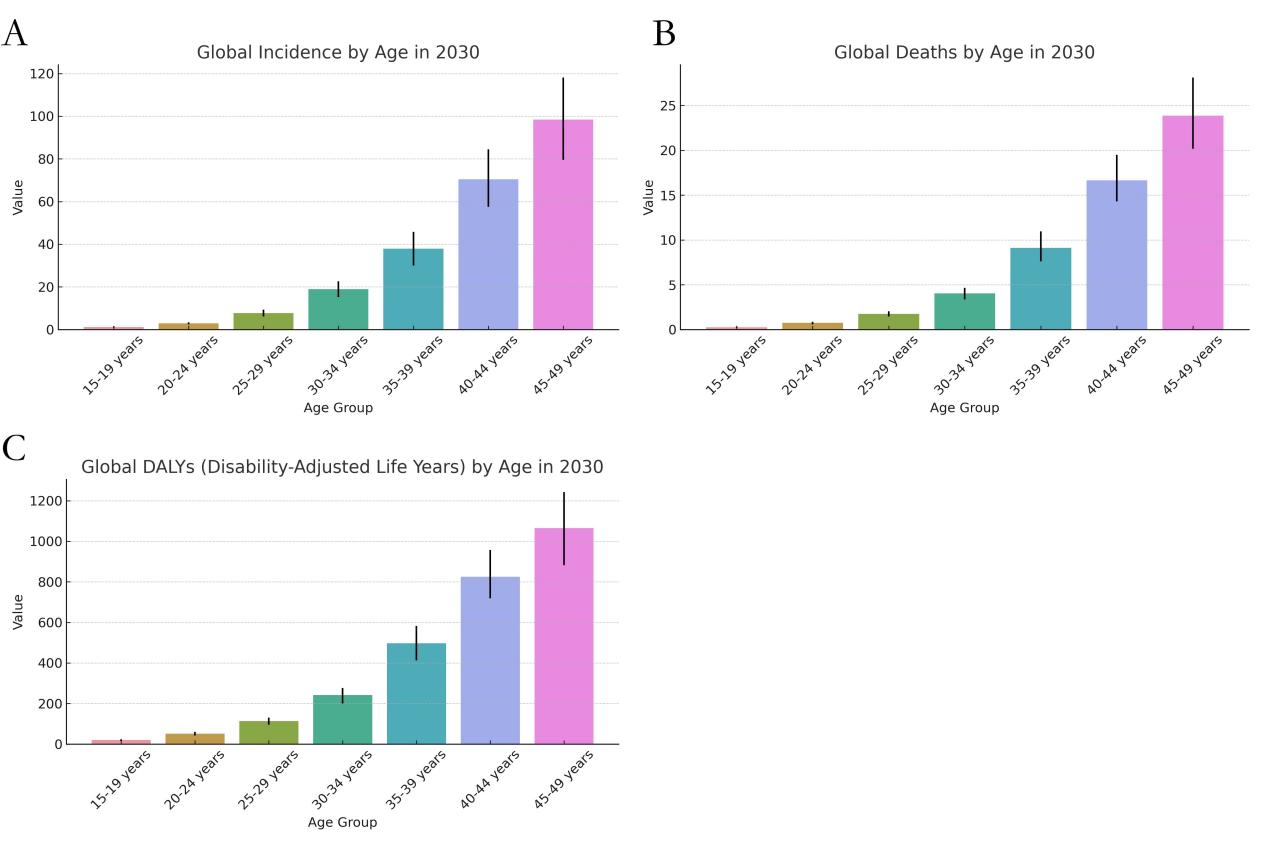
**

**SFIGURE 5**

The EAPC heat map of global burden of breast cancer in women in 204 countries from 2020 to 2030. (A) The EAPC of ASIR (B) The EAPC of ASDR (C) The EAPC of age-standardized DALY rate. EAPC = estimated annual percentage change. ASIR = age standardized incidence rate. ASDR = age standardized death rate.


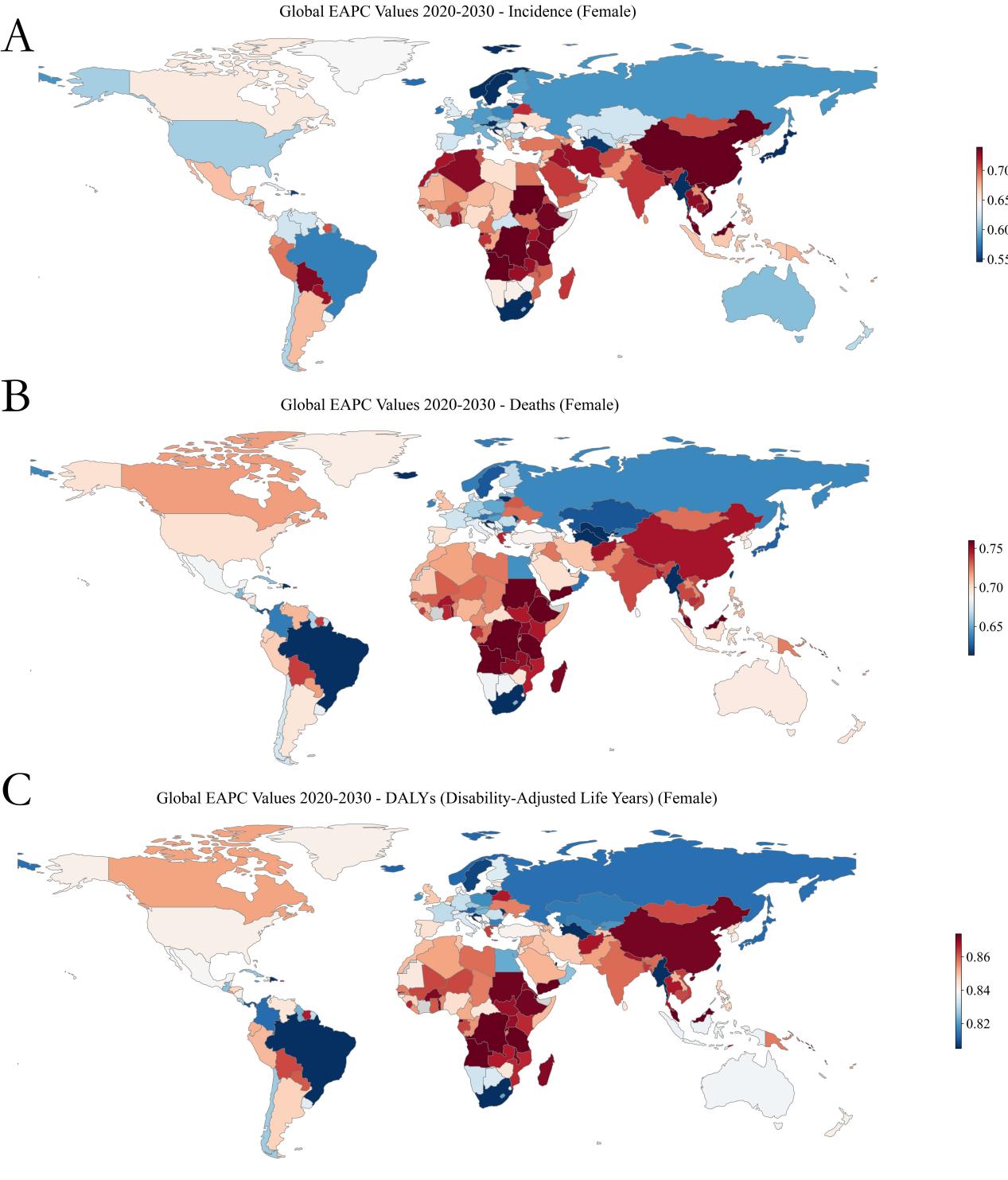


**SFIGURE 6**

The EAPC heat map of global burden of breast cancer in women in 204 countries from 1990 to 2019. (A) The EAPC of ASIR (B) The EAPC of ASDR (C) The EAPC of age-standardized DALY rate. EAPC = estimated annual percentage change. ASIR = age standardized incidence rate. ASDR = age standardized death rate.


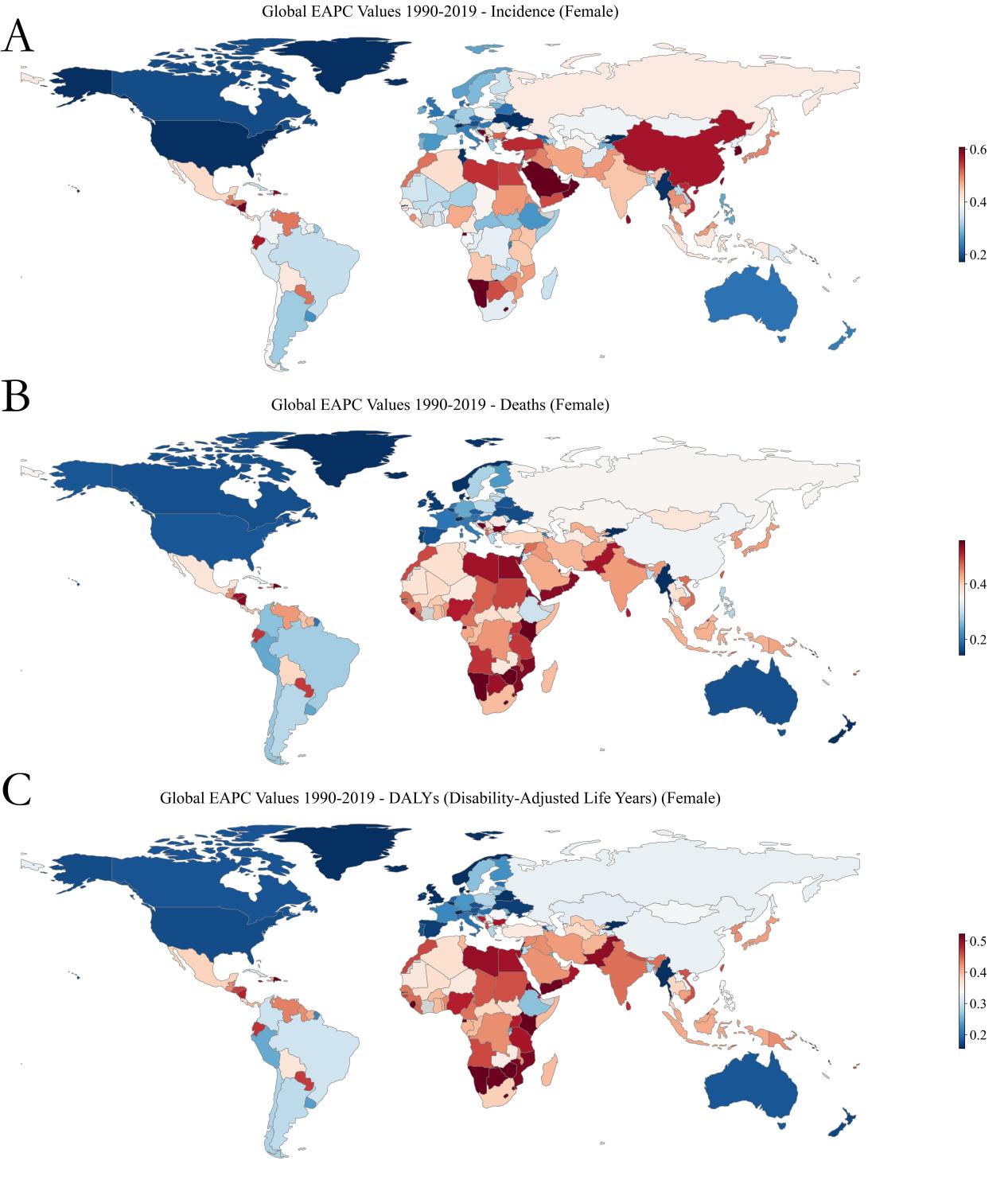


**SFIGURE 7**

The Age-standardized rates heat map of global burden of breast cancer in women in 204 countries in 2030 (A) ASIR (B) ASDR (C) age-standardized DALY rate. ASIR = age standardized incidence rate. ASDR = age standardized death rate.


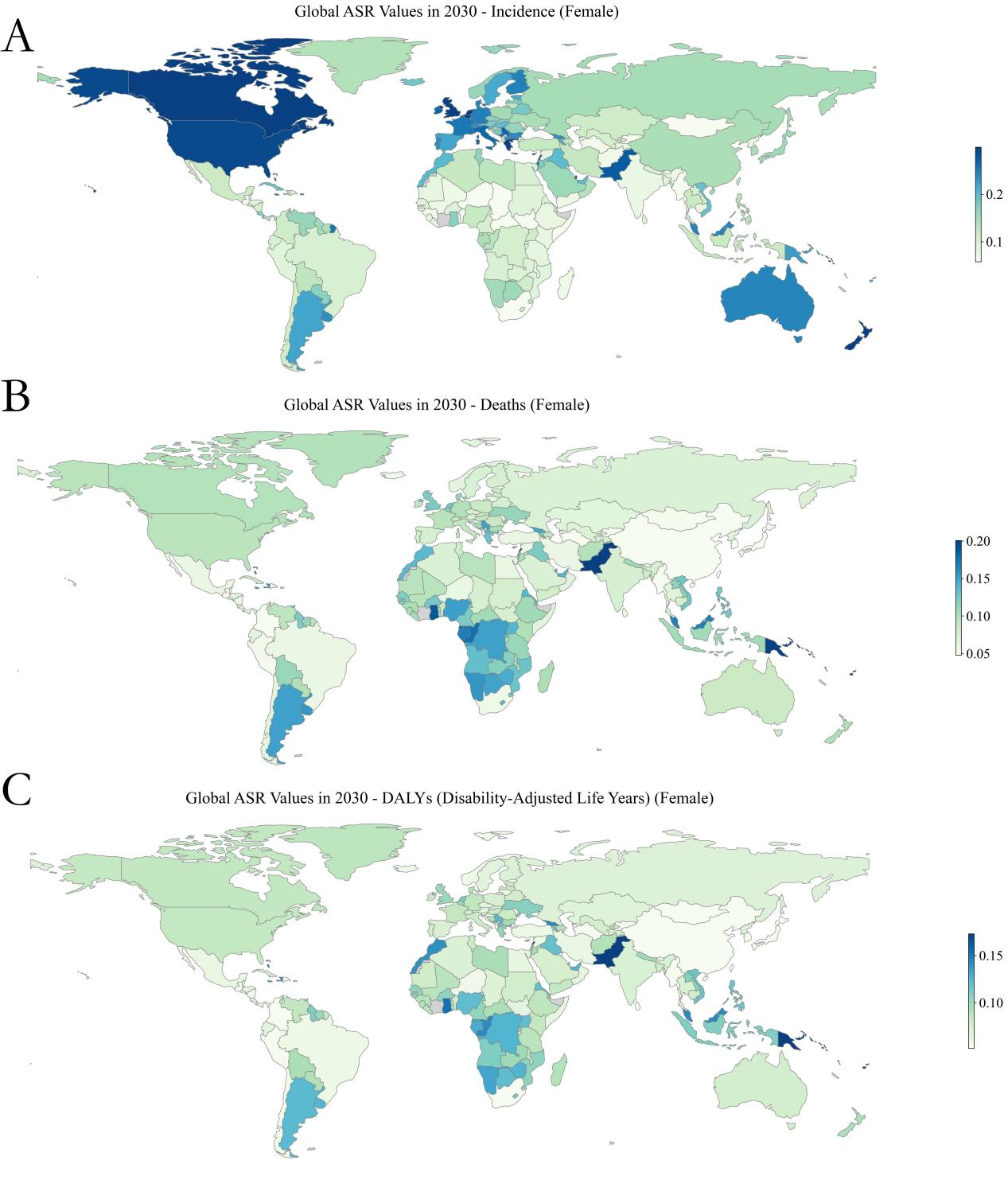


**SFIGURE 8**

The Age-standardized rates heat map of global burden of breast cancer in women in 204 countries in 2019 (A) ASIR (B) ASDR (C) age-standardized DALY rate. ASIR = age standardized incidence rate. ASDR = age standardized death rate.


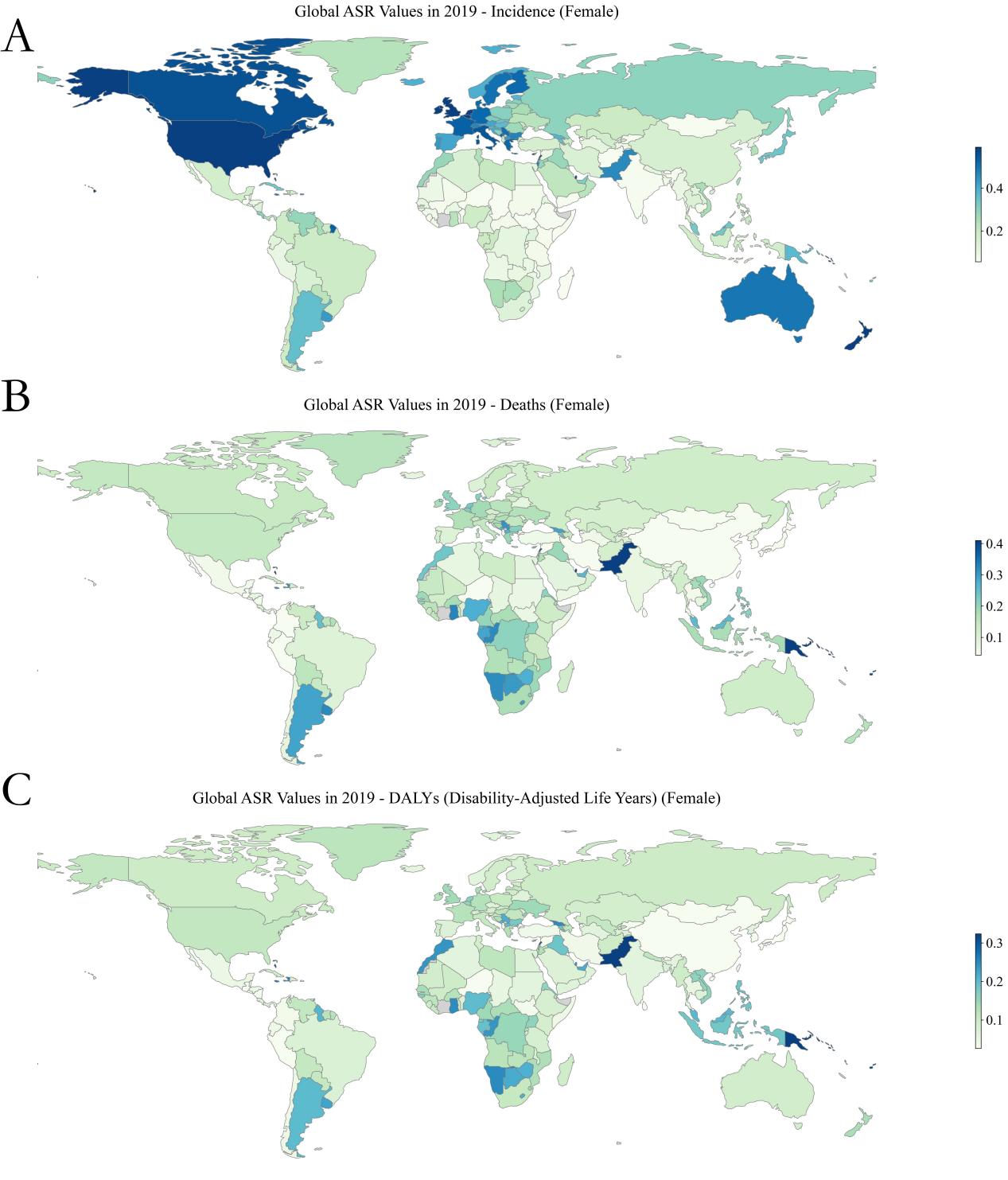


**SFIGURE 9**

The EAPC correlation between 1990-2019 and 2020-2030 of breast cancer global burden in women in different countries. (A) EAPC of ASIR (B) EAPC of ASDR (C) EAPC of age-standardized DALY rate. ASIR = age standardized incidence rate. ASDR = age standardized death rate. EAPC = estimated annual percentage change.


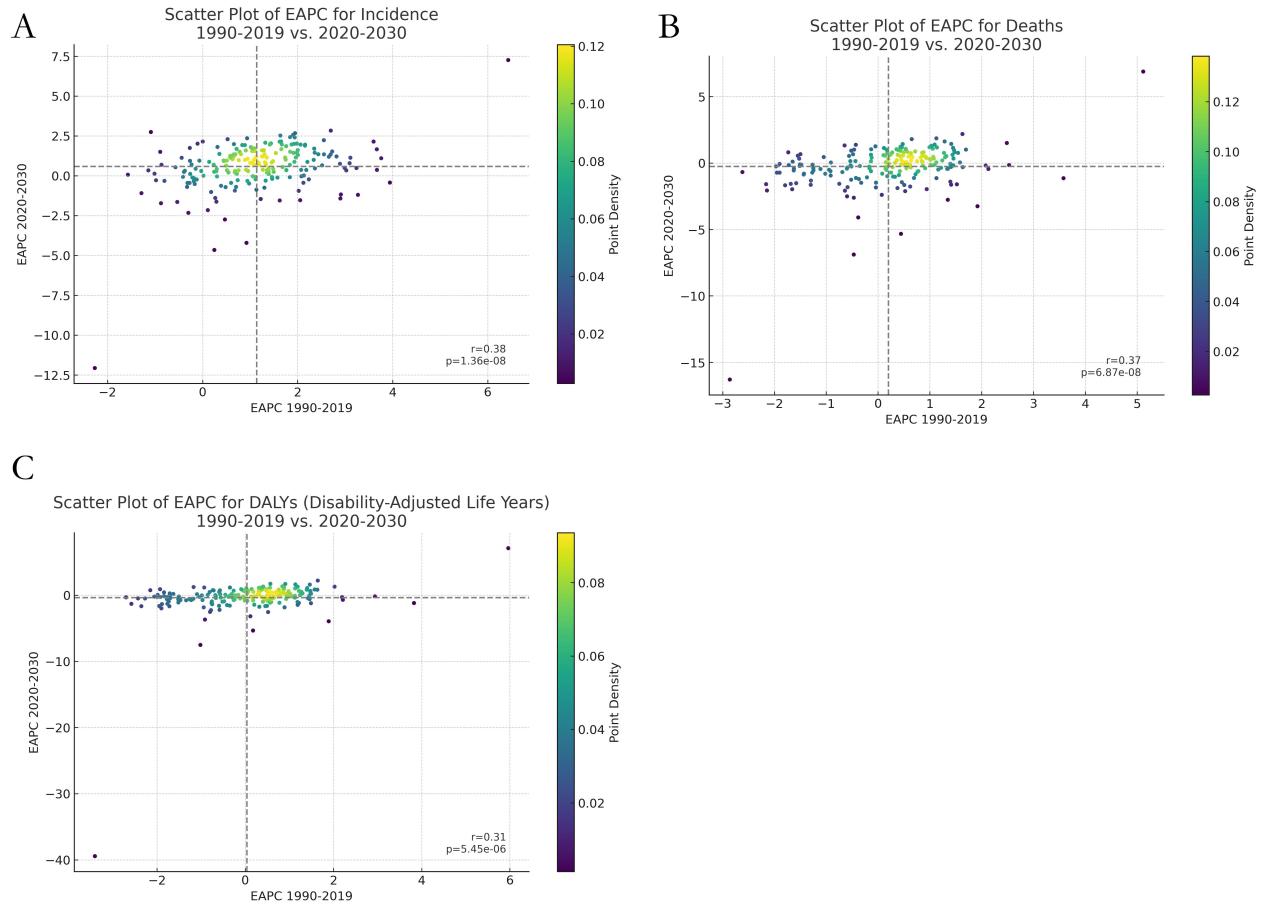


**SFIGURE 10**

The Age-standardized rates correlation between 1990 and 2030 of breast cancer global burden in women in different countries. (A)ASIR (B) ASDR (C)age-standardized DALY rate. ASIR = age standardized incidence rate. ASDR = age standardized death rate.


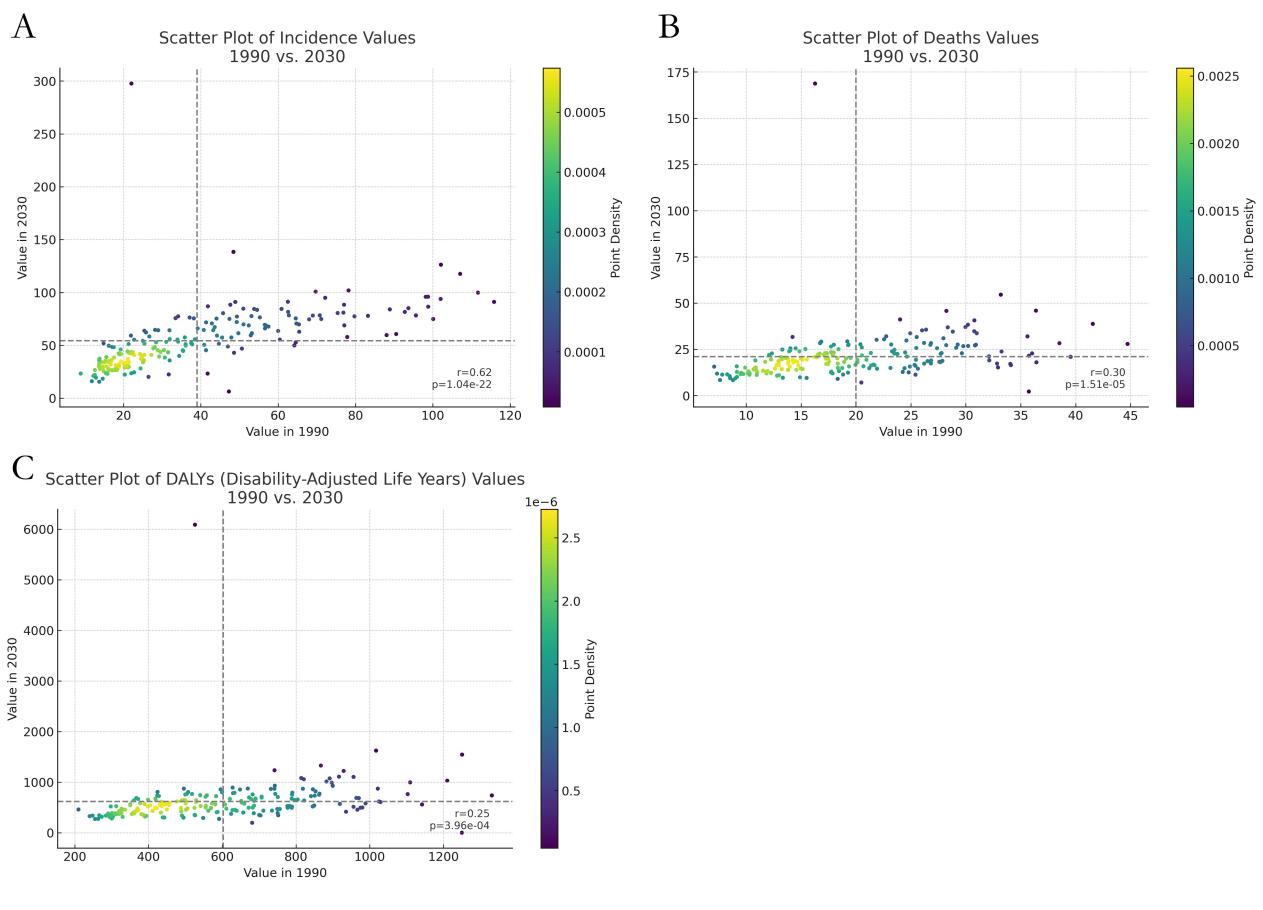

Supplement: Supplementary file 1 [file DataSheet_1.docx]
