# Supplementary material for: The global burden of breast cancer in women from 1990 to 2030: assessment and projection based on the global burden of disease study 2019
Source: Front Oncol. 2024 Jun 20;14:1364397. doi: 10.3389/fonc.2024.1364397 (PMC11222408; doi:10.3389/fonc.2024.1364397)
Supplement: Supplementary file 2 [file Table_1.docx]

**STABLE 1 The Incidence rate of global breast cancer burden in women from 1990 to 2030, by age groups.**

| **year** | **15-19 years** | **20-24 years** | **25-29 years** | **30-34 years** | **35-39 years** | **40-44 years** | **45-49 years** |
| --- | --- | --- | --- | --- | --- | --- | --- |
| **1990** | 0.52 (0.47, 0.59) | 1.23 (1.10, 1.34) | 4.36 (4.03, 4.68) | 13.37 (12.53, 14.15) | 28.66 (27.09, 30.35) | 55.72 (53.45, 58.39) | 80.94 (77.72, 84.44) |
| **1991** | 0.54 (0.48, 0.61) | 1.25 (1.13, 1.37) | 4.35 (4.04, 4.67) | 13.54 (12.77, 14.33) | 28.95 (27.57, 30.52) | 56.40 (54.33, 58.86) | 81.87 (78.82, 85.18) |
| **1992** | 0.55 (0.49, 0.62) | 1.28 (1.15, 1.40) | 4.37 (4.04, 4.69) | 13.59 (12.78, 14.35) | 29.22 (27.76, 30.75) | 56.70 (54.62, 59.29) | 83.48 (80.74, 86.67) |
| **1993** | 0.57 (0.51, 0.64) | 1.31 (1.19, 1.43) | 4.42 (4.09, 4.74) | 13.74 (13.00, 14.49) | 30.01 (28.68, 31.55) | 57.83 (55.60, 60.33) | 86.26 (83.21, 89.36) |
| **1994** | 0.58 (0.52, 0.65) | 1.37 (1.25, 1.48) | 4.63 (4.30, 4.96) | 14.09 (13.29, 14.86) | 30.49 (29.11, 31.90) | 58.01 (55.90, 60.29) | 89.26 (86.40, 92.36) |
| **1995** | 0.59 (0.53, 0.66) | 1.43 (1.30, 1.55) | 4.84 (4.47, 5.23) | 14.36 (13.44, 15.23) | 30.78 (29.48, 32.18) | 57.71 (55.68, 60.13) | 91.65 (88.72, 94.65) |
| **1996** | 0.60 (0.54, 0.67) | 1.47 (1.33, 1.59) | 4.94 (4.54, 5.32) | 14.39 (13.42, 15.31) | 30.64 (29.36, 32.00) | 57.56 (55.47, 59.75) | 91.95 (88.92, 95.09) |
| **1997** | 0.61 (0.54, 0.69) | 1.50 (1.37, 1.63) | 5.02 (4.61, 5.41) | 14.48 (13.47, 15.37) | 30.66 (29.41, 31.99) | 57.79 (55.66, 59.91) | 91.53 (88.53, 94.95) |
| **1998** | 0.61 (0.55, 0.68) | 1.54 (1.39, 1.68) | 5.18 (4.68, 5.61) | 14.69 (13.54, 15.66) | 30.55 (29.24, 31.94) | 58.43 (56.24, 60.74) | 91.45 (88.17, 95.00) |
| **1999** | 0.62 (0.56, 0.69) | 1.58 (1.42, 1.73) | 5.28 (4.75, 5.73) | 14.80 (13.58, 15.87) | 30.51 (29.16, 31.86) | 59.26 (57.12, 61.44) | 91.74 (88.24, 95.18) |
| **2000** | 0.63 (0.56, 0.70) | 1.61 (1.45, 1.74) | 5.35 (4.84, 5.77) | 14.92 (13.72, 16.00) | 30.52 (29.02, 32.01) | 59.43 (57.48, 61.48) | 91.24 (87.64, 94.89) |
| **2001** | 0.63 (0.57, 0.70) | 1.62 (1.49, 1.75) | 5.45 (4.99, 5.85) | 15.06 (13.99, 16.08) | 30.62 (29.11, 32.11) | 59.62 (57.44, 61.68) | 91.57 (88.18, 95.03) |
| **2002** | 0.63 (0.57, 0.70) | 1.61 (1.48, 1.72) | 5.28 (4.88, 5.64) | 14.50 (13.64, 15.30) | 30.38 (28.76, 31.95) | 59.84 (58.00, 61.82) | 90.84 (87.49, 94.18) |
| **2003** | 0.64 (0.58, 0.71) | 1.59 (1.47, 1.70) | 5.15 (4.85, 5.47) | 14.16 (13.43, 14.90) | 30.06 (28.64, 31.46) | 59.71 (57.77, 61.75) | 90.66 (87.75, 93.46) |
| **2004** | 0.64 (0.58, 0.71) | 1.59 (1.49, 1.71) | 5.18 (4.90, 5.45) | 14.17 (13.55, 14.88) | 29.92 (28.65, 31.23) | 59.10 (57.08, 61.22) | 91.21 (88.23, 94.38) |
| **2005** | 0.65 (0.59, 0.72) | 1.62 (1.50, 1.73) | 5.26 (4.98, 5.56) | 14.22 (13.61, 14.87) | 30.24 (28.98, 31.56) | 59.28 (57.32, 61.41) | 92.18 (89.26, 95.25) |
| **2006** | 0.65 (0.59, 0.73) | 1.62 (1.51, 1.74) | 5.27 (5.02, 5.56) | 14.14 (13.52, 14.79) | 30.27 (29.02, 31.56) | 58.86 (56.80, 60.94) | 90.86 (87.83, 93.81) |
| **2007** | 0.66 (0.60, 0.74) | 1.64 (1.52, 1.76) | 5.32 (5.03, 5.60) | 14.24 (13.65, 14.88) | 30.32 (29.10, 31.65) | 58.99 (56.65, 61.35) | 90.36 (87.33, 93.42) |
| **2008** | 0.69 (0.62, 0.77) | 1.67 (1.56, 1.79) | 5.49 (5.20, 5.77) | 14.60 (13.95, 15.32) | 30.59 (29.38, 31.95) | 59.51 (57.22, 62.04) | 91.19 (88.00, 94.70) |
| **2009** | 0.71 (0.64, 0.79) | 1.71 (1.60, 1.84) | 5.70 (5.40, 6.03) | 15.01 (14.31, 15.75) | 30.84 (29.56, 32.21) | 59.36 (57.01, 61.73) | 92.10 (88.79, 95.42) |
| **2010** | 0.73 (0.65, 0.82) | 1.77 (1.64, 1.90) | 5.84 (5.48, 6.20) | 15.33 (14.65, 16.13) | 31.04 (29.73, 32.40) | 59.29 (56.93, 61.88) | 91.93 (88.08, 95.86) |
| **2011** | 0.75 (0.67, 0.84) | 1.81 (1.67, 1.96) | 5.87 (5.51, 6.26) | 15.51 (14.71, 16.38) | 31.12 (29.77, 32.55) | 59.49 (56.90, 62.24) | 90.84 (86.96, 95.18) |
| **2012** | 0.76 (0.68, 0.86) | 1.82 (1.67, 1.98) | 5.83 (5.46, 6.23) | 15.39 (14.66, 16.18) | 31.08 (29.71, 32.57) | 59.32 (56.76, 62.16) | 88.52 (85.00, 92.75) |
| **2013** | 0.79 (0.70, 0.89) | 1.85 (1.69, 2.01) | 5.92 (5.51, 6.33) | 15.56 (14.78, 16.44) | 31.55 (30.06, 33.12) | 59.85 (57.27, 62.90) | 87.82 (84.07, 91.85) |
| **2014** | 0.81 (0.71, 0.92) | 1.87 (1.72, 2.06) | 5.96 (5.57, 6.40) | 15.72 (14.85, 16.67) | 31.93 (30.46, 33.69) | 60.68 (57.73, 64.08) | 87.43 (83.41, 92.01) |
| **2015** | 0.83 (0.72, 0.95) | 1.91 (1.73, 2.12) | 5.99 (5.57, 6.42) | 15.85 (14.93, 16.89) | 32.41 (30.75, 34.19) | 61.05 (57.96, 64.47) | 87.08 (82.66, 91.58) |
| **2016** | 0.84 (0.73, 0.97) | 1.95 (1.76, 2.16) | 6.05 (5.59, 6.56) | 16.01 (14.96, 17.10) | 32.75 (30.97, 34.70) | 61.25 (57.80, 65.10) | 87.25 (82.63, 92.11) |
| **2017** | 0.86 (0.75, 1.01) | 2.00 (1.78, 2.25) | 6.17 (5.60, 6.75) | 16.24 (14.97, 17.62) | 33.03 (30.68, 35.51) | 61.96 (57.69, 66.72) | 87.76 (81.77, 94.17) |
| **2018** | 0.89 (0.77, 1.05) | 2.10 (1.85, 2.36) | 6.34 (5.70, 7.01) | 16.53 (15.05, 18.03) | 33.47 (30.64, 36.36) | 62.94 (57.76, 68.23) | 88.86 (81.72, 95.97) |
| **2019** | 0.91 (0.78, 1.08) | 2.16 (1.89, 2.44) | 6.41 (5.70, 7.15) | 16.63 (14.99, 18.33) | 33.80 (30.64, 37.12) | 63.29 (57.76, 69.46) | 89.56 (81.70, 97.90) |
| **2020** | 0.93 (0.80, 1.11) | 2.22 (1.93, 2.53) | 6.52 (5.75, 7.34) | 16.86 (15.03, 18.73) | 34.19 (30.62, 37.87) | 63.99 (57.74, 70.75) | 90.18 (81.34, 99.36) |
| **2021** | 0.96 (0.81, 1.14) | 2.29 (1.98, 2.62) | 6.64 (5.79, 7.53) | 17.07 (15.05, 19.12) | 34.56 (30.56, 38.67) | 64.64 (57.71, 72.12) | 91.00 (81.16, 101.24) |
| **2022** | 0.98 (0.83, 1.18) | 2.36 (2.02, 2.71) | 6.75 (5.83, 7.73) | 17.27 (15.06, 19.51) | 34.93 (30.50, 39.46) | 65.29 (57.68, 73.50) | 91.82 (80.98, 103.11) |
| **2023** | 1.00 (0.85, 1.21) | 2.43 (2.07, 2.80) | 6.87 (5.87, 7.92) | 17.48 (15.07, 19.91) | 35.30 (30.44, 40.26) | 65.93 (57.65, 74.87) | 92.65 (80.80, 104.99) |
| **2024** | 1.03 (0.87, 1.24) | 2.49 (2.11, 2.89) | 6.99 (5.91, 8.12) | 17.69 (15.09, 20.30) | 35.67 (30.38, 41.05) | 66.58 (57.62, 76.25) | 93.47 (80.62, 106.86) |
| **2025** | 1.05 (0.88, 1.28) | 2.56 (2.16, 2.99) | 7.10 (5.95, 8.32) | 17.89 (15.10, 20.69) | 36.04 (30.32, 41.85) | 67.23 (57.59, 77.62) | 94.29 (80.44, 108.73) |
| **2026** | 1.07 (0.90, 1.31) | 2.63 (2.21, 3.08) | 7.22 (5.99, 8.51) | 18.10 (15.11, 21.08) | 36.41 (30.25, 42.64) | 67.87 (57.56, 79.00) | 95.12 (80.26, 110.61) |
| **2027** | 1.10 (0.92, 1.35) | 2.70 (2.25, 3.17) | 7.33 (6.03, 8.71) | 18.31 (15.12, 21.47) | 36.78 (30.19, 43.44) | 68.52 (57.54, 80.37) | 95.94 (80.09, 112.48) |
| **2028** | 1.12 (0.93, 1.38) | 2.77 (2.30, 3.26) | 7.45 (6.07, 8.90) | 18.51 (15.14, 21.87) | 37.14 (30.13, 44.24) | 69.17 (57.51, 81.75) | 96.76 (79.91, 114.35) |
| **2029** | 1.14 (0.95, 1.41) | 2.84 (2.34, 3.35) | 7.57 (6.11, 9.10) | 18.72 (15.15, 22.26) | 37.51 (30.07, 45.03) | 69.82 (57.48, 83.12) | 97.59 (79.73, 116.23) |
| **2030** | 1.17 (0.97, 1.45) | 2.91 (2.39, 3.44) | 7.68 (6.15, 9.29) | 18.93 (15.16, 22.65) | 37.88 (30.01, 45.83) | 70.46 (57.45, 84.50) | 98.41 (79.55, 118.10) |

**STABLE 2 The Deaths rate of global breast cancer burden in women from 1990 to 2030, by age groups.**

| **year** | **15-19 years** | **20-24 years** | **25-29 years** | **30-34 years** | **35-39 years** | **40-44 years** | **45-49 years** |
| --- | --- | --- | --- | --- | --- | --- | --- |
| **1990** | 0.16 (0.14, 0.18) | 0.40 (0.35, 0.44) | 1.32 (1.19, 1.45) | 4.38 (4.00, 4.76) | 9.86 (9.19, 10.57) | 17.87 (17.01, 18.90) | 27.67 (26.30, 29.41) |
| **1991** | 0.16 (0.14, 0.19) | 0.40 (0.35, 0.45) | 1.31 (1.18, 1.43) | 4.39 (4.04, 4.72) | 9.84 (9.18, 10.55) | 17.81 (16.88, 18.85) | 27.48 (26.10, 29.12) |
| **1992** | 0.17 (0.14, 0.20) | 0.41 (0.36, 0.46) | 1.32 (1.17, 1.44) | 4.39 (4.02, 4.71) | 9.86 (9.18, 10.50) | 17.84 (16.98, 18.84) | 27.51 (26.31, 29.01) |
| **1993** | 0.17 (0.15, 0.20) | 0.42 (0.37, 0.47) | 1.32 (1.19, 1.45) | 4.39 (4.06, 4.72) | 9.96 (9.33, 10.68) | 17.99 (17.22, 18.94) | 27.74 (26.57, 29.07) |
| **1994** | 0.17 (0.15, 0.20) | 0.43 (0.38, 0.48) | 1.38 (1.24, 1.51) | 4.49 (4.15, 4.82) | 10.04 (9.44, 10.67) | 18.03 (17.21, 18.92) | 28.46 (27.34, 29.76) |
| **1995** | 0.18 (0.15, 0.21) | 0.45 (0.39, 0.50) | 1.43 (1.28, 1.57) | 4.54 (4.15, 4.91) | 10.02 (9.42, 10.65) | 17.83 (16.99, 18.77) | 28.80 (27.56, 30.17) |
| **1996** | 0.18 (0.16, 0.21) | 0.46 (0.41, 0.50) | 1.45 (1.30, 1.58) | 4.52 (4.12, 4.88) | 9.89 (9.31, 10.49) | 17.60 (16.79, 18.47) | 28.63 (27.34, 29.97) |
| **1997** | 0.18 (0.16, 0.21) | 0.46 (0.41, 0.51) | 1.46 (1.31, 1.60) | 4.51 (4.10, 4.87) | 9.79 (9.19, 10.40) | 17.46 (16.69, 18.31) | 28.31 (27.09, 29.67) |
| **1998** | 0.18 (0.16, 0.21) | 0.47 (0.42, 0.53) | 1.49 (1.32, 1.65) | 4.54 (4.08, 4.93) | 9.68 (9.07, 10.27) | 17.43 (16.55, 18.28) | 28.03 (26.72, 29.47) |
| **1999** | 0.18 (0.16, 0.21) | 0.48 (0.42, 0.53) | 1.51 (1.33, 1.67) | 4.54 (4.08, 4.96) | 9.61 (8.98, 10.19) | 17.48 (16.64, 18.33) | 27.91 (26.54, 29.20) |
| **2000** | 0.18 (0.16, 0.21) | 0.48 (0.43, 0.53) | 1.52 (1.35, 1.68) | 4.55 (4.07, 4.95) | 9.55 (8.93, 10.14) | 17.31 (16.53, 18.08) | 27.52 (26.21, 28.84) |
| **2001** | 0.18 (0.16, 0.21) | 0.48 (0.43, 0.53) | 1.54 (1.37, 1.69) | 4.53 (4.12, 4.91) | 9.47 (8.80, 10.11) | 17.13 (16.38, 17.91) | 27.12 (25.88, 28.35) |
| **2002** | 0.18 (0.16, 0.20) | 0.48 (0.43, 0.52) | 1.48 (1.34, 1.62) | 4.31 (3.97, 4.64) | 9.31 (8.62, 9.95) | 16.97 (16.24, 17.75) | 26.48 (25.26, 27.72) |
| **2003** | 0.18 (0.16, 0.21) | 0.46 (0.42, 0.51) | 1.43 (1.32, 1.54) | 4.16 (3.86, 4.45) | 9.05 (8.49, 9.62) | 16.66 (15.92, 17.44) | 25.92 (24.89, 27.06) |
| **2004** | 0.18 (0.16, 0.20) | 0.46 (0.42, 0.51) | 1.42 (1.32, 1.53) | 4.12 (3.85, 4.40) | 8.88 (8.40, 9.36) | 16.28 (15.57, 17.00) | 25.80 (24.73, 26.85) |
| **2005** | 0.18 (0.16, 0.20) | 0.46 (0.42, 0.50) | 1.43 (1.33, 1.53) | 4.08 (3.85, 4.33) | 8.84 (8.35, 9.31) | 16.06 (15.31, 16.77) | 25.71 (24.66, 26.74) |
| **2006** | 0.18 (0.16, 0.20) | 0.46 (0.42, 0.50) | 1.41 (1.31, 1.52) | 4.03 (3.79, 4.28) | 8.76 (8.29, 9.24) | 15.76 (15.05, 16.48) | 25.09 (24.13, 26.08) |
| **2007** | 0.18 (0.16, 0.20) | 0.45 (0.42, 0.50) | 1.40 (1.30, 1.51) | 4.00 (3.76, 4.25) | 8.64 (8.18, 9.10) | 15.57 (14.84, 16.35) | 24.59 (23.53, 25.65) |
| **2008** | 0.18 (0.16, 0.21) | 0.45 (0.42, 0.49) | 1.41 (1.31, 1.52) | 4.04 (3.81, 4.28) | 8.59 (8.17, 9.06) | 15.49 (14.76, 16.26) | 24.37 (23.37, 25.38) |
| **2009** | 0.19 (0.16, 0.22) | 0.46 (0.42, 0.50) | 1.43 (1.32, 1.55) | 4.07 (3.82, 4.34) | 8.54 (8.07, 9.06) | 15.23 (14.50, 16.05) | 24.25 (23.18, 25.41) |
| **2010** | 0.19 (0.17, 0.22) | 0.46 (0.42, 0.51) | 1.45 (1.34, 1.57) | 4.10 (3.87, 4.39) | 8.55 (8.10, 9.03) | 15.08 (14.39, 15.91) | 24.09 (23.00, 25.28) |
| **2011** | 0.20 (0.17, 0.23) | 0.47 (0.42, 0.52) | 1.45 (1.33, 1.57) | 4.10 (3.84, 4.37) | 8.54 (8.06, 9.04) | 15.02 (14.23, 15.79) | 23.64 (22.55, 24.90) |
| **2012** | 0.20 (0.17, 0.23) | 0.47 (0.42, 0.52) | 1.42 (1.31, 1.54) | 4.03 (3.78, 4.31) | 8.51 (8.07, 9.02) | 14.96 (14.20, 15.74) | 22.98 (21.87, 24.24) |
| **2013** | 0.20 (0.18, 0.24) | 0.48 (0.43, 0.54) | 1.43 (1.30, 1.56) | 4.06 (3.78, 4.34) | 8.66 (8.15, 9.21) | 15.16 (14.29, 16.05) | 22.79 (21.65, 23.98) |
| **2014** | 0.21 (0.18, 0.25) | 0.49 (0.43, 0.55) | 1.41 (1.29, 1.54) | 4.05 (3.78, 4.37) | 8.70 (8.15, 9.28) | 15.35 (14.36, 16.37) | 22.67 (21.44, 23.98) |
| **2015** | 0.21 (0.18, 0.25) | 0.50 (0.44, 0.56) | 1.41 (1.28, 1.56) | 4.05 (3.75, 4.36) | 8.80 (8.23, 9.42) | 15.45 (14.44, 16.57) | 22.64 (21.37, 24.06) |
| **2016** | 0.21 (0.18, 0.26) | 0.51 (0.45, 0.58) | 1.42 (1.28, 1.58) | 4.05 (3.72, 4.39) | 8.85 (8.24, 9.51) | 15.55 (14.46, 16.71) | 22.71 (21.22, 24.29) |
| **2017** | 0.22 (0.18, 0.26) | 0.52 (0.45, 0.60) | 1.44 (1.29, 1.60) | 4.03 (3.70, 4.40) | 8.85 (8.18, 9.55) | 15.63 (14.36, 16.88) | 22.71 (21.09, 24.33) |
| **2018** | 0.22 (0.19, 0.27) | 0.54 (0.47, 0.63) | 1.47 (1.32, 1.65) | 4.05 (3.67, 4.44) | 8.88 (8.10, 9.69) | 15.73 (14.40, 17.20) | 22.80 (21.01, 24.81) |
| **2019** | 0.22 (0.18, 0.27) | 0.56 (0.47, 0.64) | 1.49 (1.32, 1.68) | 4.04 (3.65, 4.43) | 8.89 (8.09, 9.80) | 15.77 (14.39, 17.29) | 22.89 (20.96, 24.98) |
| **2020** | 0.23 (0.19, 0.28) | 0.57 (0.49, 0.66) | 1.51 (1.33, 1.71) | 4.04 (3.62, 4.46) | 8.93 (8.06, 9.90) | 15.88 (14.39, 17.53) | 22.96 (20.87, 25.25) |
| **2021** | 0.23 (0.19, 0.28) | 0.59 (0.50, 0.69) | 1.54 (1.34, 1.74) | 4.04 (3.59, 4.48) | 8.95 (8.01, 10.01) | 15.95 (14.38, 17.72) | 23.05 (20.80, 25.54) |
| **2022** | 0.24 (0.19, 0.29) | 0.61 (0.51, 0.71) | 1.56 (1.36, 1.78) | 4.04 (3.57, 4.50) | 8.97 (7.97, 10.11) | 16.03 (14.37, 17.92) | 23.14 (20.73, 25.83) |
| **2023** | 0.24 (0.19, 0.29) | 0.62 (0.52, 0.73) | 1.59 (1.37, 1.81) | 4.04 (3.54, 4.52) | 8.99 (7.93, 10.22) | 16.11 (14.36, 18.12) | 23.24 (20.66, 26.11) |
| **2024** | 0.24 (0.19, 0.29) | 0.64 (0.53, 0.75) | 1.61 (1.38, 1.84) | 4.04 (3.52, 4.54) | 9.01 (7.89, 10.32) | 16.18 (14.35, 18.31) | 23.33 (20.59, 26.40) |
| **2025** | 0.25 (0.19, 0.30) | 0.65 (0.54, 0.77) | 1.64 (1.39, 1.88) | 4.03 (3.49, 4.55) | 9.03 (7.84, 10.43) | 16.26 (14.35, 18.51) | 23.42 (20.53, 26.69) |
| **2026** | 0.25 (0.19, 0.30) | 0.67 (0.55, 0.79) | 1.66 (1.41, 1.91) | 4.03 (3.47, 4.57) | 9.05 (7.80, 10.54) | 16.34 (14.34, 18.70) | 23.51 (20.46, 26.98) |
| **2027** | 0.25 (0.19, 0.31) | 0.69 (0.56, 0.81) | 1.69 (1.42, 1.94) | 4.03 (3.44, 4.59) | 9.07 (7.76, 10.64) | 16.42 (14.33, 18.90) | 23.61 (20.39, 27.26) |
| **2028** | 0.26 (0.19, 0.31) | 0.70 (0.56, 0.83) | 1.71 (1.43, 1.98) | 4.03 (3.42, 4.61) | 9.09 (7.71, 10.75) | 16.49 (14.32, 19.10) | 23.70 (20.32, 27.55) |
| **2029** | 0.26 (0.19, 0.32) | 0.72 (0.57, 0.86) | 1.74 (1.44, 2.01) | 4.03 (3.39, 4.63) | 9.11 (7.67, 10.85) | 16.57 (14.31, 19.29) | 23.79 (20.25, 27.84) |
| **2030** | 0.26 (0.20, 0.32) | 0.74 (0.58, 0.88) | 1.76 (1.46, 2.05) | 4.03 (3.37, 4.65) | 9.13 (7.63, 10.96) | 16.65 (14.30, 19.49) | 23.88 (20.18, 28.13) |

**STABLE 3 The DALY rate of global breast cancer burden in women from 1990 to 2030, by age groups.**

| **year** | **15-19 years** | **20-24 years** | **25-29 years** | **30-34 years** | **35-39 years** | **40-44 years** | **45-49 years** |
| --- | --- | --- | --- | --- | --- | --- | --- |
| **1990** | 11.79 (10.26, 13.56) | 27.36 (23.94, 30.53) | 84.79 (75.72, 92.79) | 257.79 (235.61, 279.47) | 528.79 (493.52, 566.89) | 872.29 (827.46, 921.32) | 1211.65 (1147.67, 1283.67) |
| **1991** | 12.09 (10.46, 13.95) | 27.68 (24.44, 30.76) | 84.11 (75.72, 91.79) | 258.16 (237.21, 277.27) | 528.05 (492.58, 565.73) | 869.95 (824.23, 920.02) | 1204.79 (1144.22, 1272.33) |
| **1992** | 12.43 (10.64, 14.44) | 28.31 (24.84, 31.40) | 84.53 (75.46, 92.48) | 258.69 (237.13, 277.47) | 529.36 (493.42, 563.36) | 872.03 (829.75, 919.13) | 1207.78 (1152.21, 1274.23) |
| **1993** | 12.72 (11.00, 14.70) | 28.80 (25.51, 32.03) | 84.66 (76.29, 92.33) | 258.36 (238.39, 277.72) | 535.08 (500.71, 572.76) | 879.36 (840.06, 926.47) | 1219.00 (1165.45, 1278.09) |
| **1994** | 12.88 (11.15, 14.94) | 29.81 (26.40, 32.88) | 88.61 (79.65, 96.86) | 264.32 (243.91, 284.45) | 539.67 (507.43, 574.01) | 881.05 (841.16, 923.11) | 1250.69 (1198.13, 1309.53) |
| **1995** | 13.08 (11.35, 15.22) | 30.80 (27.20, 34.15) | 91.86 (82.16, 100.79) | 267.61 (245.05, 288.95) | 538.99 (506.59, 572.88) | 871.56 (829.05, 916.93) | 1266.69 (1210.46, 1326.31) |
| **1996** | 13.22 (11.49, 15.25) | 31.40 (27.79, 34.63) | 92.74 (83.05, 101.32) | 266.19 (243.83, 287.86) | 531.98 (499.57, 565.24) | 860.68 (817.65, 905.59) | 1259.62 (1201.79, 1318.87) |
| **1997** | 13.24 (11.55, 15.24) | 31.90 (28.25, 35.21) | 93.61 (83.89, 102.10) | 265.55 (241.47, 287.67) | 527.27 (494.78, 559.47) | 854.61 (814.44, 896.36) | 1246.29 (1188.91, 1302.40) |
| **1998** | 13.21 (11.56, 15.16) | 32.57 (28.58, 36.11) | 95.76 (84.76, 105.49) | 267.55 (239.91, 290.50) | 521.34 (488.91, 553.11) | 853.15 (808.10, 896.47) | 1234.69 (1174.43, 1297.17) |
| **1999** | 13.32 (11.62, 15.36) | 33.13 (29.07, 36.79) | 96.93 (85.37, 107.12) | 267.94 (240.76, 292.03) | 517.46 (482.81, 549.67) | 856.14 (813.80, 900.51) | 1229.48 (1167.39, 1287.26) |
| **2000** | 13.43 (11.74, 15.38) | 33.40 (29.72, 36.79) | 97.81 (86.66, 107.40) | 268.40 (240.02, 291.64) | 514.32 (480.18, 546.89) | 848.36 (808.84, 888.08) | 1212.85 (1154.49, 1269.23) |
| **2001** | 13.29 (11.68, 15.15) | 33.35 (30.00, 36.60) | 98.61 (88.01, 107.92) | 267.21 (242.96, 288.76) | 510.42 (474.41, 545.21) | 840.25 (802.03, 878.73) | 1196.13 (1140.90, 1254.71) |
| **2002** | 13.17 (11.56, 15.09) | 32.82 (29.69, 35.96) | 95.24 (86.34, 103.99) | 254.21 (233.80, 273.55) | 501.78 (464.00, 535.79) | 833.28 (797.35, 873.04) | 1169.03 (1113.39, 1225.98) |
| **2003** | 13.19 (11.57, 15.08) | 32.06 (29.25, 35.16) | 92.14 (84.85, 99.09) | 245.66 (228.32, 262.35) | 488.52 (458.45, 520.02) | 818.72 (781.89, 856.20) | 1145.97 (1098.31, 1195.16) |
| **2004** | 13.13 (11.64, 14.98) | 31.88 (29.21, 34.78) | 91.55 (84.82, 98.25) | 243.71 (228.10, 259.42) | 479.36 (452.26, 505.71) | 800.62 (764.79, 837.04) | 1141.18 (1092.96, 1189.73) |
| **2005** | 13.19 (11.67, 15.07) | 31.92 (29.09, 34.88) | 91.82 (85.62, 98.39) | 241.08 (227.08, 255.86) | 477.66 (450.82, 503.70) | 790.21 (753.61, 829.38) | 1138.13 (1091.94, 1188.12) |
| **2006** | 13.14 (11.66, 15.01) | 31.65 (28.88, 34.52) | 90.89 (84.64, 97.79) | 238.56 (224.51, 252.59) | 473.54 (447.42, 500.47) | 776.44 (740.86, 813.10) | 1111.89 (1064.64, 1161.39) |
| **2007** | 13.20 (11.64, 15.12) | 31.35 (28.73, 34.36) | 90.03 (84.01, 97.13) | 237.10 (223.56, 252.10) | 467.46 (441.70, 493.30) | 767.45 (730.29, 805.22) | 1090.91 (1042.12, 1140.76) |
| **2008** | 13.56 (11.95, 15.51) | 31.39 (28.77, 34.28) | 90.93 (84.66, 97.78) | 239.19 (226.14, 253.65) | 464.90 (442.12, 489.83) | 763.76 (729.78, 799.43) | 1082.29 (1035.03, 1128.48) |
| **2009** | 13.87 (12.21, 15.95) | 31.57 (28.88, 34.69) | 92.34 (85.37, 100.00) | 241.28 (227.10, 256.95) | 462.61 (436.99, 491.00) | 751.41 (713.67, 791.70) | 1077.15 (1025.83, 1129.66) |
| **2010** | 14.18 (12.36, 16.36) | 32.15 (29.32, 35.52) | 93.73 (86.77, 101.68) | 243.31 (228.83, 260.43) | 463.05 (438.06, 490.64) | 744.62 (709.24, 784.80) | 1070.10 (1017.86, 1125.62) |
| **2011** | 14.45 (12.54, 16.71) | 32.62 (29.42, 35.87) | 93.43 (85.88, 101.43) | 243.07 (227.90, 259.32) | 462.71 (436.37, 488.75) | 741.77 (703.39, 780.81) | 1050.35 (999.77, 1104.43) |
| **2012** | 14.65 (12.63, 17.00) | 32.76 (29.35, 36.25) | 91.87 (84.50, 99.67) | 239.53 (224.70, 255.96) | 461.54 (435.16, 488.48) | 739.41 (701.91, 780.11) | 1021.70 (974.47, 1075.08) |
| **2013** | 15.10 (13.01, 17.54) | 33.23 (29.93, 37.05) | 92.14 (84.26, 100.90) | 240.79 (224.52, 258.13) | 469.19 (441.23, 498.04) | 748.95 (704.71, 793.05) | 1013.05 (958.42, 1067.10) |
| **2014** | 15.43 (13.23, 18.18) | 33.66 (29.89, 37.71) | 91.38 (83.25, 99.84) | 240.63 (224.11, 259.36) | 471.56 (441.90, 502.54) | 757.93 (708.19, 809.86) | 1007.37 (950.83, 1063.85) |
| **2015** | 15.72 (13.40, 18.60) | 34.50 (30.29, 38.90) | 91.30 (82.48, 100.88) | 240.78 (222.86, 259.25) | 476.57 (446.49, 510.62) | 763.06 (712.01, 818.94) | 1005.59 (948.99, 1069.61) |
| **2016** | 15.87 (13.42, 19.12) | 35.32 (30.80, 40.01) | 91.97 (82.87, 102.47) | 240.56 (220.47, 260.42) | 479.60 (446.92, 516.08) | 767.86 (712.00, 823.11) | 1008.44 (942.88, 1079.39) |
| **2017** | 16.12 (13.60, 19.43) | 36.26 (31.57, 41.26) | 93.09 (83.66, 103.90) | 240.01 (219.77, 261.64) | 479.80 (443.44, 517.77) | 772.15 (710.43, 834.24) | 1008.95 (937.70, 1080.94) |
| **2018** | 16.54 (13.85, 20.08) | 37.71 (32.77, 43.38) | 95.34 (85.23, 106.71) | 241.24 (218.57, 264.36) | 481.63 (439.57, 525.33) | 777.07 (709.91, 848.63) | 1013.23 (935.79, 1100.21) |
| **2019** | 16.66 (13.72, 20.13) | 38.62 (33.06, 44.32) | 96.71 (85.40, 108.04) | 240.55 (216.64, 263.57) | 482.44 (438.57, 528.65) | 779.27 (713.10, 852.20) | 1017.67 (927.42, 1108.77) |
| **2020** | 16.95 (13.90, 20.63) | 39.72 (33.91, 45.87) | 97.99 (86.24, 109.97) | 240.78 (215.36, 265.30) | 484.46 (436.65, 534.10) | 784.60 (712.98, 863.71) | 1020.76 (924.49, 1119.91) |
| **2021** | 17.21 (13.98, 21.00) | 40.85 (34.67, 47.35) | 99.59 (87.10, 111.97) | 240.87 (213.90, 266.44) | 485.74 (434.21, 538.89) | 788.58 (713.54, 872.99) | 1025.12 (920.24, 1132.15) |
| **2022** | 17.46 (14.07, 21.36) | 41.97 (35.43, 48.83) | 101.20 (87.97, 113.97) | 240.96 (212.44, 267.57) | 487.02 (431.76, 543.67) | 792.57 (714.10, 882.27) | 1029.48 (916.00, 1144.40) |
| **2023** | 17.71 (14.15, 21.73) | 43.10 (36.19, 50.31) | 102.81 (88.84, 115.96) | 241.05 (210.98, 268.70) | 488.31 (429.32, 548.45) | 796.55 (714.66, 891.55) | 1033.85 (911.75, 1156.64) |
| **2024** | 17.96 (14.23, 22.09) | 44.23 (36.95, 51.79) | 104.41 (89.71, 117.96) | 241.13 (209.52, 269.84) | 489.59 (426.87, 553.24) | 800.53 (715.22, 900.83) | 1038.21 (907.51, 1168.89) |
| **2025** | 18.22 (14.32, 22.46) | 45.36 (37.71, 53.27) | 106.02 (90.58, 119.96) | 241.22 (208.06, 270.97) | 490.88 (424.43, 558.02) | 804.51 (715.78, 910.11) | 1042.58 (903.26, 1181.13) |
| **2026** | 18.47 (14.40, 22.82) | 46.49 (38.48, 54.75) | 107.62 (91.45, 121.96) | 241.31 (206.60, 272.11) | 492.16 (421.98, 562.81) | 808.49 (716.34, 919.39) | 1046.94 (899.02, 1193.38) |
| **2027** | 18.72 (14.49, 23.18) | 47.61 (39.24, 56.23) | 109.23 (92.32, 123.95) | 241.40 (205.13, 273.24) | 493.44 (419.54, 567.59) | 812.47 (716.90, 928.67) | 1051.31 (894.77, 1205.62) |
| **2028** | 18.98 (14.57, 23.55) | 48.74 (40.00, 57.71) | 110.84 (93.18, 125.95) | 241.49 (203.67, 274.37) | 494.73 (417.09, 572.38) | 816.45 (717.47, 937.95) | 1055.67 (890.53, 1217.87) |
| **2029** | 19.23 (14.65, 23.91) | 49.87 (40.76, 59.19) | 112.44 (94.05, 127.95) | 241.57 (202.21, 275.51) | 496.01 (414.65, 577.16) | 820.43 (718.03, 947.23) | 1060.04 (886.28, 1230.11) |
| **2030** | 19.48 (14.74, 24.28) | 51.00 (41.52, 60.67) | 114.05 (94.92, 129.95) | 241.66 (200.75, 276.64) | 497.30 (412.20, 581.95) | 824.41 (718.59, 956.51) | 1064.40 (882.04, 1242.36) |

**STABLE 4 The Age-standerized rates trends of global breast cancer in burden women from 1990 to 2030.**

| year | Incidence | Deaths | DALYs (Disability-Adjusted Life Years) |
| --- | --- | --- | --- |
| 1990 | 40.12 (38.78, 41.33) | 17.76 (16.93, 18.51) | 524.87 (501.78, 551.15) |
| 1992 | 40.54 (39.17, 41.78) | 17.76 (16.93, 18.49) | 525.41 (503.95, 549.65) |
| 1991 | 40.97 (39.60, 42.19) | 17.73 (16.90, 18.51) | 524.08 (501.78, 548.34) |
| 1994 | 42.08 (40.70, 43.30) | 17.96 (17.21, 18.65) | 534.19 (514.31, 554.92) |
| 1993 | 42.54 (41.10, 43.71) | 18.00 (17.19, 18.65) | 531.27 (511.88, 554.37) |
| 1997 | 42.83 (41.45, 44.03) | 17.69 (16.90, 18.33) | 525.38 (504.77, 546.47) |
| 1998 | 42.84 (41.40, 43.93) | 17.93 (17.14, 18.59) | 521.67 (499.93, 543.73) |
| 1996 | 42.92 (41.38, 44.05) | 17.55 (16.74, 18.23) | 528.92 (508.72, 551.00) |
| 1995 | 43.12 (41.74, 44.21) | 17.81 (17.01, 18.45) | 532.79 (511.98, 554.36) |
| 1999 | 43.54 (42.17, 44.67) | 17.57 (16.79, 18.22) | 521.18 (499.67, 542.65) |
| 2002 | 43.62 (42.25, 44.82) | 17.43 (16.66, 18.05) | 511.35 (490.82, 532.57) |
| 2000 | 43.77 (42.20, 45.05) | 17.31 (16.52, 17.91) | 517.99 (497.93, 539.14) |
| 2001 | 44.04 (42.58, 45.32) | 17.12 (16.31, 17.73) | 514.44 (493.93, 533.98) |
| 2003 | 44.31 (42.85, 45.51) | 17.25 (16.42, 17.89) | 506.69 (488.00, 526.88) |
| 2006 | 44.07 (42.44, 45.42) | 16.86 (16.04, 17.44) | 489.79 (471.19, 510.10) |
| 2004 | 44.33 (42.59, 45.68) | 16.41 (15.57, 16.99) | 499.62 (479.06, 519.05) |
| 2005 | 44.05 (42.32, 45.35) | 16.78 (15.92, 17.37) | 497.01 (476.82, 517.28) |
| 2007 | 44.20 (42.43, 45.48) | 16.55 (15.68, 17.14) | 485.09 (465.72, 505.47) |
| 2010 | 44.66 (42.86, 46.01) | 16.11 (15.19, 16.76) | 478.79 (459.11, 499.58) |
| 2014 | 44.92 (43.15, 46.22) | 16.34 (15.47, 16.96) | 472.10 (447.94, 495.38) |
| 2009 | 45.07 (43.34, 46.56) | 16.21 (15.35, 16.87) | 479.57 (457.51, 501.04) |
| 2008 | 45.12 (43.35, 46.83) | 16.18 (15.32, 16.83) | 482.95 (464.02, 501.68) |
| 2011 | 44.81 (42.79, 46.45) | 15.99 (15.07, 16.63) | 476.20 (455.32, 496.99) |
| 2012 | 44.83 (42.88, 46.51) | 16.03 (15.04, 16.81) | 471.92 (450.11, 493.45) |
| 2013 | 44.97 (42.92, 46.78) | 15.95 (14.97, 16.67) | 471.53 (447.96, 494.16) |
| 2018 | 45.28 (43.14, 47.25) | 15.94 (15.04, 16.69) | 473.56 (441.20, 507.62) |
| 2015 | 45.27 (43.02, 47.40) | 15.88 (14.66, 17.07) | 474.83 (450.27, 500.64) |
| 2016 | 45.31 (42.45, 48.21) | 15.89 (14.69, 16.92) | 474.63 (447.06, 501.60) |
| 2017 | 45.66 (42.35, 49.00) | 15.98 (14.97, 16.87) | 473.11 (442.59, 503.14) |
| 2019 | 45.86 (41.91, 49.76) | 15.89 (14.77, 16.87) | 473.83 (437.30, 510.51) |
| 2020 | 46.03 (41.69, 50.40) | 15.85 (14.55, 17.12) | 473.87 (435.03, 513.54) |
| 2021 | 46.23 (41.38, 51.15) | 15.83 (14.46, 17.19) | 473.91 (432.08, 516.75) |
| 2022 | 46.44 (41.06, 51.90) | 15.81 (14.37, 17.27) | 473.95 (429.12, 519.97) |
| 2023 | 46.65 (40.75, 52.65) | 15.78 (14.27, 17.35) | 473.99 (426.17, 523.19) |
| 2024 | 46.85 (40.43, 53.40) | 15.76 (14.18, 17.43) | 474.04 (423.22, 526.40) |
| 2025 | 47.06 (40.12, 54.14) | 15.74 (14.09, 17.50) | 474.08 (420.26, 529.62) |
| 2026 | 47.27 (39.80, 54.89) | 15.71 (14.00, 17.58) | 474.12 (417.31, 532.84) |
| 2027 | 47.47 (39.49, 55.64) | 15.69 (13.91, 17.66) | 474.16 (414.35, 536.05) |
| 2028 | 47.68 (39.18, 56.39) | 15.67 (13.82, 17.74) | 474.20 (411.40, 539.27) |
| 2029 | 47.89 (38.86, 57.14) | 15.64 (13.72, 17.82) | 474.24 (408.44, 542.48) |
| 2030 | 48.09 (38.55, 57.89) | 15.62 (13.63, 17.89) | 474.28 (405.49, 545.70) |

**STABLE 5 The Age-standerized rates of global breast cancer burden in women in 2020 and 2030, by countries.**

|  | 2020 | | | 2030 | | | |
| --- | --- | --- | --- | --- | --- | --- | --- |
| location | Incidence | Deaths | DALYs (Disability-Adjusted Life Years) | | Incidence | Deaths | DALYs (Disability-Adjusted Life Years) |
| Afghanistan | 22.72 (17.14, 29.60) | 16.70 (12.68, 21.59) | 512.46 (384.79, 668.81) | | 26.60 (20.50, 34.79) | 18.54 (14.41, 24.37) | 562.60 (430.37, 726.82) |
| Albania | 39.40 (28.53, 53.77) | 11.93 (8.77, 16.13) | 374.74 (272.42, 517.11) | | 42.45 (32.56, 61.33) | 11.68 (8.77, 17.22) | 364.79 (280.86, 555.98) |
| Algeria | 34.59 (25.82, 44.46) | 13.85 (10.49, 17.47) | 418.64 (310.22, 545.23) | | 42.31 (28.75, 58.13) | 14.26 (9.62, 19.03) | 429.35 (280.96, 615.65) |
| American Samoa | 55.99 (43.76, 71.11) | 31.91 (25.30, 39.84) | 893.92 (698.30, 1134.44) | | 55.49 (36.92, 78.55) | 32.04 (21.99, 43.80) | 892.00 (596.68, 1254.55) |
| Andorra | 80.62 (55.99, 108.46) | 17.23 (12.27, 23.08) | 504.63 (359.02, 691.39) | | 78.39 (49.11, 110.35) | 16.65 (11.37, 23.21) | 486.55 (319.77, 735.67) |
| Angola | 27.22 (19.90, 36.73) | 20.39 (15.22, 27.24) | 569.35 (405.68, 778.38) | | 34.92 (23.25, 50.33) | 24.51 (16.78, 35.08) | 673.55 (416.43, 983.61) |
| Antigua and Barbuda | 81.09 (68.56, 96.32) | 30.11 (26.03, 34.93) | 809.03 (687.07, 962.19) | | 84.69 (68.75, 108.85) | 27.64 (24.21, 33.87) | 757.13 (610.60, 1005.09) |
| Argentina | 63.73 (45.93, 84.88) | 28.00 (25.85, 30.29) | 752.16 (693.68, 817.32) | | 69.86 (20.61, 131.25) | 27.48 (24.30, 31.72) | 751.53 (638.41, 887.32) |
| Armenia | 61.74 (48.73, 76.23) | 23.82 (19.11, 29.13) | 701.55 (554.20, 870.58) | | 67.75 (33.33, 105.66) | 22.89 (10.50, 37.20) | 687.43 (290.73, 1154.37) |
| Australia | 81.03 (58.83, 109.51) | 16.99 (15.41, 18.39) | 488.39 (445.92, 540.15) | | 77.43 (19.18, 157.41) | 16.60 (14.38, 18.95) | 467.61 (400.49, 556.80) |
| Austria | 68.36 (52.96, 86.49) | 17.16 (15.69, 18.50) | 461.15 (421.33, 501.86) | | 57.90 (15.28, 110.72) | 14.53 (12.67, 16.57) | 389.05 (324.00, 454.25) |
| Azerbaijan | 39.01 (30.38, 48.70) | 17.79 (13.92, 22.17) | 550.73 (426.72, 691.83) | | 43.47 (26.29, 62.34) | 17.29 (10.47, 25.35) | 557.91 (314.97, 848.36) |
| Bahamas | 91.37 (72.06, 116.35) | 35.53 (28.50, 44.96) | 1077.45 (845.02, 1383.29) | | 88.42 (57.24, 133.69) | 32.01 (20.76, 49.92) | 994.33 (619.86, 1558.18) |
| Bahrain | 68.65 (54.63, 85.51) | 25.22 (20.41, 30.83) | 669.02 (528.70, 839.55) | | 80.42 (58.90, 109.63) | 25.58 (19.24, 34.30) | 671.55 (472.99, 946.89) |
| Bangladesh | 25.64 (19.74, 32.77) | 14.70 (11.48, 18.44) | 453.62 (349.79, 578.78) | | 32.17 (22.91, 43.02) | 16.24 (11.62, 20.93) | 488.74 (347.59, 647.47) |
| Barbados | 101.95 (80.52, 124.22) | 35.84 (28.62, 43.10) | 992.90 (773.43, 1215.41) | | 100.77 (65.37, 134.30) | 33.68 (21.41, 45.44) | 926.54 (543.20, 1287.30) |
| Belarus | 49.82 (36.07, 68.60) | 14.04 (10.27, 18.74) | 435.47 (315.10, 596.57) | | 59.22 (27.50, 107.42) | 14.99 (6.08, 26.65) | 475.89 (196.70, 886.89) |
| Belgium | 94.52 (69.02, 127.84) | 22.81 (20.72, 24.80) | 623.24 (571.16, 692.72) | | 93.89 (26.28, 189.57) | 21.56 (18.48, 24.79) | 606.05 (516.83, 747.28) |
| Belize | 26.85 (22.35, 32.16) | 11.74 (9.85, 14.02) | 352.90 (292.66, 422.70) | | 27.79 (19.63, 38.11) | 11.28 (7.79, 15.79) | 338.22 (224.27, 467.97) |
| Benin | 21.38 (15.49, 28.69) | 15.85 (11.67, 20.64) | 446.98 (318.45, 610.93) | | 23.64 (17.11, 32.01) | 16.52 (12.00, 21.35) | 464.07 (332.41, 638.26) |
| Bermuda | 80.56 (64.35, 102.85) | 19.27 (15.50, 24.39) | 514.66 (415.45, 657.11) | | 81.61 (58.53, 117.64) | 17.98 (13.11, 26.10) | 497.68 (367.26, 735.38) |
| Bhutan | 21.26 (14.82, 29.35) | 12.11 (8.56, 16.49) | 349.88 (237.68, 485.06) | | 25.39 (17.98, 35.69) | 12.88 (9.11, 17.81) | 366.59 (242.91, 513.94) |
| Bolivia (Plurinational State of) | 35.57 (26.77, 47.60) | 19.19 (14.87, 24.73) | 541.30 (399.49, 719.96) | | 43.67 (31.60, 63.05) | 20.75 (15.81, 27.63) | 578.49 (401.33, 818.90) |
| Bosnia and Herzegovina | 55.66 (40.49, 74.94) | 20.80 (15.64, 27.19) | 575.32 (421.44, 772.13) | | 58.42 (19.17, 112.25) | 20.16 (6.75, 37.59) | 553.87 (151.45, 1111.13) |
| Botswana | 49.25 (31.88, 72.88) | 28.52 (19.06, 41.03) | 788.01 (501.92, 1182.67) | | 51.57 (34.42, 78.78) | 27.28 (18.77, 39.14) | 741.39 (488.88, 1176.41) |
| Brazil | 39.24 (36.50, 42.07) | 14.85 (13.80, 15.88) | 440.47 (412.76, 472.43) | | 35.73 (30.11, 42.30) | 12.02 (10.42, 14.27) | 351.62 (292.90, 427.11) |
| Brunei Darussalam | 68.22 (55.41, 83.43) | 22.78 (18.99, 27.15) | 737.59 (605.26, 889.11) | | 70.72 (50.29, 94.37) | 20.31 (14.66, 26.95) | 696.53 (495.64, 921.26) |
| Bulgaria | 78.20 (56.64, 104.56) | 22.70 (16.80, 29.67) | 689.88 (498.15, 924.66) | | 76.44 (23.56, 144.28) | 19.30 (4.62, 37.40) | 594.95 (111.75, 1209.18) |
| Burkina Faso | 27.77 (21.96, 34.41) | 20.97 (16.82, 25.62) | 579.88 (449.16, 732.18) | | 32.25 (26.59, 39.56) | 23.23 (19.56, 28.64) | 647.44 (513.88, 808.02) |
| Burundi | 20.83 (14.57, 28.89) | 16.23 (11.37, 22.46) | 466.96 (324.21, 652.04) | | 22.34 (15.89, 29.31) | 17.11 (11.98, 22.61) | 489.54 (349.37, 635.47) |
| Cabo Verde | 28.53 (22.55, 35.66) | 15.72 (12.19, 19.48) | 407.08 (322.33, 504.29) | | 34.64 (23.34, 48.66) | 17.33 (10.77, 24.38) | 430.11 (283.65, 601.64) |
| Cambodia | 24.06 (18.22, 30.05) | 14.01 (10.77, 17.29) | 446.59 (337.92, 564.82) | | 29.23 (21.16, 37.00) | 15.28 (11.12, 19.25) | 477.93 (350.91, 612.86) |
| Cameroon | 31.35 (21.56, 45.30) | 22.06 (15.68, 31.08) | 621.36 (424.19, 909.70) | | 35.84 (25.09, 52.29) | 22.97 (17.15, 32.95) | 634.81 (454.77, 963.57) |
| Canada | 90.98 (67.06, 121.41) | 18.03 (16.36, 19.64) | 513.35 (463.50, 565.90) | | 95.96 (32.81, 180.83) | 18.62 (16.22, 21.43) | 526.95 (437.47, 616.47) |
| Central African Republic | 23.69 (15.05, 38.66) | 20.19 (13.05, 32.56) | 591.48 (375.15, 952.91) | | 23.59 (14.85, 39.00) | 20.05 (12.76, 32.14) | 579.64 (394.26, 880.58) |
| Chad | 17.89 (12.57, 23.82) | 14.15 (10.09, 18.74) | 405.31 (279.46, 547.47) | | 19.43 (13.42, 26.01) | 14.82 (10.54, 19.96) | 423.04 (288.66, 591.24) |
| Chile | 39.30 (28.86, 52.50) | 13.83 (12.54, 15.20) | 373.37 (334.81, 413.40) | | 38.44 (10.65, 75.90) | 12.91 (11.14, 15.49) | 339.16 (262.77, 421.54) |
| China | 36.75 (28.53, 46.75) | 9.11 (7.15, 11.40) | 281.16 (223.39, 349.56) | | 48.61 (32.43, 70.19) | 10.09 (6.44, 14.60) | 315.16 (211.61, 445.90) |
| Colombia | 39.55 (28.29, 53.27) | 11.57 (8.42, 15.54) | 357.27 (253.36, 490.22) | | 39.42 (10.11, 77.18) | 9.88 (1.74, 21.06) | 303.66 (24.20, 689.44) |
| Comoros | 26.70 (19.35, 35.59) | 19.72 (14.64, 25.99) | 554.15 (391.90, 749.70) | | 26.50 (15.55, 38.85) | 18.94 (11.42, 27.26) | 493.47 (255.53, 748.69) |
| Congo | 40.93 (24.54, 60.74) | 30.06 (18.46, 43.90) | 840.81 (500.61, 1253.72) | | 45.50 (27.10, 69.63) | 31.66 (19.40, 48.36) | 867.97 (511.41, 1306.98) |
| Cook Islands | 91.85 (67.33, 119.60) | 38.29 (29.96, 48.01) | 1033.93 (763.45, 1347.35) | | 101.91 (65.13, 145.16) | 38.76 (27.44, 52.17) | 1028.25 (662.75, 1492.48) |
| Costa Rica | 54.62 (39.74, 72.00) | 15.42 (11.49, 20.20) | 432.16 (315.31, 579.55) | | 55.72 (17.32, 102.14) | 13.91 (4.08, 27.06) | 396.42 (87.26, 807.46) |
| Croatia | 65.99 (47.00, 89.70) | 19.11 (13.93, 25.02) | 498.38 (356.03, 670.81) | | 49.94 (2.50, 113.08) | 12.55 (-0.36, 27.87) | 341.69 (-23.51, 793.47) |
| Cuba | 60.13 (46.87, 76.11) | 17.56 (14.04, 21.94) | 481.27 (371.60, 610.74) | | 61.47 (22.50, 109.90) | 15.75 (5.80, 29.52) | 435.49 (97.60, 846.58) |
| Cyprus | 100.30 (81.15, 123.45) | 21.89 (18.78, 25.26) | 585.93 (489.40, 696.43) | | 91.17 (56.61, 137.82) | 19.39 (14.48, 24.52) | 533.67 (372.41, 722.20) |
| Czechia | 57.98 (45.00, 73.62) | 16.13 (12.82, 20.05) | 423.29 (330.25, 534.60) | | 55.40 (18.34, 101.51) | 14.78 (5.60, 26.85) | 384.66 (106.67, 733.42) |
| Côte d'Ivoire | 21.54 (15.81, 28.66) | 16.17 (12.19, 20.94) | 448.96 (323.67, 601.38) | | 23.10 (15.89, 32.61) | 16.33 (11.48, 21.74) | 442.47 (305.28, 615.69) |
| Democratic People's Republic of Korea | 26.73 (18.57, 37.53) | 12.97 (9.46, 17.35) | 426.17 (292.40, 596.31) | | 28.98 (20.14, 41.56) | 12.76 (9.53, 16.84) | 422.50 (281.48, 604.10) |
| Democratic Republic of the Congo | 29.61 (20.58, 41.08) | 23.03 (15.93, 31.74) | 646.52 (454.42, 885.48) | | 37.36 (23.25, 57.07) | 27.52 (16.80, 42.14) | 766.03 (496.19, 1133.74) |
| Denmark | 84.01 (60.60, 111.91) | 22.55 (20.10, 24.70) | 586.41 (521.61, 654.93) | | 78.29 (18.64, 152.95) | 20.96 (17.40, 24.06) | 558.14 (442.43, 685.39) |
| Djibouti | 28.40 (19.55, 38.79) | 20.38 (14.90, 27.55) | 555.61 (370.46, 783.08) | | 36.10 (24.81, 46.34) | 23.93 (18.33, 30.51) | 652.36 (430.69, 859.78) |
| Dominica | 65.22 (51.40, 82.83) | 30.79 (24.91, 38.71) | 863.07 (670.30, 1106.06) | | 65.98 (48.68, 90.26) | 30.99 (23.66, 42.51) | 868.66 (609.86, 1201.53) |
| Dominican Republic | 35.81 (24.68, 49.41) | 15.58 (11.12, 21.03) | 469.43 (322.21, 649.52) | | 31.02 (13.97, 54.19) | 11.17 (4.77, 20.27) | 313.17 (109.49, 598.35) |
| Ecuador | 31.49 (23.81, 41.82) | 13.10 (10.06, 17.20) | 381.85 (285.06, 508.81) | | 35.44 (17.14, 62.75) | 13.12 (5.98, 23.92) | 389.06 (153.52, 727.17) |
| Egypt | 29.92 (19.58, 42.10) | 14.12 (9.03, 19.72) | 433.31 (280.95, 610.76) | | 34.21 (14.90, 59.36) | 12.25 (3.91, 22.22) | 384.77 (121.51, 725.92) |
| El Salvador | 30.57 (21.13, 42.84) | 10.81 (7.66, 14.78) | 328.86 (226.61, 459.45) | | 35.56 (18.06, 60.92) | 11.33 (5.87, 19.04) | 337.45 (160.50, 589.76) |
| Equatorial Guinea | 40.15 (22.64, 65.88) | 27.32 (15.74, 43.58) | 709.50 (387.11, 1173.37) | | 49.65 (25.94, 81.14) | 31.71 (16.85, 50.76) | 805.77 (387.68, 1298.11) |
| Eritrea | 29.30 (20.87, 40.85) | 22.81 (16.40, 31.67) | 652.44 (458.08, 927.33) | | 34.37 (23.74, 49.34) | 25.53 (17.56, 36.86) | 725.45 (491.86, 1084.26) |
| Estonia | 66.52 (48.20, 88.36) | 17.25 (12.80, 22.61) | 504.07 (362.47, 676.54) | | 68.84 (24.31, 125.66) | 16.48 (5.66, 30.61) | 493.70 (137.58, 960.90) |
| Eswatini | 31.65 (19.69, 46.89) | 22.86 (14.79, 33.20) | 608.81 (373.21, 933.75) | | 32.79 (24.22, 46.95) | 22.18 (17.86, 32.20) | 580.83 (443.13, 885.90) |
| Ethiopia | 23.58 (18.82, 28.94) | 17.64 (14.50, 20.80) | 473.26 (376.28, 579.83) | | 29.16 (22.51, 37.09) | 20.20 (16.80, 22.78) | 530.66 (407.81, 651.50) |
| Fiji | 59.98 (43.82, 80.16) | 37.24 (27.53, 49.17) | 1079.06 (781.20, 1439.72) | | 67.22 (45.67, 100.22) | 38.33 (25.64, 57.60) | 1107.84 (724.84, 1663.89) |
| Finland | 83.87 (59.82, 113.25) | 16.05 (14.47, 17.63) | 460.59 (411.45, 515.45) | | 78.18 (15.58, 157.44) | 14.79 (12.60, 17.56) | 433.51 (354.93, 525.92) |
| France | 85.83 (61.46, 116.87) | 19.67 (17.61, 21.36) | 557.99 (502.66, 618.36) | | 80.56 (16.35, 168.05) | 18.29 (15.77, 20.56) | 515.91 (423.67, 627.47) |
| Gabon | 40.78 (26.03, 57.14) | 27.99 (18.16, 38.57) | 736.19 (472.60, 1044.12) | | 48.33 (29.02, 66.82) | 30.44 (19.25, 40.15) | 788.78 (490.77, 1119.94) |
| Gambia | 17.80 (11.54, 25.35) | 12.62 (8.15, 18.07) | 369.02 (236.34, 534.89) | | 23.33 (14.30, 33.56) | 15.65 (9.17, 23.18) | 458.76 (272.01, 686.87) |
| Georgia | 66.69 (53.33, 82.08) | 27.99 (22.43, 34.22) | 869.15 (689.44, 1066.54) | | 74.59 (40.76, 114.64) | 27.12 (12.71, 43.43) | 870.93 (400.13, 1386.62) |
| Germany | 84.20 (59.96, 115.48) | 20.87 (18.95, 22.45) | 569.95 (517.97, 623.93) | | 78.38 (13.90, 170.81) | 18.97 (15.90, 21.57) | 528.60 (435.57, 622.70) |
| Ghana | 46.19 (35.82, 59.38) | 30.74 (24.36, 38.86) | 874.18 (669.22, 1131.94) | | 55.89 (41.92, 73.79) | 33.66 (26.27, 42.75) | 929.08 (682.69, 1230.30) |
| Greece | 86.35 (62.09, 116.85) | 21.80 (19.81, 23.62) | 587.53 (537.38, 645.99) | | 94.97 (29.80, 180.62) | 23.90 (20.72, 27.07) | 630.74 (550.38, 739.69) |
| Greenland | 45.39 (34.85, 57.90) | 19.28 (14.99, 24.34) | 552.78 (416.85, 715.04) | | 46.94 (30.35, 67.03) | 18.78 (11.98, 26.45) | 536.82 (313.17, 806.33) |
| Grenada | 72.29 (62.09, 82.30) | 30.37 (26.72, 34.06) | 863.85 (743.93, 994.91) | | 77.24 (60.44, 90.86) | 28.45 (23.41, 32.90) | 791.38 (610.20, 990.78) |
| Guam | 38.29 (30.86, 47.42) | 17.62 (14.46, 21.75) | 516.74 (418.01, 639.98) | | 43.78 (32.17, 59.27) | 19.95 (15.24, 27.51) | 565.93 (417.08, 768.85) |
| Guatemala | 22.07 (16.69, 29.07) | 10.52 (8.19, 13.50) | 304.25 (229.97, 397.22) | | 21.99 (11.74, 36.89) | 9.39 (5.14, 15.96) | 273.87 (130.62, 478.60) |
| Guinea | 24.95 (17.93, 33.44) | 19.30 (13.98, 25.55) | 555.44 (394.20, 758.13) | | 26.66 (17.82, 36.56) | 19.57 (13.26, 26.11) | 554.81 (361.74, 779.28) |
| Guinea-Bissau | 29.59 (20.23, 43.08) | 22.31 (15.54, 32.35) | 665.34 (451.19, 972.62) | | 30.43 (19.52, 46.40) | 22.02 (14.29, 33.93) | 642.81 (412.46, 971.78) |
| Guyana | 49.42 (36.44, 64.66) | 24.88 (18.92, 32.15) | 755.86 (551.42, 1001.23) | | 50.89 (30.50, 74.91) | 22.71 (14.47, 34.17) | 674.25 (373.49, 1068.35) |
| Haiti | 43.61 (28.15, 62.24) | 28.74 (18.78, 40.62) | 881.86 (565.04, 1289.76) | | 45.28 (29.34, 62.09) | 28.36 (18.82, 39.41) | 858.23 (568.77, 1240.50) |
| Honduras | 27.55 (19.94, 37.78) | 13.41 (10.25, 17.63) | 380.20 (274.49, 520.12) | | 30.03 (23.59, 39.61) | 12.83 (10.90, 15.21) | 378.46 (302.79, 464.54) |
| Hungary | 64.29 (49.95, 81.15) | 19.91 (15.71, 24.66) | 552.07 (430.00, 696.77) | | 63.61 (21.57, 114.79) | 17.42 (4.78, 32.60) | 493.83 (116.80, 954.82) |
| Iceland | 66.63 (55.65, 78.43) | 13.91 (11.93, 15.81) | 407.14 (353.83, 461.56) | | 59.70 (43.91, 77.01) | 11.28 (9.10, 13.65) | 343.91 (277.03, 406.24) |
| India | 23.51 (17.72, 30.15) | 13.83 (10.46, 17.82) | 420.68 (316.78, 544.30) | | 27.74 (16.88, 40.92) | 14.88 (8.88, 22.53) | 443.22 (256.38, 687.39) |
| Indonesia | 37.69 (28.88, 49.40) | 20.44 (15.80, 26.24) | 701.59 (545.05, 914.98) | | 41.00 (30.33, 58.77) | 20.14 (14.81, 28.43) | 668.87 (510.61, 945.84) |
| Iran (Islamic Republic of) | 34.93 (31.38, 39.08) | 11.92 (10.80, 13.23) | 370.01 (335.49, 408.00) | | 42.42 (36.17, 49.56) | 12.01 (10.10, 14.39) | 370.65 (307.51, 433.72) |
| Iraq | 52.98 (39.82, 70.29) | 21.70 (16.55, 28.18) | 715.49 (535.37, 961.44) | | 64.29 (50.23, 84.17) | 22.87 (18.53, 28.66) | 721.84 (570.66, 967.49) |
| Ireland | 94.32 (67.34, 125.69) | 20.19 (18.21, 22.24) | 566.61 (506.09, 638.47) | | 85.13 (16.40, 166.31) | 17.32 (14.12, 20.75) | 494.56 (389.63, 635.96) |
| Israel | 78.98 (56.68, 105.06) | 21.36 (19.46, 23.10) | 581.75 (531.53, 635.46) | | 80.91 (22.47, 153.28) | 21.20 (18.95, 23.69) | 569.28 (482.19, 667.92) |
| Italy | 88.52 (63.47, 119.48) | 18.31 (16.63, 19.47) | 525.69 (486.14, 567.69) | | 84.01 (16.59, 172.57) | 17.31 (15.36, 19.06) | 492.96 (434.69, 559.34) |
| Jamaica | 78.72 (57.54, 103.63) | 29.14 (21.89, 37.42) | 903.97 (666.59, 1193.60) | | 75.30 (34.88, 125.74) | 27.72 (13.88, 44.55) | 799.01 (356.94, 1406.20) |
| Japan | 59.67 (46.19, 75.87) | 9.97 (9.07, 10.63) | 332.71 (308.99, 361.84) | | 51.11 (11.79, 100.36) | 8.34 (7.46, 9.35) | 282.52 (252.09, 329.68) |
| Jordan | 53.33 (40.44, 68.80) | 19.84 (15.27, 25.36) | 582.71 (443.30, 754.86) | | 57.50 (35.85, 85.49) | 19.39 (12.29, 28.71) | 575.45 (361.26, 877.36) |
| Kazakhstan | 40.09 (32.84, 47.91) | 15.95 (13.35, 18.83) | 477.00 (393.14, 574.62) | | 39.98 (21.53, 59.72) | 13.24 (6.59, 20.55) | 409.77 (193.25, 664.09) |
| Kenya | 23.49 (16.95, 31.56) | 17.22 (12.73, 23.18) | 483.59 (351.00, 666.30) | | 29.06 (20.30, 37.86) | 19.03 (14.19, 26.11) | 524.43 (375.24, 752.37) |
| Kiribati | 46.64 (34.09, 64.60) | 34.89 (25.85, 48.38) | 1048.43 (749.65, 1451.67) | | 52.24 (40.23, 76.87) | 37.08 (28.73, 54.96) | 1102.19 (806.76, 1602.98) |
| Kuwait | 42.65 (33.50, 55.69) | 12.79 (10.08, 16.74) | 353.48 (277.65, 464.01) | | 45.41 (27.88, 67.47) | 12.46 (7.61, 18.49) | 344.35 (198.88, 532.15) |
| Kyrgyzstan | 22.34 (18.73, 26.45) | 10.60 (9.02, 12.46) | 311.25 (261.77, 370.54) | | 22.48 (13.35, 33.42) | 9.02 (4.96, 14.12) | 272.64 (143.26, 436.54) |
| Lao People's Democratic Republic | 31.68 (22.57, 44.72) | 20.88 (15.05, 29.14) | 651.85 (458.65, 929.90) | | 35.86 (24.76, 48.70) | 21.73 (14.44, 29.41) | 662.09 (449.38, 908.31) |
| Latvia | 55.77 (39.36, 77.64) | 18.27 (12.98, 25.12) | 535.11 (368.68, 747.73) | | 57.42 (22.65, 109.10) | 16.31 (4.47, 32.62) | 496.55 (126.35, 1003.09) |
| Lebanon | 124.09 (92.15, 163.81) | 35.50 (27.08, 46.88) | 1069.04 (800.07, 1420.41) | | 138.40 (91.64, 196.01) | 35.27 (24.61, 50.78) | 1079.84 (701.26, 1569.03) |
| Lesotho | 36.68 (23.06, 55.51) | 27.93 (17.72, 41.31) | 771.20 (468.66, 1186.03) | | 35.13 (22.23, 52.65) | 24.88 (14.42, 36.52) | 683.65 (431.62, 1037.20) |
| Liberia | 25.59 (17.31, 39.04) | 18.77 (13.04, 28.40) | 528.09 (351.32, 812.11) | | 27.79 (16.95, 44.91) | 19.47 (12.25, 31.28) | 544.75 (319.83, 892.67) |
| Libya | 41.57 (28.95, 57.96) | 17.37 (12.51, 23.83) | 555.23 (393.08, 770.85) | | 44.35 (26.13, 68.40) | 18.28 (11.81, 27.57) | 584.05 (364.11, 893.66) |
| Lithuania | 49.46 (37.88, 63.22) | 16.18 (12.49, 20.53) | 483.09 (366.00, 624.68) | | 42.98 (10.95, 81.82) | 12.40 (1.75, 25.55) | 373.49 (29.97, 804.02) |
| Luxembourg | 76.83 (60.27, 96.88) | 18.59 (15.69, 21.97) | 507.84 (430.97, 594.83) | | 60.69 (32.96, 95.80) | 15.22 (11.40, 20.25) | 414.95 (305.70, 538.44) |
| Madagascar | 22.37 (15.79, 30.54) | 17.07 (12.32, 22.87) | 502.75 (352.93, 684.67) | | 26.43 (17.08, 37.77) | 19.25 (12.73, 26.61) | 560.06 (369.08, 776.60) |
| Malawi | 22.46 (16.61, 28.99) | 17.41 (13.21, 22.07) | 464.10 (334.34, 610.39) | | 26.33 (18.33, 35.64) | 19.58 (14.22, 26.12) | 518.85 (351.07, 728.56) |
| Malaysia | 61.06 (44.83, 79.41) | 26.20 (19.38, 33.82) | 767.94 (565.86, 995.57) | | 77.47 (41.39, 116.41) | 30.17 (15.30, 46.36) | 872.30 (445.17, 1342.09) |
| Maldives | 34.32 (26.98, 42.05) | 13.74 (11.08, 16.83) | 375.56 (297.65, 464.22) | | 41.05 (29.66, 52.93) | 14.88 (11.38, 20.03) | 404.27 (299.06, 546.23) |
| Mali | 23.59 (17.55, 31.04) | 17.13 (13.06, 22.36) | 510.15 (373.64, 667.89) | | 26.62 (19.31, 37.30) | 18.26 (13.78, 25.98) | 546.05 (382.01, 751.32) |
| Malta | 80.65 (65.18, 99.28) | 20.29 (17.33, 23.74) | 583.76 (501.72, 683.41) | | 77.94 (50.70, 113.42) | 17.12 (12.70, 23.00) | 511.88 (388.52, 681.02) |
| Marshall Islands | 58.93 (39.02, 88.02) | 39.82 (27.18, 58.64) | 1207.02 (794.40, 1812.45) | | 64.26 (41.98, 99.27) | 41.16 (27.90, 62.77) | 1234.00 (812.05, 1887.47) |
| Mauritania | 26.79 (18.54, 35.79) | 18.16 (12.88, 23.87) | 489.15 (332.28, 661.03) | | 29.52 (15.79, 44.30) | 18.25 (9.88, 26.58) | 478.90 (224.67, 749.10) |
| Mauritius | 54.77 (42.11, 69.15) | 22.38 (17.58, 28.13) | 689.04 (529.97, 875.11) | | 59.22 (24.16, 100.16) | 21.95 (8.76, 38.86) | 668.00 (213.54, 1208.48) |
| Mexico | 37.33 (29.48, 46.88) | 12.48 (10.02, 15.63) | 382.58 (305.49, 480.14) | | 40.87 (14.40, 74.94) | 11.96 (4.08, 22.98) | 368.90 (115.98, 707.54) |
| Micronesia (Federated States of) | 67.03 (39.52, 109.26) | 42.47 (26.85, 66.99) | 1246.23 (708.72, 2053.87) | | 76.29 (39.75, 128.44) | 45.79 (26.52, 74.74) | 1327.22 (638.35, 2256.37) |
| Monaco | 147.66 (105.65, 193.00) | 31.61 (23.05, 40.15) | 911.41 (662.36, 1194.92) | | 126.26 (70.42, 179.90) | 26.74 (15.54, 37.57) | 770.96 (465.05, 1125.47) |
| Mongolia | 18.13 (13.00, 24.51) | 9.98 (7.32, 13.34) | 294.22 (209.36, 401.27) | | 21.14 (13.35, 30.52) | 10.54 (6.86, 15.36) | 314.58 (197.28, 468.41) |
| Montenegro | 86.08 (66.52, 107.64) | 26.09 (20.78, 32.04) | 766.24 (595.85, 962.56) | | 84.45 (51.81, 118.08) | 22.54 (13.99, 32.03) | 693.34 (412.93, 1028.20) |
| Morocco | 53.60 (38.88, 74.19) | 24.50 (17.97, 33.07) | 844.52 (613.19, 1168.40) | | 64.64 (44.96, 93.29) | 25.07 (17.11, 35.92) | 853.66 (589.52, 1228.71) |
| Mozambique | 27.70 (19.33, 38.84) | 21.80 (15.64, 30.26) | 595.44 (398.82, 853.64) | | 31.94 (20.95, 49.51) | 23.96 (16.00, 36.63) | 646.60 (383.15, 1032.37) |
| Myanmar | 24.20 (19.33, 30.75) | 15.09 (12.40, 18.90) | 447.38 (354.82, 572.79) | | 6.34 (4.51, 9.34) | 2.20 (1.21, 3.91) | 0.15 (-20.44, 56.17) |
| Namibia | 48.74 (33.41, 71.21) | 29.67 (20.81, 41.95) | 865.02 (581.14, 1289.19) | | 51.14 (35.54, 79.50) | 28.35 (19.96, 41.61) | 805.46 (545.43, 1324.43) |
| Nauru | 76.74 (50.42, 105.85) | 41.26 (28.31, 55.34) | 1232.54 (806.03, 1707.77) | | 88.40 (54.17, 125.18) | 40.65 (24.78, 56.72) | 1221.11 (741.05, 1703.36) |
| Nepal | 29.48 (21.54, 39.46) | 18.33 (13.47, 24.25) | 556.56 (403.90, 752.04) | | 35.94 (24.30, 50.47) | 20.39 (13.66, 28.12) | 611.87 (410.53, 889.84) |
| Netherlands | 112.61 (82.19, 151.03) | 24.13 (21.98, 26.18) | 671.70 (608.44, 740.90) | | 117.63 (38.50, 225.28) | 23.82 (21.07, 26.75) | 683.68 (573.62, 811.69) |
| New Zealand | 101.48 (73.25, 137.26) | 19.37 (17.74, 20.95) | 596.93 (543.61, 655.09) | | 99.86 (26.91, 198.25) | 19.06 (16.92, 21.66) | 580.07 (493.27, 681.60) |
| Nicaragua | 33.37 (26.39, 42.43) | 12.10 (9.90, 14.80) | 336.14 (263.45, 431.55) | | 37.34 (18.99, 63.80) | 11.87 (6.28, 19.37) | 326.52 (122.55, 616.51) |
| Niger | 14.62 (9.95, 21.82) | 11.31 (7.84, 16.46) | 320.08 (215.14, 479.05) | | 16.07 (10.88, 25.30) | 11.99 (8.42, 18.04) | 342.53 (227.11, 532.73) |
| Nigeria | 38.98 (26.86, 53.33) | 27.04 (19.45, 36.35) | 745.72 (523.28, 1023.09) | | 41.73 (27.05, 56.76) | 27.56 (19.63, 38.81) | 737.38 (508.65, 991.60) |
| Niue | 67.95 (44.03, 93.97) | 30.05 (20.08, 40.43) | 871.77 (564.91, 1203.61) | | 71.73 (42.83, 100.74) | 29.78 (18.41, 40.32) | 865.81 (490.04, 1202.06) |
| North Macedonia | 73.20 (52.22, 98.70) | 26.33 (19.57, 34.24) | 741.88 (530.79, 985.46) | | 75.02 (27.00, 136.36) | 24.74 (9.36, 43.18) | 705.71 (226.96, 1264.46) |
| Northern Mariana Islands | 65.96 (51.65, 83.50) | 27.48 (22.03, 34.33) | 770.76 (605.67, 982.32) | | 68.75 (49.19, 89.41) | 25.82 (18.77, 34.97) | 721.97 (520.87, 986.54) |
| Norway | 65.77 (50.31, 85.32) | 14.99 (13.59, 16.32) | 412.61 (378.10, 455.39) | | 52.86 (10.18, 109.90) | 12.79 (11.45, 14.40) | 349.37 (304.09, 412.39) |
| Oman | 44.64 (36.36, 53.04) | 15.65 (12.98, 18.75) | 428.45 (348.57, 515.23) | | 46.16 (33.30, 58.65) | 13.27 (9.76, 17.13) | 386.08 (270.09, 519.03) |
| Pakistan | 77.52 (56.78, 104.48) | 52.27 (39.21, 70.74) | 1578.14 (1181.21, 2165.46) | | 86.91 (60.46, 123.84) | 54.56 (40.93, 78.85) | 1622.52 (1207.44, 2447.20) |
| Palau | 76.03 (55.58, 100.52) | 34.07 (25.49, 43.43) | 974.36 (711.67, 1274.28) | | 83.53 (59.16, 113.10) | 34.78 (25.26, 45.18) | 988.85 (710.35, 1286.65) |
| Palestine | 58.80 (47.61, 71.73) | 25.88 (21.09, 31.40) | 751.19 (609.92, 905.97) | | 75.71 (51.77, 103.13) | 29.30 (19.79, 40.67) | 856.84 (565.88, 1160.84) |
| Panama | 43.54 (31.06, 58.21) | 13.30 (9.64, 17.63) | 395.38 (276.99, 533.62) | | 45.53 (12.00, 84.60) | 10.99 (1.62, 22.86) | 331.06 (11.66, 715.20) |
| Papua New Guinea | 65.26 (48.20, 87.93) | 43.75 (32.65, 57.97) | 1476.44 (1082.22, 1969.58) | | 72.05 (53.56, 100.02) | 45.93 (34.61, 62.37) | 1543.58 (1108.74, 2059.37) |
| Paraguay | 45.60 (32.22, 60.72) | 18.21 (13.20, 23.98) | 534.90 (377.14, 716.14) | | 55.31 (26.11, 88.25) | 18.82 (8.50, 31.19) | 567.75 (236.56, 958.67) |
| Peru | 28.02 (20.02, 38.52) | 10.76 (7.86, 14.45) | 317.61 (225.03, 432.64) | | 32.05 (19.34, 50.43) | 10.79 (6.58, 16.87) | 321.81 (184.08, 501.42) |
| Philippines | 41.01 (29.76, 55.27) | 23.00 (16.81, 30.64) | 715.06 (515.51, 957.05) | | 44.72 (23.86, 72.42) | 23.32 (12.13, 38.20) | 712.29 (343.03, 1180.10) |
| Poland | 55.12 (40.12, 73.60) | 18.98 (14.36, 24.96) | 518.70 (389.27, 682.65) | | 50.46 (10.50, 102.91) | 16.72 (4.37, 34.50) | 451.77 (89.60, 930.09) |
| Portugal | 73.67 (52.60, 100.28) | 16.36 (14.91, 17.95) | 480.67 (435.15, 538.95) | | 75.51 (19.74, 149.71) | 15.67 (13.85, 18.87) | 468.73 (392.28, 588.52) |
| Puerto Rico | 71.83 (51.59, 97.11) | 17.07 (12.52, 22.69) | 522.10 (372.52, 704.01) | | 77.68 (26.77, 146.25) | 18.22 (6.47, 34.02) | 560.93 (161.73, 1070.03) |
| Qatar | 102.71 (78.71, 131.50) | 36.13 (28.03, 45.26) | 836.35 (639.55, 1061.04) | | 91.04 (65.51, 130.71) | 27.27 (18.80, 38.13) | 645.08 (444.72, 896.89) |
| Republic of Korea | 46.54 (36.72, 57.63) | 8.67 (7.59, 9.77) | 279.57 (243.45, 317.93) | | 48.27 (24.23, 75.84) | 8.39 (6.63, 10.46) | 271.36 (200.44, 347.55) |
| Republic of Moldova | 37.62 (31.16, 46.08) | 14.53 (12.02, 17.58) | 432.92 (357.80, 530.88) | | 23.30 (7.54, 45.83) | 7.05 (0.37, 15.33) | 195.72 (-1.15, 460.56) |
| Romania | 51.47 (40.20, 64.50) | 18.44 (14.53, 23.14) | 538.75 (416.54, 682.54) | | 53.43 (19.84, 93.49) | 17.01 (4.79, 32.60) | 500.04 (108.27, 974.23) |
| Russian Federation | 53.78 (41.60, 68.56) | 16.64 (13.01, 20.69) | 496.86 (391.21, 622.41) | | 49.86 (7.43, 100.88) | 14.33 (2.32, 28.12) | 423.29 (70.71, 845.71) |
| Rwanda | 27.83 (20.96, 36.68) | 20.22 (15.61, 25.79) | 554.62 (411.24, 740.38) | | 33.97 (25.85, 46.05) | 23.05 (18.52, 29.27) | 626.14 (482.48, 849.00) |
| Saint Kitts and Nevis | 83.91 (60.31, 110.32) | 29.72 (23.03, 37.80) | 793.94 (554.64, 1059.70) | | 86.55 (51.76, 131.83) | 27.94 (18.52, 41.62) | 736.66 (404.05, 1174.30) |
| Saint Lucia | 55.96 (46.22, 68.29) | 22.01 (18.14, 26.39) | 639.74 (522.54, 777.25) | | 61.48 (46.53, 84.14) | 23.00 (16.10, 30.56) | 636.82 (438.94, 870.00) |
| Saint Vincent and the Grenadines | 68.01 (57.25, 81.16) | 30.49 (25.93, 35.67) | 887.01 (745.18, 1055.52) | | 69.41 (50.88, 95.16) | 27.58 (19.71, 37.11) | 818.02 (575.76, 1138.44) |
| Samoa | 44.84 (29.70, 70.94) | 24.64 (16.80, 38.72) | 748.90 (491.70, 1178.00) | | 53.21 (34.75, 78.74) | 26.06 (17.96, 37.39) | 782.82 (498.56, 1104.08) |
| San Marino | 84.22 (61.38, 117.09) | 19.44 (12.47, 29.49) | 555.47 (357.39, 867.90) | | 81.67 (55.76, 119.13) | 18.07 (10.68, 28.63) | 527.58 (333.51, 913.00) |
| Sao Tome and Principe | 32.88 (20.38, 49.35) | 21.12 (13.08, 31.38) | 599.49 (370.59, 900.46) | | 38.92 (20.94, 60.16) | 22.77 (12.00, 35.55) | 638.23 (339.46, 997.28) |
| Saudi Arabia | 43.96 (32.01, 59.10) | 14.35 (10.64, 19.05) | 447.51 (324.91, 599.85) | | 51.82 (32.98, 78.78) | 14.19 (8.50, 22.12) | 454.95 (269.70, 705.16) |
| Senegal | 29.53 (20.92, 38.84) | 21.45 (15.39, 28.22) | 607.32 (418.06, 806.82) | | 33.31 (19.33, 46.21) | 22.67 (12.50, 32.37) | 634.41 (320.77, 902.81) |
| Serbia | 83.49 (60.94, 111.90) | 28.94 (21.73, 37.65) | 783.58 (575.53, 1050.45) | | 84.39 (24.40, 163.31) | 27.45 (8.39, 51.53) | 753.30 (202.30, 1515.16) |
| Seychelles | 55.93 (45.35, 67.19) | 25.06 (20.49, 30.07) | 761.72 (616.96, 927.38) | | 63.84 (45.11, 83.12) | 25.14 (17.60, 33.65) | 755.91 (505.95, 1053.56) |
| Sierra Leone | 24.48 (17.33, 35.70) | 18.01 (13.01, 26.36) | 519.08 (361.69, 759.04) | | 28.28 (19.27, 43.59) | 19.52 (13.65, 31.34) | 564.79 (377.92, 861.96) |
| Singapore | 61.30 (46.66, 78.17) | 12.28 (11.01, 13.46) | 375.43 (338.59, 420.32) | | 51.50 (12.46, 99.26) | 9.52 (7.73, 11.54) | 298.29 (233.81, 375.29) |
| Slovakia | 60.27 (41.37, 83.57) | 18.43 (13.00, 25.18) | 503.27 (349.71, 700.59) | | 57.39 (12.34, 116.58) | 15.86 (3.11, 33.17) | 447.93 (86.58, 963.14) |
| Slovenia | 64.39 (46.28, 89.76) | 17.49 (12.82, 23.95) | 466.64 (335.69, 646.44) | | 62.89 (20.64, 131.17) | 16.13 (5.39, 33.73) | 437.81 (127.12, 930.25) |
| Solomon Islands | 146.50 (113.24, 188.92) | 86.18 (67.73, 108.75) | 3044.81 (2339.25, 3913.00) | | 297.60 (225.89, 388.99) | 168.75 (131.78, 213.49) | 6087.21 (4604.45, 7891.12) |
| Somalia | 15.05 (9.08, 24.02) | 12.98 (8.06, 20.67) | 376.76 (234.01, 595.23) | | 15.58 (9.24, 24.78) | 13.30 (8.53, 20.17) | 381.97 (239.13, 588.30) |
| South Africa | 31.11 (26.63, 35.96) | 19.74 (17.22, 22.54) | 508.02 (435.07, 584.01) | | 20.19 (12.87, 27.18) | 11.37 (7.13, 16.04) | 291.95 (184.79, 370.65) |
| South Sudan | 16.13 (10.86, 22.34) | 13.26 (8.95, 18.23) | 361.93 (231.94, 516.21) | | 18.47 (12.28, 25.18) | 14.63 (10.09, 19.30) | 399.54 (257.06, 555.30) |
| Spain | 69.81 (50.42, 94.30) | 15.11 (13.73, 16.34) | 442.16 (401.71, 485.79) | | 69.85 (20.19, 139.26) | 14.94 (13.42, 16.91) | 436.23 (366.66, 515.99) |
| Sri Lanka | 30.24 (21.48, 41.57) | 12.11 (8.66, 16.39) | 344.87 (243.57, 472.46) | | 34.10 (21.71, 50.54) | 11.67 (6.97, 17.39) | 338.54 (204.62, 503.79) |
| Sudan | 24.78 (16.75, 33.72) | 13.31 (9.58, 17.82) | 422.19 (285.19, 580.23) | | 32.26 (21.43, 42.80) | 15.14 (11.08, 18.93) | 474.30 (313.43, 620.84) |
| Suriname | 40.41 (31.49, 50.38) | 19.32 (15.31, 23.73) | 566.37 (441.46, 712.32) | | 47.13 (30.80, 66.90) | 20.93 (13.67, 29.16) | 618.35 (393.21, 896.14) |
| Sweden | 81.17 (62.21, 103.73) | 17.48 (15.90, 18.88) | 479.88 (439.69, 520.43) | | 68.87 (13.77, 136.53) | 14.54 (12.25, 16.95) | 398.04 (333.51, 459.29) |
| Switzerland | 77.37 (55.24, 104.91) | 17.71 (15.68, 19.31) | 478.27 (429.78, 531.96) | | 74.91 (17.68, 150.79) | 16.46 (13.48, 18.85) | 454.64 (379.48, 542.98) |
| Syrian Arab Republic | 27.17 (18.93, 37.87) | 11.33 (8.04, 15.69) | 335.88 (237.14, 479.16) | | 30.00 (19.20, 42.32) | 11.47 (7.41, 16.45) | 343.94 (234.51, 514.57) |
| Taiwan (Province of China) | 48.76 (34.29, 68.31) | 11.36 (8.23, 15.55) | 379.31 (271.94, 525.80) | | 43.32 (5.40, 98.39) | 9.31 (0.88, 21.53) | 314.76 (23.60, 743.20) |
| Tajikistan | 26.27 (20.06, 34.00) | 15.22 (11.78, 19.43) | 451.78 (342.62, 583.46) | | 28.14 (15.49, 44.81) | 14.67 (8.08, 23.68) | 454.18 (230.59, 745.20) |
| Thailand | 33.91 (23.42, 46.87) | 12.48 (8.71, 16.98) | 397.12 (273.81, 549.94) | | 41.43 (19.23, 71.78) | 13.45 (5.52, 23.96) | 435.00 (178.64, 796.76) |
| Timor-Leste | 25.31 (17.74, 34.96) | 16.44 (11.89, 22.66) | 497.01 (334.90, 697.70) | | 28.34 (21.05, 38.10) | 17.80 (13.56, 24.16) | 540.00 (379.79, 731.75) |
| Togo | 25.06 (19.03, 33.56) | 17.91 (13.83, 23.75) | 507.97 (378.24, 681.56) | | 30.34 (23.03, 41.57) | 20.13 (15.77, 27.63) | 568.28 (426.30, 768.89) |
| Tokelau | 65.05 (44.43, 91.02) | 33.88 (23.32, 46.14) | 1011.88 (685.12, 1419.00) | | 75.53 (50.01, 110.29) | 35.66 (23.59, 49.63) | 1056.67 (683.47, 1533.88) |
| Tonga | 56.34 (41.19, 74.43) | 32.57 (24.36, 42.57) | 959.95 (700.83, 1285.83) | | 64.34 (44.64, 86.52) | 34.59 (24.65, 47.10) | 1012.30 (705.37, 1434.22) |
| Trinidad and Tobago | 57.85 (41.71, 78.24) | 23.63 (17.51, 31.08) | 684.80 (488.01, 921.94) | | 59.52 (40.48, 84.77) | 22.64 (16.02, 30.56) | 655.92 (439.74, 913.81) |
| Tunisia | 46.81 (33.41, 63.02) | 15.74 (11.43, 20.80) | 484.18 (344.25, 650.39) | | 54.32 (39.45, 72.84) | 15.67 (11.69, 20.84) | 486.95 (354.25, 649.79) |
| Turkey | 36.58 (27.99, 46.98) | 12.61 (9.77, 15.90) | 368.72 (281.06, 472.42) | | 41.79 (24.91, 62.67) | 12.22 (7.04, 18.40) | 357.33 (192.51, 557.85) |
| Turkmenistan | 28.39 (21.37, 38.20) | 12.28 (9.27, 16.12) | 415.73 (310.92, 562.15) | | 24.50 (10.82, 46.90) | 9.63 (3.66, 17.98) | 300.21 (97.38, 628.72) |
| Tuvalu | 58.61 (40.41, 84.54) | 35.31 (24.66, 49.81) | 1046.26 (714.67, 1514.95) | | 66.04 (44.85, 95.52) | 36.91 (24.85, 50.48) | 1073.58 (733.40, 1550.69) |
| Uganda | 32.07 (23.86, 41.15) | 22.72 (17.27, 29.11) | 664.12 (489.49, 874.22) | | 38.55 (26.60, 52.54) | 25.52 (18.32, 35.69) | 734.69 (494.75, 1061.50) |
| Ukraine | 45.69 (33.37, 60.57) | 19.99 (14.69, 26.26) | 627.44 (460.27, 826.43) | | 48.93 (14.52, 91.94) | 21.11 (5.99, 40.07) | 654.28 (175.18, 1247.37) |
| United Arab Emirates | 57.39 (43.07, 73.95) | 25.70 (19.58, 33.12) | 781.08 (585.45, 1012.22) | | 64.53 (48.27, 85.15) | 25.73 (19.13, 34.66) | 767.17 (573.69, 1022.64) |
| United Kingdom | 94.81 (69.98, 125.47) | 22.60 (21.02, 23.64) | 614.56 (580.05, 656.05) | | 95.90 (25.24, 188.29) | 22.81 (20.95, 24.13) | 616.73 (568.51, 687.26) |
| United Republic of Tanzania | 24.41 (18.96, 30.18) | 18.13 (14.32, 21.85) | 478.99 (365.63, 601.74) | | 30.17 (21.75, 38.35) | 20.79 (14.99, 25.22) | 543.33 (374.27, 718.50) |
| United States of America | 93.71 (74.05, 118.18) | 18.28 (17.20, 19.14) | 534.22 (500.97, 571.48) | | 91.08 (26.31, 176.49) | 18.05 (16.78, 19.35) | 518.86 (469.78, 579.13) |
| United States Virgin Islands | 73.85 (57.59, 92.83) | 27.95 (22.37, 34.20) | 751.59 (576.35, 940.12) | | 70.60 (49.83, 94.92) | 26.83 (19.67, 35.71) | 732.42 (490.69, 948.92) |
| Uruguay | 72.75 (52.96, 96.48) | 29.83 (27.28, 32.29) | 805.87 (736.45, 884.95) | | 74.93 (20.61, 141.17) | 28.33 (24.89, 32.09) | 762.61 (643.37, 921.47) |
| Uzbekistan | 37.45 (29.72, 46.26) | 17.23 (13.88, 20.96) | 531.74 (420.43, 657.68) | | 37.15 (16.81, 60.29) | 13.90 (5.01, 24.09) | 459.36 (160.85, 800.12) |
| Vanuatu | 38.65 (25.15, 57.36) | 27.43 (17.90, 39.47) | 823.03 (532.79, 1232.05) | | 42.75 (28.11, 64.38) | 29.16 (18.10, 40.87) | 871.01 (587.32, 1338.25) |
| Venezuela (Bolivarian Republic of) | 52.77 (37.13, 72.86) | 17.54 (12.64, 23.80) | 534.50 (376.76, 745.38) | | 52.85 (25.30, 91.45) | 17.86 (9.28, 29.63) | 524.28 (252.74, 941.51) |
| Viet Nam | 49.92 (37.12, 65.20) | 21.74 (16.38, 28.38) | 659.55 (491.23, 863.04) | | 62.23 (44.36, 85.99) | 23.47 (16.20, 33.77) | 709.76 (498.86, 1015.11) |
| Yemen | 23.15 (16.98, 31.97) | 13.70 (10.29, 18.68) | 443.91 (322.14, 619.57) | | 26.72 (19.15, 37.95) | 15.80 (11.89, 22.00) | 519.90 (366.36, 738.96) |
| Zambia | 27.94 (20.74, 37.72) | 19.67 (14.92, 26.17) | 557.96 (402.85, 752.56) | | 34.00 (24.87, 47.76) | 22.00 (16.27, 30.59) | 609.79 (435.91, 854.50) |
| Zimbabwe | 37.91 (26.14, 52.47) | 26.94 (18.51, 37.65) | 782.74 (525.31, 1109.74) | | 39.32 (25.30, 58.16) | 26.52 (16.13, 40.67) | 768.15 (477.81, 1201.40) |

**STABLE 6 The EAPC of global breast cancer burden in women from 1990 to 2030, by countries.**

|  | 1990-2019 EAPC | | | 2020-2030 EAPC | | |
| --- | --- | --- | --- | --- | --- | --- |
| location | Incidence | Deaths | DALYs (Disability-Adjusted Life Years) | Incidence | Deaths | DALYs (Disability-Adjusted Life Years) |
| Afghanistan | 0.91 (0.85, 0.95) | 0.53 (0.44, 0.58) | 0.37 (0.36, 0.40) | 1.59 (1.81, 1.63) | 1.05 (1.29, 1.22) | 0.94 (1.13, 0.84) |
| Albania | 2.89 (1.91, 3.72) | 0.86 (0.04, 1.58) | 0.95 (-0.02, 1.78) | 0.75 (1.33, 1.32) | -0.21 (0.00, 0.65) | -0.27 (0.31, 0.73) |
| Algeria | 1.44 (1.43, 1.31) | 0.20 (0.18, 0.11) | 0.04 (0.04, -0.07) | 2.03 (1.08, 2.71) | 0.30 (-0.87, 0.86) | 0.25 (-0.99, 1.22) |
| American Samoa | 1.84 (1.94, 1.90) | 1.13 (1.20, 1.21) | 1.24 (1.35, 1.27) | -0.09 (-1.68, 1.00) | 0.04 (-1.39, 0.95) | -0.02 (-1.56, 1.01) |
| Andorra | 0.74 (0.68, 0.60) | -0.59 (-0.66, -0.77) | -0.55 (-0.60, -0.68) | -0.28 (-1.30, 0.17) | -0.34 (-0.75, 0.06) | -0.36 (-1.15, 0.62) |
| Angola | 1.64 (2.05, 1.46) | 1.13 (1.50, 0.96) | 0.91 (1.21, 0.76) | 2.52 (1.57, 3.19) | 1.85 (0.98, 2.56) | 1.69 (0.26, 2.36) |
| Antigua and Barbuda | 1.49 (1.35, 1.61) | 0.72 (0.58, 0.82) | 0.48 (0.32, 0.58) | 0.44 (0.03, 1.23) | -0.85 (-0.72, -0.31) | -0.66 (-1.17, 0.44) |
| Argentina | 0.45 (0.12, 0.76) | -0.53 (-0.60, -0.47) | -0.68 (-0.75, -0.60) | 0.92 (-7.59, 4.43) | -0.19 (-0.62, 0.46) | -0.01 (-0.83, 0.83) |
| Armenia | -0.26 (-0.45, -0.08) | -1.25 (-1.38, -1.12) | -1.70 (-1.89, -1.51) | 0.93 (-3.71, 3.31) | -0.40 (-5.76, 2.47) | -0.20 (-6.19, 2.86) |
| Australia | -0.27 (-0.66, 0.11) | -1.51 (-1.67, -1.40) | -1.70 (-1.81, -1.56) | -0.45 (-10.31, 3.68) | -0.23 (-0.68, 0.30) | -0.43 (-1.07, 0.30) |
| Austria | -0.53 (-0.71, -0.36) | -1.30 (-1.42, -1.21) | -1.72 (-1.82, -1.63) | -1.65 (-11.30, 2.50) | -1.65 (-2.11, -1.10) | -1.68 (-2.59, -0.99) |
| Azerbaijan | 0.61 (0.30, 0.91) | -0.17 (-0.52, 0.15) | -0.57 (-0.88, -0.29) | 1.09 (-1.44, 2.50) | -0.28 (-2.80, 1.35) | 0.13 (-2.98, 2.06) |
| Bahamas | 0.94 (0.73, 1.17) | 0.33 (0.14, 0.53) | 0.18 (-0.02, 0.40) | -0.33 (-2.27, 1.40) | -1.04 (-3.11, 1.05) | -0.80 (-3.04, 1.20) |
| Bahrain | 1.25 (1.11, 1.34) | -0.44 (-0.57, -0.36) | -0.85 (-1.01, -0.74) | 1.59 (0.76, 2.51) | 0.14 (-0.59, 1.07) | 0.04 (-1.11, 1.21) |
| Bangladesh | 0.53 (0.63, 0.55) | -0.46 (-0.33, -0.44) | -0.73 (-0.62, -0.73) | 2.29 (1.50, 2.75) | 1.00 (0.13, 1.28) | 0.75 (-0.06, 1.13) |
| Barbados | 1.57 (1.33, 1.80) | 0.78 (0.55, 0.96) | 0.59 (0.31, 0.80) | -0.12 (-2.06, 0.78) | -0.62 (-2.86, 0.53) | -0.69 (-3.46, 0.58) |
| Belarus | -0.27 (-0.70, 0.16) | -1.50 (-1.85, -1.15) | -1.93 (-2.37, -1.49) | 1.74 (-2.67, 4.56) | 0.66 (-5.07, 3.58) | 0.89 (-4.58, 4.03) |
| Belgium | -0.41 (-0.71, -0.14) | -1.63 (-1.74, -1.54) | -1.92 (-2.02, -1.82) | -0.07 (-9.01, 4.00) | -0.56 (-1.14, -0.00) | -0.28 (-0.99, 0.76) |
| Belize | 1.35 (1.28, 1.42) | 0.55 (0.47, 0.63) | 0.65 (0.59, 0.72) | 0.34 (-1.29, 1.71) | -0.40 (-2.31, 1.20) | -0.42 (-2.62, 1.02) |
| Benin | 1.08 (0.86, 1.31) | 0.66 (0.50, 0.79) | 0.61 (0.35, 0.85) | 1.01 (1.00, 1.10) | 0.42 (0.28, 0.33) | 0.38 (0.43, 0.44) |
| Bermuda | -1.01 (-1.29, -0.65) | -2.62 (-2.94, -2.24) | -2.70 (-2.97, -2.36) | 0.13 (-0.94, 1.35) | -0.69 (-1.66, 0.68) | -0.33 (-1.22, 1.13) |
| Bhutan | 1.02 (0.84, 0.94) | -0.14 (-0.26, -0.22) | -0.59 (-0.81, -0.62) | 1.79 (1.94, 1.97) | 0.62 (0.63, 0.78) | 0.47 (0.22, 0.58) |
| Bolivia (Plurinational State of) | 1.32 (1.29, 1.35) | 0.27 (0.24, 0.28) | -0.01 (-0.03, -0.01) | 2.07 (1.67, 2.84) | 0.79 (0.61, 1.11) | 0.67 (0.05, 1.30) |
| Bosnia and Herzegovina | 2.99 (3.08, 2.89) | 1.63 (1.75, 1.51) | 1.13 (1.27, 0.98) | 0.49 (-7.11, 4.11) | -0.31 (-7.94, 3.28) | -0.38 (-9.50, 3.69) |
| Botswana | 2.36 (2.71, 2.23) | 1.33 (1.65, 1.23) | 1.46 (1.90, 1.27) | 0.46 (0.77, 0.78) | -0.44 (-0.15, -0.47) | -0.61 (-0.26, -0.05) |
| Brazil | 0.68 (0.63, 0.71) | -0.64 (-0.71, -0.61) | -0.58 (-0.62, -0.55) | -0.93 (-1.90, 0.06) | -2.09 (-2.76, -1.06) | -2.23 (-3.36, -1.00) |
| Brunei Darussalam | 2.18 (2.28, 2.12) | 1.13 (1.21, 1.04) | 0.93 (1.06, 0.84) | 0.36 (-0.96, 1.24) | -1.14 (-2.55, -0.07) | -0.57 (-1.98, 0.36) |
| Bulgaria | 2.24 (1.90, 2.55) | 1.52 (1.23, 1.79) | 1.19 (0.89, 1.46) | -0.23 (-8.25, 3.26) | -1.61 (-11.69, 2.34) | -1.47 (-13.25, 2.71) |
| Burkina Faso | 0.78 (0.88, 0.79) | 0.36 (0.48, 0.32) | 0.34 (0.41, 0.33) | 1.50 (1.93, 1.40) | 1.03 (1.52, 1.12) | 1.11 (1.35, 0.99) |
| Burundi | -0.23 (-0.43, -0.02) | -0.56 (-0.78, -0.33) | -0.81 (-0.95, -0.59) | 0.70 (0.87, 0.14) | 0.53 (0.52, 0.07) | 0.47 (0.75, -0.26) |
| Cabo Verde | 1.42 (1.54, 1.27) | 0.39 (0.48, 0.24) | -0.00 (0.16, -0.20) | 1.96 (0.35, 3.15) | 0.98 (-1.23, 2.27) | 0.55 (-1.27, 1.78) |
| Cambodia | 1.67 (1.65, 1.55) | 0.72 (0.77, 0.57) | 0.48 (0.44, 0.36) | 1.96 (1.50, 2.10) | 0.87 (0.32, 1.08) | 0.68 (0.38, 0.82) |
| Cameroon | 1.30 (0.77, 1.83) | 0.82 (0.38, 1.25) | 0.71 (0.16, 1.24) | 1.35 (1.53, 1.45) | 0.41 (0.90, 0.58) | 0.21 (0.70, 0.58) |
| Canada | -0.57 (-0.84, -0.30) | -1.52 (-1.67, -1.40) | -1.71 (-1.83, -1.59) | 0.53 (-6.82, 4.05) | 0.32 (-0.09, 0.88) | 0.26 (-0.58, 0.86) |
| Central African Republic | 0.30 (-0.16, 1.08) | 0.20 (-0.19, 0.95) | 0.14 (-0.37, 0.92) | -0.04 (-0.13, 0.09) | -0.07 (-0.23, -0.13) | -0.20 (0.50, -0.79) |
| Chad | 1.18 (1.00, 1.14) | 0.92 (0.78, 0.88) | 0.86 (0.65, 0.81) | 0.83 (0.66, 0.88) | 0.47 (0.43, 0.64) | 0.43 (0.32, 0.77) |
| Chile | 1.12 (0.81, 1.38) | -0.69 (-0.81, -0.63) | -0.76 (-0.85, -0.69) | -0.22 (-9.29, 3.74) | -0.69 (-1.17, 0.19) | -0.96 (-2.39, 0.20) |
| China | 2.70 (2.63, 2.77) | -0.13 (-0.19, -0.07) | -0.35 (-0.35, -0.31) | 2.83 (1.29, 4.13) | 1.03 (-1.04, 2.50) | 1.15 (-0.54, 2.46) |
| Colombia | 1.06 (0.69, 1.39) | -0.75 (-1.07, -0.46) | -0.59 (-0.94, -0.27) | -0.03 (-9.55, 3.76) | -1.57 (-13.84, 3.08) | -1.61 (-18.91, 3.46) |
| Comoros | 1.46 (2.86, 1.16) | 0.91 (2.17, 0.62) | 0.78 (2.43, 0.46) | -0.08 (-2.16, 0.88) | -0.40 (-2.45, 0.48) | -1.15 (-4.17, -0.01) |
| Congo | 0.95 (0.53, 1.19) | 0.42 (0.06, 0.64) | 0.30 (-0.07, 0.55) | 1.06 (1.00, 1.38) | 0.52 (0.50, 0.97) | 0.32 (0.21, 0.42) |
| Cook Islands | 0.63 (0.62, 0.49) | -0.14 (-0.04, -0.30) | -0.34 (-0.36, -0.47) | 1.04 (-0.33, 1.95) | 0.12 (-0.87, 0.83) | -0.06 (-1.40, 1.03) |
| Costa Rica | 1.36 (1.07, 1.60) | 0.29 (0.03, 0.52) | 0.05 (-0.23, 0.32) | 0.20 (-7.84, 3.55) | -1.03 (-9.61, 2.96) | -0.86 (-11.63, 3.36) |
| Croatia | 0.47 (0.30, 0.61) | -0.38 (-0.57, -0.25) | -0.91 (-1.08, -0.76) | -2.74 (-22.05, 2.34) | -4.10 (-6.12, -2.01) | -3.69 (-5.12, -1.23) |
| Cuba | 0.68 (0.50, 0.85) | -0.21 (-0.38, -0.05) | -0.52 (-0.72, -0.32) | 0.22 (-6.99, 3.73) | -1.09 (-8.31, 3.01) | -0.99 (-12.05, 3.31) |
| Cyprus | 2.02 (2.06, 1.95) | -0.37 (-0.28, -0.50) | -0.48 (-0.40, -0.57) | -0.95 (-3.53, 1.11) | -1.21 (-2.56, -0.29) | -0.93 (-2.69, 0.36) |
| Czechia | -0.32 (-0.54, -0.12) | -1.43 (-1.67, -1.23) | -1.73 (-1.96, -1.52) | -0.45 (-8.43, 3.26) | -0.87 (-7.82, 2.96) | -0.95 (-10.39, 3.20) |
| C娑斿澅e d'Ivoire | 1.19 (1.13, 1.32) | 0.86 (0.83, 0.94) | 0.79 (0.73, 0.90) | 0.70 (0.05, 1.30) | 0.10 (-0.59, 0.37) | -0.15 (-0.58, 0.24) |
| Democratic People's Republic of Korea | 1.00 (0.83, 1.06) | 0.61 (0.50, 0.59) | 0.58 (0.40, 0.58) | 0.81 (0.82, 1.02) | -0.17 (0.07, -0.30) | -0.09 (-0.38, 0.13) |
| Democratic Republic of the Congo | 0.94 (0.64, 1.18) | 0.65 (0.36, 0.90) | 0.57 (0.33, 0.74) | 2.35 (1.22, 3.33) | 1.79 (0.53, 2.87) | 1.71 (0.88, 2.50) |
| Denmark | -0.33 (-0.70, 0.03) | -1.95 (-2.12, -1.81) | -2.43 (-2.58, -2.28) | -0.70 (-10.79, 3.17) | -0.73 (-1.43, -0.27) | -0.49 (-1.63, 0.46) |
| Djibouti | 1.84 (1.97, 1.99) | 1.28 (1.53, 1.42) | 1.14 (1.18, 1.35) | 2.43 (2.41, 1.79) | 1.62 (2.09, 1.03) | 1.62 (1.52, 0.94) |
| Dominica | 0.72 (0.58, 0.89) | 0.54 (0.44, 0.71) | 0.28 (0.14, 0.46) | 0.12 (-0.54, 0.86) | 0.06 (-0.51, 0.94) | 0.06 (-0.94, 0.83) |
| Dominican Republic | 2.90 (2.40, 3.32) | 1.92 (1.55, 2.22) | 1.89 (1.40, 2.31) | -1.43 (-5.49, 0.93) | -3.26 (-7.98, -0.37) | -3.95 (-9.97, -0.82) |
| Ecuador | 2.66 (2.24, 3.09) | 1.12 (0.78, 1.47) | 1.02 (0.58, 1.45) | 1.19 (-3.23, 4.12) | 0.01 (-5.04, 3.34) | 0.19 (-5.95, 3.62) |
| Egypt | 2.44 (1.65, 3.01) | 1.40 (0.56, 2.01) | 1.22 (0.46, 1.78) | 1.35 (-2.69, 3.49) | -1.41 (-7.90, 1.20) | -1.18 (-7.91, 1.74) |
| El Salvador | 2.73 (2.23, 3.15) | 1.06 (0.61, 1.44) | 0.97 (0.48, 1.37) | 1.52 (-1.56, 3.57) | 0.46 (-2.61, 2.56) | 0.26 (-3.38, 2.52) |
| Equatorial Guinea | 3.60 (2.85, 4.43) | 2.49 (1.92, 3.20) | 2.03 (1.22, 2.89) | 2.14 (1.37, 2.10) | 1.50 (0.68, 1.53) | 1.28 (0.01, 1.02) |
| Eritrea | 2.00 (2.57, 1.85) | 1.45 (1.99, 1.26) | 1.41 (1.92, 1.28) | 1.61 (1.30, 1.90) | 1.13 (0.68, 1.53) | 1.07 (0.71, 1.57) |
| Estonia | 0.76 (0.40, 1.08) | -1.11 (-1.45, -0.80) | -1.50 (-1.86, -1.17) | 0.34 (-6.54, 3.57) | -0.46 (-7.72, 3.07) | -0.21 (-9.04, 3.56) |
| Eswatini | 1.76 (0.84, 2.38) | 1.41 (0.64, 1.89) | 1.42 (0.43, 2.12) | 0.35 (2.09, 0.01) | -0.30 (1.90, -0.31) | -0.47 (1.73, -0.52) |
| Ethiopia | 0.00 (0.41, -0.34) | -0.43 (-0.02, -0.83) | -0.93 (-0.50, -1.22) | 2.14 (1.80, 2.51) | 1.37 (1.48, 0.91) | 1.15 (0.81, 1.17) |
| Fiji | 1.35 (1.30, 1.37) | 1.00 (0.96, 1.03) | 0.88 (0.80, 0.91) | 1.15 (0.41, 2.26) | 0.29 (-0.71, 1.59) | 0.26 (-0.75, 1.46) |
| Finland | 0.71 (0.35, 1.04) | -1.02 (-1.15, -0.91) | -1.27 (-1.41, -1.12) | -0.70 (-12.11, 3.34) | -0.81 (-1.38, -0.04) | -0.60 (-1.47, 0.20) |
| France | 0.39 (0.05, 0.70) | -1.27 (-1.42, -1.15) | -1.34 (-1.48, -1.20) | -0.63 (-11.95, 3.69) | -0.72 (-1.10, -0.38) | -0.78 (-1.69, 0.15) |
| Gabon | 1.09 (0.50, 1.56) | 0.57 (-0.00, 1.01) | 0.35 (-0.23, 0.85) | 1.71 (1.09, 1.58) | 0.84 (0.58, 0.40) | 0.69 (0.38, 0.70) |
| Gambia | -1.09 (-1.14, -1.07) | 1.63 (1.27, 1.92) | 1.65 (1.35, 1.88) | 2.74 (2.17, 2.84) | 2.17 (1.18, 2.52) | 2.20 (1.41, 2.53) |
| Georgia | -0.44 (-0.45, -0.41) | 0.15 (0.12, 0.18) | -0.16 (-0.21, -0.11) | 1.12 (-2.65, 3.39) | -0.31 (-5.48, 2.41) | 0.02 (-5.26, 2.65) |
| Germany | 0.48 (0.11, 0.84) | -0.91 (-1.03, -0.83) | -1.22 (-1.33, -1.11) | -0.71 (-13.00, 3.98) | -0.95 (-1.73, -0.40) | -0.75 (-1.72, -0.02) |
| Ghana | 0.89 (0.85, 0.95) | 0.43 (0.43, 0.49) | 0.31 (0.26, 0.37) | 1.92 (1.58, 2.19) | 0.91 (0.76, 0.96) | 0.61 (0.20, 0.84) |
| Greece | 0.44 (0.14, 0.75) | -0.52 (-0.62, -0.44) | -0.72 (-0.81, -0.62) | 0.96 (-6.99, 4.43) | 0.92 (0.45, 1.37) | 0.71 (0.24, 1.36) |
| Greenland | -1.14 (-1.24, -1.05) | -1.97 (-2.01, -1.88) | -2.00 (-2.09, -1.90) | 0.34 (-1.37, 1.47) | -0.26 (-2.21, 0.83) | -0.29 (-2.81, 1.21) |
| Grenada | 1.33 (1.31, 1.34) | 0.76 (0.75, 0.76) | 0.51 (0.50, 0.53) | 0.66 (-0.27, 0.99) | -0.65 (-1.31, -0.35) | -0.87 (-1.96, -0.04) |
| Guam | 0.69 (0.58, 0.83) | -0.05 (-0.15, 0.05) | 0.48 (0.41, 0.59) | 1.35 (0.41, 2.25) | 1.25 (0.53, 2.37) | 0.91 (-0.02, 1.85) |
| Guatemala | 2.08 (1.94, 2.26) | 0.69 (0.58, 0.80) | 0.59 (0.49, 0.69) | -0.04 (-3.45, 2.41) | -1.13 (-4.52, 1.69) | -1.05 (-5.46, 1.88) |
| Guinea | 1.11 (0.74, 1.48) | 0.75 (0.45, 1.08) | 0.74 (0.33, 1.18) | 0.67 (-0.06, 0.90) | 0.14 (-0.53, 0.22) | -0.01 (-0.86, 0.28) |
| Guinea-Bissau | 1.39 (1.35, 1.74) | 1.01 (1.00, 1.35) | 0.94 (0.92, 1.26) | 0.28 (-0.36, 0.75) | -0.13 (-0.83, 0.48) | -0.34 (-0.89, -0.01) |
| Guyana | 0.96 (0.67, 1.15) | 0.40 (0.16, 0.56) | 0.57 (0.29, 0.75) | 0.29 (-1.76, 1.48) | -0.91 (-2.64, 0.61) | -1.14 (-3.81, 0.65) |
| Haiti | 0.91 (0.10, 1.51) | 0.44 (-0.29, 0.98) | 0.37 (-0.44, 0.91) | 0.38 (0.41, -0.02) | -0.13 (0.02, -0.30) | -0.27 (0.07, -0.39) |
| Honduras | 2.21 (1.76, 2.61) | 1.35 (1.08, 1.65) | 0.96 (0.49, 1.39) | 0.87 (1.69, 0.48) | -0.45 (0.61, -1.47) | -0.05 (0.99, -1.12) |
| Hungary | -0.22 (-0.40, -0.02) | -1.30 (-1.45, -1.16) | -1.51 (-1.70, -1.32) | -0.10 (-7.93, 3.52) | -1.33 (-10.87, 2.82) | -1.11 (-11.78, 3.19) |
| Iceland | -1.29 (-1.50, -1.13) | -2.14 (-2.34, -2.01) | -2.35 (-2.50, -2.22) | -1.09 (-2.34, -0.18) | -2.07 (-2.66, -1.46) | -1.67 (-2.41, -1.27) |
| India | 1.66 (1.61, 1.72) | 0.63 (0.60, 0.70) | 0.72 (0.65, 0.79) | 1.67 (-0.49, 3.10) | 0.73 (-1.63, 2.37) | 0.52 (-2.09, 2.36) |
| Indonesia | 1.31 (0.88, 1.66) | 0.52 (0.12, 0.82) | 0.43 (0.03, 0.76) | 0.84 (0.49, 1.75) | -0.15 (-0.65, 0.81) | -0.48 (-0.65, 0.33) |
| Iran (Islamic Republic of) | 1.87 (2.25, 1.38) | 0.48 (0.93, -0.11) | 0.51 (0.87, 0.01) | 1.96 (1.43, 2.40) | 0.08 (-0.67, 0.84) | 0.02 (-0.87, 0.61) |
| Iraq | 2.06 (2.18, 2.03) | 0.64 (0.79, 0.53) | 0.57 (0.61, 0.56) | 1.95 (2.35, 1.82) | 0.53 (1.14, 0.17) | 0.09 (0.64, 0.06) |
| Ireland | 0.18 (-0.14, 0.47) | -1.58 (-1.71, -1.47) | -1.89 (-2.02, -1.75) | -1.02 (-12.63, 2.83) | -1.52 (-2.51, -0.69) | -1.35 (-2.58, -0.04) |
| Israel | -0.39 (-0.68, -0.13) | -1.90 (-2.02, -1.82) | -2.06 (-2.16, -1.98) | 0.24 (-8.67, 3.84) | -0.07 (-0.27, 0.25) | -0.22 (-0.97, 0.50) |
| Italy | -0.16 (-0.47, 0.10) | -1.25 (-1.44, -1.14) | -1.52 (-1.63, -1.42) | -0.52 (-12.08, 3.73) | -0.56 (-0.79, -0.21) | -0.64 (-1.11, -0.15) |
| Jamaica | 2.12 (1.97, 2.23) | 1.16 (0.98, 1.30) | 1.49 (1.35, 1.57) | -0.44 (-4.85, 1.95) | -0.50 (-4.43, 1.76) | -1.23 (-6.00, 1.65) |
| Japan | 2.05 (1.88, 2.21) | 0.62 (0.43, 0.72) | 0.49 (0.39, 0.59) | -1.54 (-12.27, 2.83) | -1.78 (-1.93, -1.27) | -1.62 (-2.01, -0.93) |
| Jordan | 1.33 (1.49, 1.20) | -0.35 (-0.18, -0.52) | -0.66 (-0.56, -0.78) | 0.76 (-1.20, 2.19) | -0.23 (-2.15, 1.24) | -0.13 (-2.02, 1.51) |
| Kazakhstan | 1.06 (0.93, 1.19) | -0.04 (-0.13, 0.05) | -0.41 (-0.52, -0.29) | -0.03 (-4.11, 2.22) | -1.84 (-6.74, 0.88) | -1.51 (-6.78, 1.46) |
| Kenya | 1.65 (1.92, 1.42) | 1.70 (1.94, 1.49) | 1.64 (1.94, 1.40) | 2.15 (1.82, 1.84) | 1.00 (1.09, 1.20) | 0.81 (0.67, 1.22) |
| Kiribati | 0.58 (0.13, 1.11) | 0.38 (0.01, 0.89) | 0.19 (-0.30, 0.69) | 1.14 (1.67, 1.75) | 0.61 (1.06, 1.28) | 0.50 (0.74, 1.00) |
| Kuwait | 0.50 (0.25, 0.85) | -0.51 (-0.73, -0.19) | -1.04 (-1.27, -0.67) | 0.63 (-1.82, 1.94) | -0.26 (-2.76, 1.00) | -0.26 (-3.27, 1.38) |
| Kyrgyzstan | -1.57 (-1.62, -1.53) | -2.16 (-2.18, -2.15) | -2.58 (-2.61, -2.54) | 0.06 (-3.32, 2.36) | -1.60 (-5.75, 1.26) | -1.32 (-5.80, 1.65) |
| Lao People's Democratic Republic | 0.58 (0.90, 0.59) | -0.12 (0.16, -0.11) | -0.31 (0.09, -0.31) | 1.25 (0.93, 0.86) | 0.40 (-0.41, 0.09) | 0.16 (-0.20, -0.23) |
| Latvia | 0.68 (0.24, 1.10) | -0.43 (-0.84, -0.03) | -0.97 (-1.43, -0.52) | 0.29 (-5.34, 3.45) | -1.13 (-9.85, 2.64) | -0.74 (-9.90, 2.98) |
| Lebanon | 3.76 (3.59, 3.94) | 1.40 (1.19, 1.62) | 1.26 (1.15, 1.44) | 1.10 (-0.06, 1.81) | -0.07 (-0.95, 0.80) | 0.10 (-1.31, 1.00) |
| Lesotho | 3.94 (3.33, 4.35) | 3.58 (3.06, 3.94) | 3.83 (3.13, 4.20) | -0.43 (-0.37, -0.53) | -1.15 (-2.04, -1.22) | -1.20 (-0.82, -1.33) |
| Liberia | 1.46 (0.95, 2.16) | 0.94 (0.54, 1.58) | 0.79 (0.32, 1.47) | 0.83 (-0.21, 1.41) | 0.37 (-0.62, 0.97) | 0.31 (-0.93, 0.95) |
| Libya | 2.52 (2.12, 2.73) | 1.32 (1.00, 1.50) | 1.27 (0.91, 1.49) | 0.65 (-1.02, 1.67) | 0.51 (-0.57, 1.47) | 0.51 (-0.76, 1.49) |
| Lithuania | 0.27 (0.07, 0.47) | -0.47 (-0.68, -0.26) | -0.80 (-1.02, -0.57) | -1.39 (-11.29, 2.61) | -2.62 (-16.53, 2.21) | -2.54 (-19.79, 2.55) |
| Luxembourg | -0.30 (-0.59, -0.02) | -1.66 (-1.88, -1.45) | -1.90 (-2.10, -1.69) | -2.33 (-5.81, -0.11) | -1.98 (-3.13, -0.81) | -2.00 (-3.37, -0.99) |
| Madagascar | 0.72 (0.22, 1.18) | 0.43 (0.04, 0.87) | 0.31 (-0.17, 0.75) | 1.68 (0.79, 2.15) | 1.21 (0.33, 1.53) | 1.09 (0.45, 1.27) |
| Malawi | 1.29 (1.30, 1.26) | 0.99 (1.05, 0.94) | 0.56 (0.49, 0.55) | 1.60 (0.99, 2.08) | 1.18 (0.75, 1.70) | 1.12 (0.49, 1.78) |
| Malaysia | 1.94 (1.69, 2.14) | 0.48 (0.27, 0.62) | 0.41 (0.18, 0.59) | 2.41 (-0.79, 3.88) | 1.42 (-2.34, 3.19) | 1.28 (-2.37, 3.03) |
| Maldives | 0.31 (1.59, -0.49) | -1.73 (-0.59, -2.49) | -2.15 (-0.82, -2.98) | 1.80 (0.95, 2.32) | 0.80 (0.26, 1.76) | 0.74 (0.05, 1.64) |
| Mali | 0.65 (0.37, 0.91) | 0.24 (0.04, 0.43) | 0.06 (-0.27, 0.31) | 1.22 (0.96, 1.85) | 0.64 (0.53, 1.51) | 0.68 (0.22, 1.18) |
| Malta | -0.18 (-0.41, 0.03) | -1.79 (-1.98, -1.61) | -1.76 (-1.94, -1.58) | -0.34 (-2.48, 1.34) | -1.68 (-3.05, -0.32) | -1.30 (-2.52, -0.04) |
| Marshall Islands | 1.93 (1.14, 2.59) | 1.46 (0.79, 2.05) | 1.49 (0.70, 2.17) | 0.87 (0.73, 1.21) | 0.33 (0.26, 0.68) | 0.22 (0.22, 0.41) |
| Mauritania | 0.76 (0.91, 0.62) | 0.15 (0.34, -0.02) | -0.04 (0.12, -0.21) | 0.97 (-1.60, 2.15) | 0.05 (-2.62, 1.08) | -0.21 (-3.82, 1.26) |
| Mauritius | 3.09 (2.96, 3.19) | 2.08 (2.00, 2.15) | 2.20 (2.09, 2.29) | 0.78 (-5.37, 3.76) | -0.20 (-6.65, 3.27) | -0.31 (-8.53, 3.27) |
| Mexico | 1.48 (1.29, 1.65) | 0.15 (-0.02, 0.31) | 0.16 (-0.00, 0.32) | 0.91 (-6.84, 4.78) | -0.43 (-8.43, 3.91) | -0.36 (-9.04, 3.94) |
| Micronesia (Federated States of) | 2.00 (1.09, 2.93) | 1.27 (0.63, 2.06) | 1.22 (0.26, 2.19) | 1.30 (0.06, 1.63) | 0.76 (-0.12, 1.10) | 0.63 (-1.04, 0.94) |
| Monaco | 1.62 (1.74, 1.47) | 0.51 (0.70, 0.26) | 0.46 (0.60, 0.29) | -1.55 (-3.96, -0.70) | -1.66 (-3.85, -0.66) | -1.66 (-3.46, -0.60) |
| Mongolia | 1.04 (0.96, 1.11) | 0.14 (0.10, 0.16) | -0.26 (-0.34, -0.20) | 1.55 (0.27, 2.21) | 0.55 (-0.65, 1.42) | 0.67 (-0.59, 1.56) |
| Montenegro | 1.46 (1.45, 1.38) | 0.46 (0.47, 0.40) | 0.40 (0.41, 0.32) | -0.19 (-2.46, 0.93) | -1.45 (-3.86, -0.00) | -0.99 (-3.59, 0.66) |
| Morocco | 2.16 (1.63, 2.70) | 1.01 (0.56, 1.50) | 0.92 (0.42, 1.47) | 1.89 (1.46, 2.31) | 0.23 (-0.49, 0.83) | 0.11 (-0.39, 0.50) |
| Mozambique | 1.91 (1.43, 2.27) | 1.47 (1.07, 1.81) | 1.40 (0.90, 1.74) | 1.43 (0.81, 2.45) | 0.95 (0.23, 1.93) | 0.83 (-0.40, 1.92) |
| Myanmar | -2.27 (-1.61, -2.73) | -2.86 (-2.28, -3.32) | -3.41 (-2.72, -3.88) | -12.06 (-12.96, -10.89) | -16.29 (-18.79, -13.84) | -39.43 (-41.42, -28.82) |
| Namibia | 3.24 (2.91, 3.74) | 2.13 (1.88, 2.51) | 2.21 (1.93, 2.71) | 0.48 (0.62, 1.11) | -0.46 (-0.42, -0.08) | -0.71 (-0.63, 0.27) |
| Nauru | 1.54 (1.61, 1.55) | 1.08 (1.43, 1.01) | 1.05 (0.96, 1.10) | 1.42 (0.72, 1.69) | -0.15 (-1.32, 0.25) | -0.09 (-0.84, -0.03) |
| Nepal | 1.93 (2.29, 1.80) | 1.06 (1.48, 0.91) | 0.88 (1.18, 0.72) | 2.00 (1.21, 2.49) | 1.07 (0.14, 1.49) | 0.95 (0.16, 1.70) |
| Netherlands | -0.15 (-0.43, 0.15) | -1.26 (-1.39, -1.15) | -1.63 (-1.75, -1.50) | 0.44 (-7.21, 4.06) | -0.13 (-0.43, 0.21) | 0.18 (-0.59, 0.92) |
| New Zealand | -0.18 (-0.50, 0.12) | -1.72 (-1.82, -1.65) | -1.79 (-1.88, -1.71) | -0.16 (-9.32, 3.73) | -0.16 (-0.47, 0.33) | -0.29 (-0.97, 0.40) |
| Nicaragua | 3.11 (3.19, 3.08) | 1.45 (1.58, 1.37) | 1.11 (1.21, 1.05) | 1.13 (-3.23, 4.15) | -0.19 (-4.44, 2.73) | -0.29 (-7.27, 3.62) |
| Niger | 0.51 (0.37, 0.89) | 0.11 (0.01, 0.40) | -0.04 (-0.23, 0.36) | 0.95 (0.90, 1.49) | 0.59 (0.73, 0.92) | 0.68 (0.54, 1.07) |
| Nigeria | 1.84 (1.85, 1.76) | 1.24 (1.33, 1.02) | 1.13 (1.21, 0.97) | 0.68 (0.07, 0.62) | 0.19 (0.09, 0.66) | -0.11 (-0.28, -0.31) |
| Niue | 1.68 (1.23, 1.81) | 0.58 (0.16, 0.63) | 0.49 (0.09, 0.61) | 0.54 (-0.28, 0.70) | -0.09 (-0.87, -0.03) | -0.07 (-1.41, -0.01) |
| North Macedonia | 1.50 (1.18, 1.79) | 0.42 (0.24, 0.59) | -0.11 (-0.43, 0.17) | 0.25 (-6.32, 3.28) | -0.62 (-7.03, 2.34) | -0.50 (-8.01, 2.52) |
| Northern Mariana Islands | 0.30 (0.46, 0.27) | -0.14 (0.03, -0.19) | -0.24 (-0.08, -0.25) | 0.42 (-0.49, 0.69) | -0.62 (-1.59, 0.19) | -0.65 (-1.50, 0.04) |
| Norway | 0.11 (-0.12, 0.39) | -1.68 (-1.83, -1.52) | -1.95 (-2.07, -1.77) | -2.16 (-14.01, 2.56) | -1.57 (-1.70, -1.24) | -1.65 (-2.15, -0.99) |
| Oman | 3.02 (3.83, 2.26) | 1.41 (2.11, 0.76) | 1.19 (1.98, 0.45) | 0.34 (-0.88, 1.01) | -1.64 (-2.81, -0.90) | -1.04 (-2.51, 0.07) |
| Pakistan | 1.96 (1.96, 1.89) | 1.29 (1.33, 1.20) | 1.30 (1.24, 1.34) | 1.15 (0.63, 1.71) | 0.43 (0.43, 1.09) | 0.28 (0.22, 1.23) |
| Palau | 1.08 (1.12, 1.07) | 0.36 (0.43, 0.29) | 0.31 (0.34, 0.31) | 0.95 (0.63, 1.19) | 0.21 (-0.09, 0.40) | 0.15 (-0.02, 0.10) |
| Palestine | 1.90 (2.62, 1.11) | 1.03 (1.73, 0.25) | 0.84 (1.56, 0.07) | 2.56 (0.84, 3.69) | 1.25 (-0.63, 2.62) | 1.32 (-0.75, 2.51) |
| Panama | 1.39 (1.16, 1.58) | 0.37 (0.17, 0.54) | 0.32 (0.08, 0.51) | 0.45 (-8.89, 3.80) | -1.89 (-15.32, 2.63) | -1.76 (-23.18, 2.97) |
| Papua New Guinea | 0.89 (0.87, 0.97) | 0.59 (0.57, 0.65) | 0.57 (0.60, 0.65) | 0.99 (1.06, 1.30) | 0.49 (0.58, 0.74) | 0.45 (0.24, 0.45) |
| Paraguay | 2.16 (1.98, 2.29) | 1.08 (0.94, 1.19) | 0.98 (0.81, 1.11) | 1.95 (-2.08, 3.80) | 0.33 (-4.28, 2.66) | 0.60 (-4.53, 2.95) |
| Peru | 0.81 (0.42, 1.14) | -0.91 (-1.23, -0.66) | -1.09 (-1.49, -0.78) | 1.35 (-0.35, 2.72) | 0.03 (-1.75, 1.56) | 0.13 (-1.99, 1.49) |
| Philippines | 0.14 (-0.14, 0.45) | -0.53 (-0.79, -0.25) | -0.22 (-0.51, 0.08) | 0.87 (-2.18, 2.73) | 0.14 (-3.21, 2.23) | -0.04 (-3.98, 2.11) |
| Poland | 1.13 (0.79, 1.45) | -0.52 (-0.78, -0.28) | -0.75 (-1.05, -0.45) | -0.88 (-12.07, 3.40) | -1.26 (-10.89, 3.28) | -1.37 (-13.06, 3.13) |
| Portugal | 0.25 (-0.10, 0.57) | -1.62 (-1.77, -1.53) | -1.74 (-1.87, -1.61) | 0.25 (-9.14, 4.07) | -0.43 (-0.73, 0.50) | -0.25 (-1.03, 0.88) |
| Puerto Rico | 1.04 (0.70, 1.33) | -0.48 (-0.81, -0.19) | -0.57 (-0.91, -0.26) | 0.79 (-6.29, 4.16) | 0.65 (-6.33, 4.12) | 0.72 (-7.88, 4.26) |
| Qatar | 3.27 (3.33, 3.12) | 1.35 (1.55, 1.07) | 0.52 (0.61, 0.38) | -1.20 (-1.82, -0.06) | -2.77 (-3.90, -1.70) | -2.56 (-3.56, -1.67) |
| Republic of Korea | 3.67 (3.42, 3.88) | 0.63 (0.44, 0.73) | 0.53 (0.36, 0.65) | 0.37 (-4.05, 2.78) | -0.33 (-1.35, 0.68) | -0.30 (-1.92, 0.89) |
| Republic of Moldova | 0.25 (0.14, 0.37) | -0.47 (-0.54, -0.37) | -1.02 (-1.11, -0.88) | -4.66 (-12.68, -0.06) | -6.89 (-24.57, -1.36) | -7.52 (-8.21, -6.41) |
| Romania | 1.27 (1.07, 1.45) | 0.08 (-0.10, 0.24) | -0.26 (-0.44, -0.08) | 0.37 (-6.74, 3.77) | -0.81 (-10.22, 3.48) | -0.74 (-12.13, 3.61) |
| Russian Federation | 1.30 (1.18, 1.44) | -0.04 (-0.16, 0.07) | -0.35 (-0.47, -0.21) | -0.75 (-14.90, 3.92) | -1.48 (-14.92, 3.11) | -1.59 (-14.81, 3.11) |
| Rwanda | -0.11 (-0.06, -0.02) | -0.65 (-0.55, -0.60) | -1.17 (-1.13, -1.06) | 2.01 (2.12, 2.30) | 1.32 (1.72, 1.27) | 1.22 (1.61, 1.38) |
| Saint Kitts and Nevis | -0.37 (-0.90, -0.01) | -0.92 (-1.25, -0.65) | -1.47 (-2.10, -1.08) | 0.31 (-1.52, 1.80) | -0.62 (-2.15, 0.97) | -0.75 (-3.11, 1.03) |
| Saint Lucia | -0.07 (-0.22, 0.10) | -1.05 (-1.18, -0.90) | -0.98 (-1.15, -0.81) | 0.94 (0.07, 2.11) | 0.44 (-1.19, 1.47) | -0.05 (-1.73, 1.13) |
| Saint Vincent and the Grenadines | 0.55 (0.49, 0.60) | 0.18 (0.11, 0.24) | -0.01 (-0.07, 0.06) | 0.20 (-1.17, 1.60) | -1.00 (-2.70, 0.40) | -0.81 (-2.54, 0.76) |
| Samoa | 1.05 (0.66, 1.71) | 0.48 (0.15, 1.12) | 0.52 (0.14, 1.19) | 1.73 (1.58, 1.05) | 0.56 (0.67, -0.35) | 0.44 (0.14, -0.65) |
| San Marino | 1.33 (0.86, 1.88) | 0.25 (-0.70, 1.16) | 0.44 (-0.51, 1.36) | -0.31 (-0.96, 0.17) | -0.73 (-1.54, -0.30) | -0.51 (-0.69, 0.51) |
| Sao Tome and Principe | 2.35 (1.92, 2.97) | 1.54 (1.04, 2.12) | 1.48 (1.17, 2.01) | 1.70 (0.28, 2.00) | 0.76 (-0.86, 1.26) | 0.63 (-0.87, 1.03) |
| Saudi Arabia | 3.67 (3.72, 3.62) | 0.54 (0.71, 0.41) | 0.54 (0.73, 0.39) | 1.66 (0.30, 2.91) | -0.11 (-2.22, 1.51) | 0.16 (-1.84, 1.63) |
| Senegal | 1.25 (1.23, 1.35) | 0.87 (0.88, 0.96) | 0.69 (0.69, 0.77) | 1.21 (-0.79, 1.75) | 0.56 (-2.05, 1.38) | 0.44 (-2.61, 1.13) |
| Serbia | 1.39 (1.44, 1.37) | 0.16 (0.19, 0.13) | -0.29 (-0.24, -0.32) | 0.11 (-8.58, 3.84) | -0.53 (-8.90, 3.18) | -0.39 (-9.69, 3.72) |
| Seychelles | 2.69 (2.63, 2.74) | 1.58 (1.51, 1.61) | 1.45 (1.42, 1.46) | 1.33 (-0.05, 2.15) | 0.04 (-1.51, 1.13) | -0.08 (-1.96, 1.28) |
| Sierra Leone | 1.99 (1.94, 2.37) | 1.48 (1.46, 1.84) | 1.58 (1.54, 1.93) | 1.45 (1.06, 2.02) | 0.81 (0.48, 1.75) | 0.85 (0.44, 1.28) |
| Singapore | -0.87 (-0.87, -0.87) | -0.60 (-0.71, -0.55) | -0.78 (-0.84, -0.67) | -1.73 (-11.92, 2.41) | -2.51 (-3.46, -1.53) | -2.27 (-3.62, -1.13) |
| Slovakia | 0.98 (0.49, 1.40) | -0.33 (-0.68, 0.00) | -0.76 (-1.20, -0.36) | -0.49 (-11.04, 3.38) | -1.49 (-12.76, 2.79) | -1.16 (-12.50, 3.23) |
| Slovenia | -0.12 (0.19, -0.36) | -1.43 (-1.24, -1.61) | -1.77 (-1.49, -2.03) | -0.24 (-7.65, 3.85) | -0.80 (-8.16, 3.48) | -0.64 (-9.06, 3.69) |
| Solomon Islands | 6.43 (7.43, 6.01) | 5.12 (6.12, 4.68) | 5.96 (6.86, 5.55) | 7.25 (7.07, 7.39) | 6.87 (6.81, 6.90) | 7.09 (6.93, 7.18) |
| Somalia | 0.51 (0.27, 0.76) | 0.45 (0.30, 0.70) | 0.32 (0.18, 0.55) | 0.35 (0.17, 0.31) | 0.24 (0.56, -0.24) | 0.14 (0.22, -0.12) |
| South Africa | 0.92 (0.92, 0.95) | 0.44 (0.53, 0.36) | 0.18 (0.15, 0.27) | -4.21 (-6.93, -2.76) | -5.33 (-8.29, -3.33) | -5.35 (-8.07, -4.42) |
| South Sudan | 0.35 (0.00, 0.63) | 0.17 (-0.25, 0.45) | 0.02 (-0.45, 0.34) | 1.37 (1.24, 1.21) | 0.99 (1.20, 0.57) | 0.99 (1.03, 0.73) |
| Spain | 0.02 (-0.39, 0.38) | -1.51 (-1.64, -1.42) | -1.84 (-1.96, -1.73) | 0.01 (-8.59, 3.96) | -0.11 (-0.23, 0.34) | -0.14 (-0.91, 0.60) |
| Sri Lanka | 2.82 (2.22, 3.40) | 1.22 (0.71, 1.70) | 1.06 (0.47, 1.63) | 1.21 (0.11, 1.97) | -0.37 (-2.15, 0.59) | -0.19 (-1.73, 0.64) |
| Sudan | 1.95 (1.89, 1.69) | 1.02 (1.15, 0.74) | 0.88 (0.74, 0.76) | 2.67 (2.49, 2.41) | 1.29 (1.47, 0.61) | 1.17 (0.95, 0.68) |
| Suriname | 1.10 (1.03, 1.16) | 0.49 (0.43, 0.53) | 0.39 (0.35, 0.45) | 1.55 (-0.22, 2.87) | 0.80 (-1.13, 2.08) | 0.88 (-1.15, 2.32) |
| Sweden | 0.29 (0.10, 0.48) | -0.59 (-0.68, -0.52) | -0.94 (-1.02, -0.87) | -1.63 (-13.35, 2.78) | -1.82 (-2.57, -1.07) | -1.85 (-2.72, -1.24) |
| Switzerland | -0.80 (-1.15, -0.48) | -1.90 (-2.05, -1.79) | -2.27 (-2.41, -2.13) | -0.32 (-10.46, 3.68) | -0.73 (-1.50, -0.24) | -0.51 (-1.24, 0.21) |
| Syrian Arab Republic | 2.34 (2.23, 2.57) | 0.85 (0.77, 1.06) | 0.59 (0.48, 0.83) | 0.99 (0.14, 1.12) | 0.13 (-0.81, 0.48) | 0.24 (-0.11, 0.72) |
| Taiwan (Province of China) | 2.91 (2.50, 3.33) | 0.94 (0.59, 1.28) | 0.89 (0.58, 1.22) | -1.17 (-15.76, 3.70) | -1.97 (-18.24, 3.30) | -1.85 (-19.46, 3.51) |
| Tajikistan | 0.24 (0.01, 0.44) | 0.16 (-0.09, 0.37) | -0.45 (-0.67, -0.26) | 0.69 (-2.55, 2.80) | -0.37 (-3.69, 1.99) | 0.05 (-3.87, 2.47) |
| Thailand | 1.98 (1.67, 2.20) | 0.18 (-0.07, 0.32) | 0.15 (-0.15, 0.33) | 2.02 (-1.95, 4.33) | 0.75 (-4.44, 3.49) | 0.92 (-4.16, 3.76) |
| Timor-Leste | 1.74 (1.51, 1.87) | 0.88 (0.76, 1.02) | 0.76 (0.40, 0.90) | 1.14 (1.72, 0.86) | 0.80 (1.32, 0.64) | 0.83 (1.27, 0.48) |
| Togo | 0.84 (0.59, 1.18) | 0.46 (0.28, 0.76) | 0.37 (0.08, 0.72) | 1.93 (1.92, 2.16) | 1.17 (1.32, 1.52) | 1.13 (1.20, 1.21) |
| Tokelau | 1.84 (2.33, 1.70) | 0.85 (1.33, 0.67) | 0.73 (1.17, 0.59) | 1.50 (1.19, 1.94) | 0.51 (0.12, 0.73) | 0.43 (-0.02, 0.78) |
| Tonga | 0.65 (0.36, 0.87) | 0.23 (-0.03, 0.41) | 0.07 (-0.23, 0.30) | 1.34 (0.81, 1.51) | 0.60 (0.11, 1.01) | 0.53 (0.06, 1.10) |
| Trinidad and Tobago | 0.29 (-0.44, 0.96) | -0.60 (-1.25, 0.02) | -0.66 (-1.40, -0.01) | 0.28 (-0.30, 0.80) | -0.43 (-0.89, -0.17) | -0.43 (-1.04, -0.09) |
| Tunisia | -0.89 (-0.40, -1.25) | 0.21 (-0.44, 0.63) | 0.32 (-0.42, 0.83) | 1.50 (1.68, 1.46) | -0.04 (0.22, 0.02) | 0.06 (0.29, -0.01) |
| Turkey | 2.55 (2.68, 2.37) | 0.28 (0.45, 0.07) | -0.06 (0.09, -0.26) | 1.34 (-1.16, 2.92) | -0.32 (-3.21, 1.47) | -0.31 (-3.70, 1.67) |
| Turkmenistan | 1.22 (0.87, 1.57) | 0.07 (-0.22, 0.35) | 0.12 (-0.22, 0.46) | -1.46 (-6.51, 2.07) | -2.40 (-8.70, 1.10) | -3.20 (-10.64, 1.13) |
| Tuvalu | 1.26 (0.77, 1.79) | 0.66 (0.15, 1.20) | 0.53 (0.06, 1.05) | 1.20 (1.05, 1.23) | 0.44 (0.08, 0.13) | 0.26 (0.26, 0.23) |
| Uganda | 1.76 (1.84, 1.61) | 1.14 (1.20, 1.00) | 1.13 (1.26, 0.99) | 1.86 (1.09, 2.47) | 1.17 (0.59, 2.06) | 1.01 (0.11, 1.96) |
| Ukraine | -0.86 (-1.12, -0.60) | -1.55 (-1.79, -1.32) | -1.84 (-2.10, -1.58) | 0.69 (-7.86, 4.24) | 0.55 (-8.43, 4.30) | 0.42 (-9.02, 4.19) |
| United Arab Emirates | 1.13 (1.23, 0.91) | 0.33 (0.53, 0.07) | 0.21 (0.27, 0.05) | 1.18 (1.15, 1.42) | 0.01 (-0.23, 0.45) | -0.18 (-0.20, 0.10) |
| United Kingdom | -0.26 (-0.60, 0.08) | -1.65 (-1.75, -1.57) | -1.98 (-2.06, -1.89) | 0.11 (-9.47, 4.13) | 0.10 (-0.03, 0.20) | 0.04 (-0.20, 0.47) |
| United Republic of Tanzania | 1.62 (1.65, 1.61) | 1.09 (1.14, 1.06) | 1.19 (1.23, 1.17) | 2.14 (1.39, 2.42) | 1.38 (0.46, 1.44) | 1.27 (0.23, 1.79) |
| United States of America | -0.98 (-1.17, -0.77) | -1.48 (-1.55, -1.41) | -1.76 (-1.82, -1.70) | -0.28 (-9.60, 4.08) | -0.12 (-0.25, 0.11) | -0.29 (-0.64, 0.13) |
| United States Virgin Islands | 0.60 (0.42, 0.79) | -0.16 (-0.32, -0.05) | -0.38 (-0.57, -0.17) | -0.45 (-1.44, 0.22) | -0.41 (-1.28, 0.44) | -0.26 (-1.60, 0.09) |
| Uruguay | -0.03 (-0.31, 0.24) | -0.95 (-1.02, -0.88) | -1.18 (-1.24, -1.10) | 0.30 (-8.83, 3.87) | -0.51 (-0.91, -0.06) | -0.55 (-1.34, 0.41) |
| Uzbekistan | 1.12 (0.96, 1.28) | 0.52 (0.43, 0.62) | 0.21 (0.06, 0.35) | -0.08 (-5.50, 2.68) | -2.12 (-9.46, 1.40) | -1.45 (-8.97, 1.98) |
| Vanuatu | 1.75 (1.66, 2.04) | 1.44 (1.38, 1.69) | 1.47 (1.32, 1.75) | 1.01 (1.12, 1.16) | 0.61 (0.11, 0.35) | 0.57 (0.98, 0.83) |
| Venezuela (Bolivarian Republic of) | 2.13 (1.58, 2.64) | 0.64 (0.16, 1.10) | 0.64 (0.10, 1.15) | 0.01 (-3.75, 2.29) | 0.18 (-3.04, 2.21) | -0.19 (-3.90, 2.36) |
| Viet Nam | 2.55 (2.48, 2.63) | 0.85 (0.78, 0.93) | 0.87 (0.83, 0.92) | 2.23 (1.80, 2.80) | 0.76 (-0.11, 1.75) | 0.74 (0.15, 1.64) |
| Yemen | 2.36 (3.28, 1.51) | 1.41 (2.32, 0.54) | 1.46 (2.30, 0.71) | 1.45 (1.21, 1.73) | 1.43 (1.46, 1.65) | 1.59 (1.29, 1.78) |
| Zambia | 0.66 (0.51, 0.73) | 0.11 (0.03, 0.20) | -0.09 (-0.19, -0.06) | 1.98 (1.83, 2.38) | 1.12 (0.87, 1.57) | 0.89 (0.79, 1.28) |
| Zimbabwe | 2.07 (1.77, 2.34) | 2.53 (2.26, 2.64) | 2.94 (2.63, 3.07) | 0.37 (-0.33, 1.03) | -0.16 (-1.37, 0.77) | -0.19 (-0.94, 0.80) |

**STABLE 7 The Top 10 Countries With the Largest Increase of Age-Standardized Incidence Rate, Death Rate, and Disability Adjusted Life Years Rate of breast cancer Between 2020 and 2030.**

| order | Countries | EAPC of ASIR | Countries | EAPC of ASDR | Countries | EAPC of Age-standardized DALY rate |
| --- | --- | --- | --- | --- | --- | --- |
|  |  | No.(95%CI) |  | No.(95%CI) |  | No.(95%CI) |
| 1 | Solomon Islands | 7.25 (7.07, 7.39) | Solomon Islands | 6.87 (6.81, 6.90) | Solomon Islands | 7.09 (6.93, 7.18) |
| 2 | China | 2.83 (1.29, 4.13) | Gambia | 2.17 (1.18, 2.52) | Gambia | 2.20 (1.41, 2.53) |
| 3 | Gambia | 2.74 (2.17, 2.84) | Angola | 1.85 (0.98, 2.56) | Democratic Republic of the Congo | 1.71 (0.88, 2.50) |
| 4 | Sudan | 2.67 (2.49, 2.41) | Democratic Republic of the Congo | 1.79 (0.53, 2.87) | Angola | 1.69 (0.26, 2.36) |
| 5 | Palestine | 2.56 (0.84, 3.69) | Djibouti | 1.62 (2.09, 1.03) | Djibouti | 1.62 (1.52, 0.94) |
| 6 | Angola | 2.52 (1.57, 3.19) | Equatorial Guinea | 1.50 (0.68, 1.53) | Yemen | 1.59 (1.29, 1.78) |
| 7 | Djibouti | 2.43 (2.41, 1.79) | Yemen | 1.43 (1.46, 1.65) | Palestine | 1.32 (-0.75, 2.51) |
| 8 | Malaysia | 2.41 (-0.79, 3.88) | Malaysia | 1.42 (-2.34, 3.19) | Malaysia | 1.28 (-2.37, 3.03) |
| 9 | Democratic Republic of the Congo | 2.35 (1.22, 3.33) | United Republic of Tanzania | 1.38 (0.46, 1.44) | Equatorial Guinea | 1.28 (0.01, 1.02) |
| 10 | Bangladesh | 2.29 (1.50, 2.75) | Ethiopia | 1.37 (1.48, 0.91) | United Republic of Tanzania | 1.27 (0.23, 1.79) |

**STABLE 8 The Top 10 Countries With the Lowest Increase of Age-Standardized Incidence Rate, Death Rate, and Disability Adjusted Life Years Rate of breast cancer Between 2020 and 2030.**

| order | **Countries** | EAPC of ASIR | **Countries** | EAPC of ASDR | **Countries** | EAPC of Age-standardized DALY rate |
| --- | --- | --- | --- | --- | --- | --- |
|  |  | No.(95%CI) |  | No.(95%CI) |  | No.(95%CI) |
| 1 | Myanmar | -12.06 (-12.96, -10.89) | Myanmar | -16.29 (-18.79, -13.84) | Myanmar | -39.43 (-41.42, -28.82) |
| 2 | Republic of Moldova | -4.66 (-12.68, -0.06) | Republic of Moldova | -6.89 (-24.57, -1.36) | Republic of Moldova | -7.52 (-8.21, -6.41) |
| 3 | South Africa | -4.21 (-6.93, -2.76) | South Africa | -5.33 (-8.29, -3.33) | South Africa | -5.35 (-8.07, -4.42) |
| 4 | Croatia | -2.74 (-22.05, 2.34) | Croatia | -4.10 (-6.12, -2.01) | Dominican Republic | -3.95 (-9.97, -0.82) |
| 5 | Luxembourg | -2.33 (-5.81, -0.11) | Dominican Republic | -3.26 (-7.98, -0.37) | Croatia | -3.69 (-5.12, -1.23) |
| 6 | Norway | -2.16 (-14.01, 2.56) | Qatar | -2.77 (-3.90, -1.70) | Turkmenistan | -3.20 (-10.64, 1.13) |
| 7 | Singapore | -1.73 (-11.92, 2.41) | Lithuania | -2.62 (-16.53, 2.21) | Qatar | -2.56 (-3.56, -1.67) |
| 8 | Austria | -1.65 (-11.30, 2.50) | Singapore | -2.51 (-3.46, -1.53) | Lithuania | -2.54 (-19.79, 2.55) |
| 9 | Sweden | -1.63 (-13.35, 2.78) | Turkmenistan | -2.40 (-8.70, 1.10) | Singapore | -2.27 (-3.62, -1.13) |
| 10 | Monaco | -1.55 (-3.96, -0.70) | Uzbekistan | -2.12 (-9.46, 1.40) | Brazil | -2.23 (-3.36, -1.00) |

**STABLE 9. The Top 10 Countries With the Largest Increase of Age-Standardized Incidence Rate, Death Rate, and Disability Adjusted Life Years Rate of breast cancer Between 1990 and 2019.**

| order | **Countries** | ASIR | **Countries** | ASDR | **Countries** | Age-standardized DALY rate |
| --- | --- | --- | --- | --- | --- | --- |
|  |  | No.(95%CI) |  | No.(95%CI) |  | No.(95%CI) |
| 1 | Solomon Islands | 6.43 (7.43, 6.01) | Solomon Islands | 5.12 (6.12, 4.68) | Solomon Islands | 5.96 (6.86, 5.55) |
| 2 | Lesotho | 3.94 (3.33, 4.35) | Lesotho | 3.58 (3.06, 3.94) | Lesotho | 3.83 (3.13, 4.20) |
| 3 | Lebanon | 3.76 (3.59, 3.94) | Zimbabwe | 2.53 (2.26, 2.64) | Zimbabwe | 2.94 (2.63, 3.07) |
| 4 | Republic of Korea | 3.67 (3.42, 3.88) | Equatorial Guinea | 2.49 (1.92, 3.20) | Namibia | 2.21 (1.93, 2.71) |
| 5 | Saudi Arabia | 3.67 (3.72, 3.62) | Namibia | 2.13 (1.88, 2.51) | Mauritius | 2.20 (2.09, 2.29) |
| 6 | Equatorial Guinea | 3.60 (2.85, 4.43) | Mauritius | 2.08 (2.00, 2.15) | Equatorial Guinea | 2.03 (1.22, 2.89) |
| 7 | Qatar | 3.27 (3.33, 3.12) | Dominican Republic | 1.92 (1.55, 2.22) | Dominican Republic | 1.89 (1.40, 2.31) |
| 8 | Namibia | 3.24 (2.91, 3.74) | Kenya | 1.70 (1.94, 1.49) | Gambia | 1.65 (1.35, 1.88) |
| 9 | Nicaragua | 3.11 (3.19, 3.08) | Bosnia and Herzegovina | 1.63 (1.75, 1.51) | Kenya | 1.64 (1.94, 1.40) |
| 10 | Mauritius | 3.09 (2.96, 3.19) | Gambia | 1.63 (1.27, 1.92) | Sierra Leone | 1.58 (1.54, 1.93) |

**STABLE 10 The Top 10 Countries With the Lowest Increase of Age-Standardized Incidence Rate, Death Rate, and Disability Adjusted Life Years Rate of breast cancer Between 1990 and 2019.**

| order | **Countries** | ASIR | **Countries** | ASDR | **Countries** | Age-standardized DALY rate |
| --- | --- | --- | --- | --- | --- | --- |
|  |  | No.(95%CI) |  | No.(95%CI) |  | No.(95%CI) |
| 1 | Myanmar | -2.27 (-1.61, -2.73) | Myanmar | -2.86 (-2.28, -3.32) | Myanmar | -3.41 (-2.72, -3.88) |
| 2 | Kyrgyzstan | -1.57 (-1.62, -1.53) | Bermuda | -2.62 (-2.94, -2.24) | Bermuda | -2.70 (-2.97, -2.36) |
| 3 | Iceland | -1.29 (-1.50, -1.13) | Kyrgyzstan | -2.16 (-2.18, -2.15) | Kyrgyzstan | -2.58 (-2.61, -2.54) |
| 4 | Greenland | -1.14 (-1.24, -1.05) | Iceland | -2.14 (-2.34, -2.01) | Denmark | -2.43 (-2.58, -2.28) |
| 5 | Gambia | -1.09 (-1.14, -1.07) | Greenland | -1.97 (-2.01, -1.88) | Iceland | -2.35 (-2.50, -2.22) |
| 6 | Bermuda | -1.01 (-1.29, -0.65) | Denmark | -1.95 (-2.12, -1.81) | Switzerland | -2.27 (-2.41, -2.13) |
| 7 | United States of America | -0.98 (-1.17, -0.77) | Switzerland | -1.90 (-2.05, -1.79) | Maldives | -2.15 (-0.82, -2.98) |
| 8 | Tunisia | -0.89 (-0.40, -1.25) | Israel | -1.90 (-2.02, -1.82) | Israel | -2.06 (-2.16, -1.98) |
| 9 | Singapore | -0.87 (-0.87, -0.87) | Malta | -1.79 (-1.98, -1.61) | Greenland | -2.00 (-2.09, -1.90) |
| 10 | Ukraine | -0.86 (-1.12, -0.60) | Maldives | -1.73 (-0.59, -2.49) | United Kingdom | -1.98 (-2.06, -1.89) |
